# Coming to Terms with the Concept of Moving Species Threatened by Climate Change – A Systematic Review of the Terminology and Definitions

**DOI:** 10.1371/journal.pone.0102979

**Published:** 2014-07-23

**Authors:** Maria H. Hällfors, Elina M. Vaara, Marko Hyvärinen, Markku Oksanen, Leif E. Schulman, Helena Siipi, Susanna Lehvävirta

**Affiliations:** 1 Botany Unit, Finnish Museum of Natural History, University of Helsinki, Helsinki, Finland; 2 Department of Behavioural Sciences and Philosophy, University of Turku, Turku, Finland; 3 Turku Institute for Advanced Studies, University of Turku, Turku, Finland; 4 Faculty of Law, University of Lapland, Rovaniemi, Finland; 5 Department of Environmental Sciences, University of Helsinki, Helsinki, Finland; University of Idaho, United States of America

## Abstract

Intentional moving of species threatened by climate change is actively being discussed as a conservation approach. The debate, empirical studies, and policy development, however, are impeded by an inconsistent articulation of the idea. The discrepancy is demonstrated by the varying use of terms, such as *assisted migration*, *assisted colonisation*, or *managed relocation*, and their multiple definitions. Since this conservation approach is novel, and may for instance lead to legislative changes, it is important to aim for terminological consistency. The objective of this study is to analyse the suitability of terms and definitions used when discussing the moving of organisms as a response to climate change. An extensive literature search and review of the material (868 scientific publications) was conducted for finding hitherto used terms (N = 40) and definitions (N = 75), and these were analysed for their suitability. Based on the findings, it is argued that an appropriate term for a conservation approach relating to aiding the movement of organisms harmed by climate change is *assisted migration* defined as follows: *Assisted migration means safeguarding biological diversity through the translocation of representatives of a species or population harmed by climate change to an area outside the indigenous range of that unit where it would be predicted to move as climate changes, were it not for anthropogenic dispersal barriers or lack of time*. The differences between assisted migration and other conservation translocations are also discussed. A wide adoption of the clear and distinctive term and definition provided would allow more focused research on the topic and enable consistent implementation as practitioners could have the same understanding of the concept.

## Introduction

As the effect of climate change on biodiversity is becoming more evident through, e.g., spatial changes in species' suitable areas (e.g., [1]–[4]), translocation of organisms has been proposed to avoid the loss of biodiversity and to complement current conservation strategies. The idea was, to our knowledge, first proposed by Peters and Darling in 1985 [5], and nine years later termed *human-assisted dispersal* [6]. Since then, numerous other terms have been applied, including *assisted migration*, first used by Whitlock and Milspaugh in 2001 [7], *assisted colonisation* first used in 2007 [8], and *managed relocation* in 2009 [9]. In addition, the initial proposal [5] has also been articulated in various ways. Different terms have been used to refer to similar ideas, while one term may be used to denote different ideas.

The debate around the idea (see, e.g., [10]; and responses to [11]: [12]–[17]) has mostly focused on epistemic uncertainty, such as the possible negative effects of introduced species on a focal area, while the linguistic uncertainties involved have been neglected [18]. However, the diversity of terms and their usage predisposes the scientific discussion to confusion; see, e.g., treatments of the concepts of community and stability [19] and diversity indices [20]. Terminological confusion may lead to poor comparison of one study with another and can seriously hamper scientific development. This, in turn, perturbs public discussion and decision-making and, thus, harms efficient application [21]–[23], [19].

We argue that there is an evident risk for confusion as this new conservation approach is being discussed using different terms and definitions – especially since the measure is evaluated in different fields of science and society. Today, mainstream conservation aims at preserving biota within their current range and at protecting nature from human activity (e.g., [24]–[26]). Moving species to new areas will thus require changes in both conservation practises and regulation [27]. In the legal context, definitions often guide the interpretation of law. In some cases, too much or too little flexibility in the definitions of concepts may lead to conflicts when laws are interpreted. For example, in the USA, the legal concept of species defined in the ESA (Endangered Species Act 1973, Pub. L. No. 93–205) has led to problems in conserving some red-listed species. Recent research has shown that the red wolf (*Canis rufus*) is a hybrid species, and as hybrids are not included in the definition of species given in the ESA, some stake-holders are trying to get the red wolf removed from the ESA listings [28]. The opinion of researchers in law, ecology, conservation biology, environmental ethics and other relevant fields, as well as the views of decision-makers and the public about the idea of moving species depends partly on how this idea is described and articulated. With a clear and concise definition the discussion could stay focused and relevant to conservation of biodiversity under climate change.

In this article we examine, through the hitherto proposed terms and definitions, the general idea of *moving organisms in response to climate change* and distinguish from it the more specific idea of *aiding the dispersal of species threatened by climate change*. We scrutinise two aspects of the original articulation of the idea: the term used to designate it and the definition of the term. The idea, the term, and the definition are interdependent as a concise definition enables communicating an idea to others, and a commonly followed terminology is essential to avoid confusion. Thus, one cannot concentrate on only one of the aspects and hope to clarify the whole concept.

Our aim is to recommend the most suitable term denoting the initial idea of *aiding the dispersal of species threatened by climate change*, and to formulate a standard definition of it, which consists of necessary and sufficient conditions to distinguish this idea from other related ones. We also describe the differences between this specific measure and other cases of translocation. We hope to provide a general, yet biologically valid, articulation of the new approach to facilitate discussion and application void of confusion caused by vague and inconsistent articulations or by definitions relating to conceptually other, however seemingly similar, ideas.

## Materials and Methods

### Literature review

To quantify the discussion on the proposed conservation approach and to generate data to analyse the prevailing terminology and definitions, we conducted a literature search. We used the search query ("assisted migration" OR "assisted coloni*ation" OR "managed relocation" OR "human-aided translocation" OR "assisted translocation" AND "climate change") to search for literature published in English up until the end of 2012. These terms represented our initial understanding on which might be the most commonly used terms for the idea. We included”AND climate change” since an omission of it resulted in a large number of hits that were irrelevant to this study.

To attain maximum coverage of the relevant scientific discussion we conducted the search in Google Scholar, ISI Web of Science, Scopus Elsevier, Hein Online and EBSCO (Academic Search Online). Additionally, we searched the reference lists of two review articles [29], [30]. We excluded publications that were irrelevant (i.e., did not discuss moving organisms under climate change) or did not include a specific term for the idea. We did additional searches on new terms that came up through this search, excluding some general terms that are also used in other contexts (like "translocation" or "assisted dispersal") as they proved to generate a large number of irrelevant hits (Table 1).

**Table 1:** Terms used in three or more publications.

Term	Times mentioned
Assisted migration	563
Assisted colonization	121
Managed relocation	94
Facilitated migration	26
Translocation	25
Human assisted migration	22
Assisted dispersal	14
Assisted translocation	8
Artificial translocation	8
Bening introduction	8
Assisted relocation	7
Managed translocation	7
Facilitated dispersal	5
Human assisted dispersal	5
Conservation introduction	4
Human assisted translocation	4
Transformative restoration	3

Other terms (used in one or two publications) are: adaptation assisted migration, assisted afforestation, assisted ecosystem migration, assisted population migration, assisted range expansion, assisted reintroduction, assisted species relocation, facilitated translocation, forestry assisted migration, human aided translocation, human assistance of dispersal, human assisted colonisation, human assisted establishment, human assisted migration management, human assisted relocation, managed migration, migration management, managed reintroduction, planned invasions process, plant refuge translocation, species rescue assisted migration, and trans situ conservation.

We acknowledge that the use of a specific term and definition in a certain publication is not independent from other publications. Quite often, the use of a term or definition in influential papers by highly-cited scientists may promote their adoption by others. Nevertheless, it depicts the actual use of the term. Moreover, all of the clauses we classified as definitions may not have been intended as such by the authors. However, we considered them definition-like articulations and treated them as definitions in this analysis.

### Analysis of terms

For the terminological analysis, we recorded all terms referring to moving species under climate change. When several terms were mentioned in a publication we chose the main one used throughout the text. In cases where several terms were used throughout the text we recorded them all (two to three). Thus, the total number of occurrences of terms is greater than the number of publications reviewed.

The approach was usually referred to using a so called ‘complex term’, which consists of two or more words (e.g., *managed relocation* or *facilitated dispersal*). One of the words (usually a noun) can be understood as the main term that is qualified by restrictive modifying terms (adjectives or adjectival phrases). For instance, in the complex term *assisted colonisation*, the main term is *colonisation* and the modifying term *assisted* singles it out from other instances of colonisation.

Most previous terminological discussion on this new conservation approach has focused on the main term. For example, *colonisation*, *migration*, and *introduction* have been thoroughly discussed by Hunter [31]. However, both the single words and the term as a whole are important when choosing a suitable term. We analysed the meanings of the main and modifying terms separately, as we think that in an emerging field such as this, the meanings of the words comprising a term are easily carried over from previous uses and the complex terms do not have established meanings beyond the meanings of their parts.

### Analysis of definitions

In the analysis of definitions of the measure we included only peer-reviewed articles that in their title, abstract, or keywords mention a relevant term for the general measure. We used content analysis, a method that can be employed to identify patterns across qualitative data by calculating the frequency with which analysis units occur [32]. Our analysis units were single words or parts of sentences used in the definitions.

We followed the three-step view of content analysis by Miles and Huberman [33]. *Reduction* means that the data are selected and simplified by leaving out uninformative words, such as *and*, *or*, *is*. For *data display* (or *grouping*, cf. [34]) we identified similarities and differences of the analysis units and grouped them. Related words (e.g. move, movement, moving) were placed together into subgroups that were used to form larger groups that contain synonyms (e.g., threatened and endangered belong to the same group). Finally, *conclusion drawing* implies finding patterns from the previous steps: we placed groups referring to similar aspects of the approach into the same main category. For example, *moving*, *translocating* and *planting* were placed under the main category *action*.

To identify the exact meanings of the words used in the terms and definitions for the concept, and to evaluate the suitability of each word as part of the term or definition, we relied on interpretations from Oxford English Dictionary [35] and Collins Dictionary and Thesaurus [36]. E.M.V. and M.H.H. initially carried out the content analysis separately. Thereafter, they made a synthesis of their subjective views. Finally, the procedure was re-evaluated by S.L., for conflicting views.

## Results and Discussion

The idea of moving species in response to climate change is discussed in various contexts, for example, in the conservation of species facing a changing climate (cf. [5]) and in choosing the right provenances in forestry (e.g., [37]). Accordingly, the articulations of the idea differ substantially regarding what is to be moved where and why. Moreover, different authors speak about moving different kinds of units, such as individuals, populations, or species. We focus on this issue under *definition review* - *what* and talk about moving *species* until then for simplicity's sake.

Some authors refer to moving species *outside historic ranges* (e.g., [38]) and some *to more favourable regions* (e.g., [39]). Moving individuals beyond the range of the species is the core of many conceptualisation of the idea (e.g., [40]), although sometimes moving within the range is included in the discussion (e.g., [37]). Some authors distinguish between moving species over different distances, e.g. *assisted population migration* for “the movement of species within a species' established range” and *assisted range expansion*, for “the movement of species to areas just outside their established range”; and *assisted long-distance dispersal*, “the movement of species to areas far outside their established range” ([37]; see also [38]).

The motivation for the measure varies from a general anthropogenic threat (e.g., [11]; [40]) and an entailing need for conservation (e.g., [31]; [10]) to more specific reasons, such as managing commercial forests (e.g., [41]; [37]; [42]). IUCN [43] provides a definition of *assisted colonisation* (listing *benign introduction*, *assisted migration*, and *managed relocation* as synonyms) where the motivation for the measure is left open to include any threat to the focal species, not only climate change.

**Figure 1:**
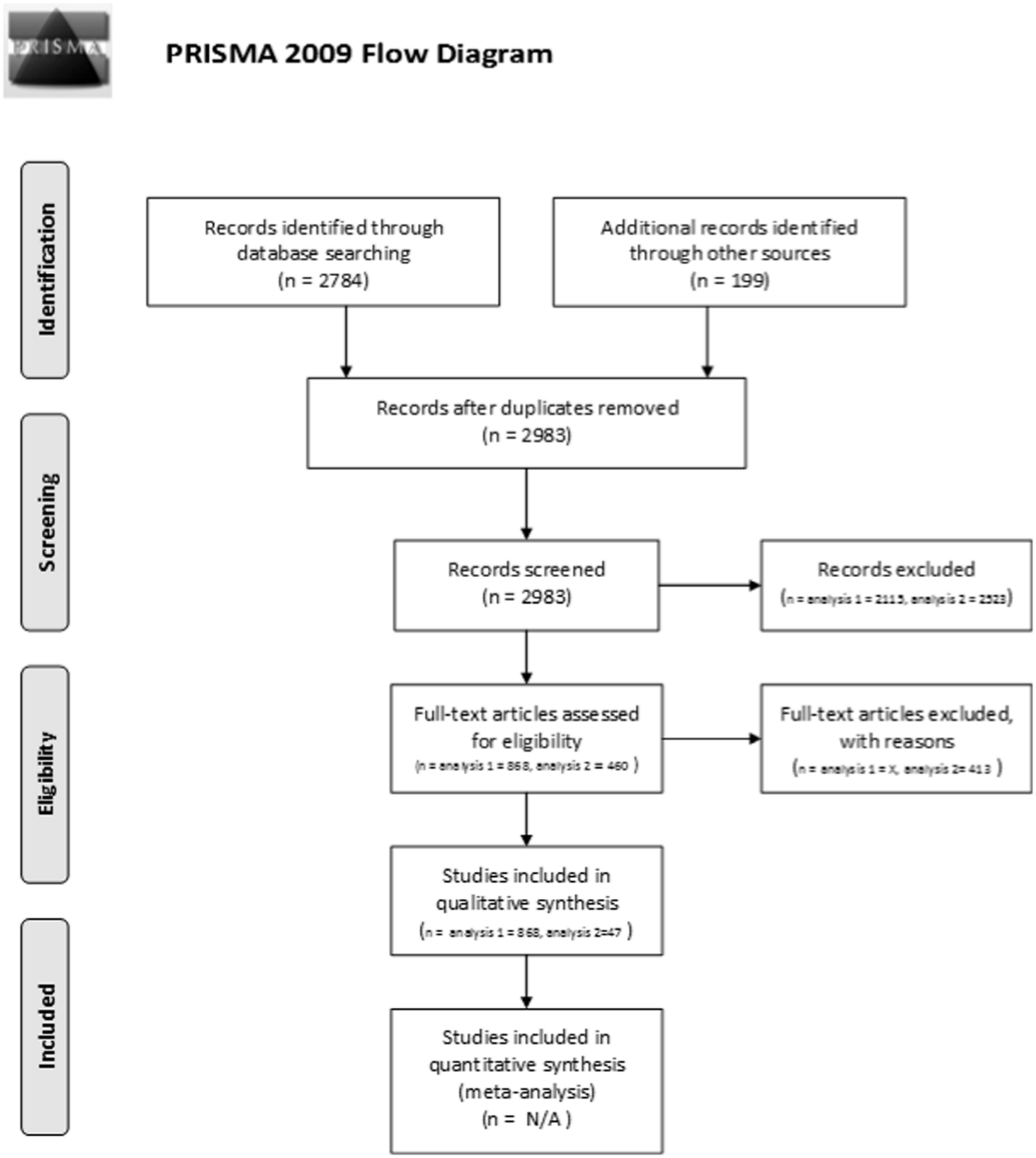
Work flow of the systematic review. ‘Analysis 1’ refers to the data used in the terminological analysis and ‘Analysis 2’ to the definition analysis.

Through our literature search, we found 2983 records (Fig. 1). Of these, 868 mention moving species in connection to climate change using a specific term, and they form our data (Table S1). The data include 460 scientific peer-reviewed articles, 111 reports, 47 theses, 85 books or book chapters, and 165 popular or professional articles including published abstracts of congress presentations. The literature review established that a multitude of terms and definitions are used to describe the idea of *moving species as a response to climate change*. In the following sections we analyse the hitherto proposed definitions and terms to assess their suitability for describing the more specific idea of *aiding the dispersal of species threatened by climate change*. We discuss them by examining the modifying and the main term and the eight main categories identified in the concept analysis of the definitions.

### Terminological review

**Figure 2:**
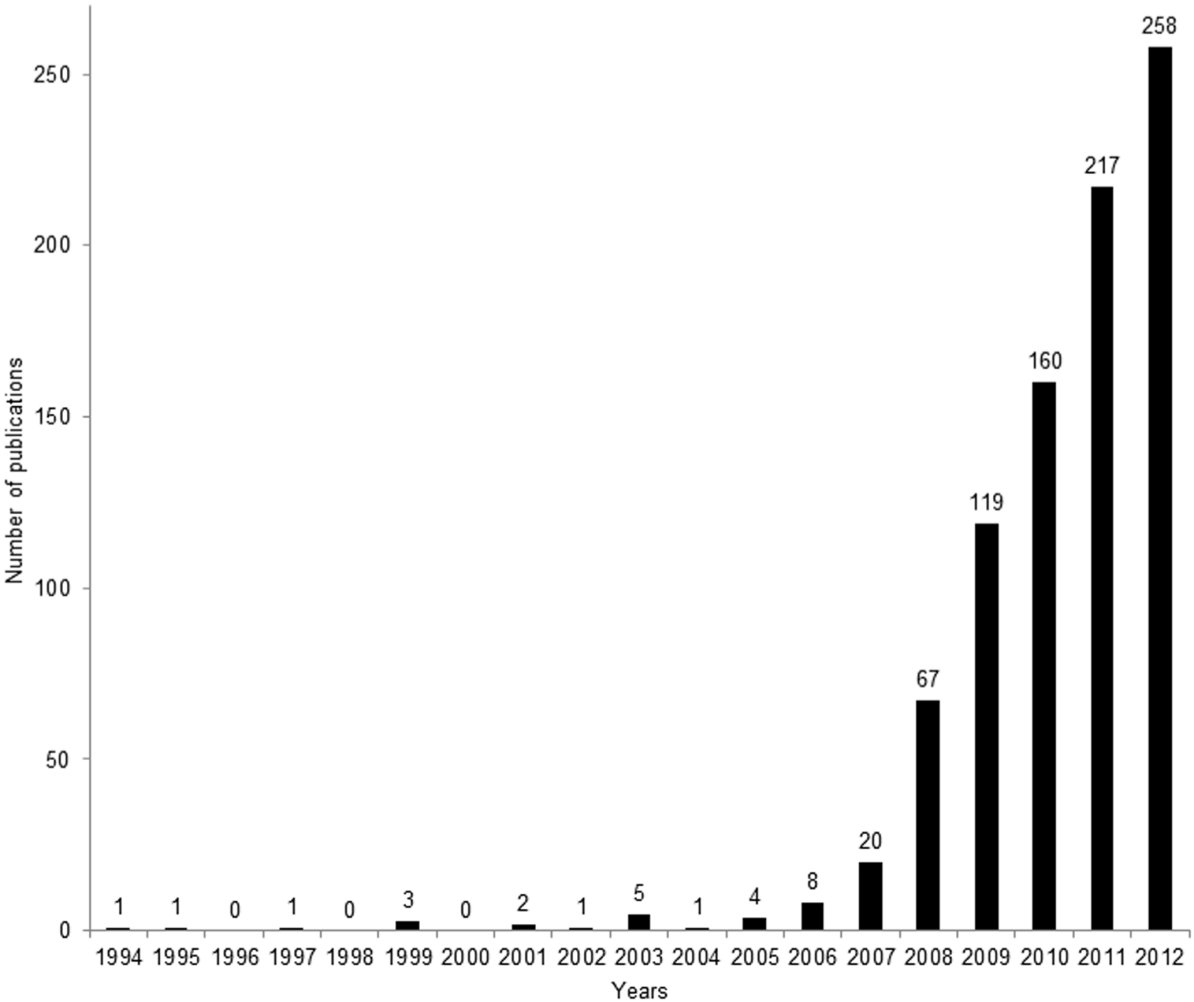
Number of publications mentioning a term for the measure. Number of publications per year (1994–2012) in which a term was mentioned for the measure entailing intentional human-mediated dispersal of organisms. The total number of publications mentioning a term was 868.

Taylor and Hamilton [6] were the first to mention a specific term for the approach, in 1994. In the years 1996, 1998, and 2000 we found no specific terms referring to the measure (Fig. 2). Otherwise, up till 2006, we found one to eight publications mentioning the approach with a specific term. In 2007, it was mentioned by a term in 20 publications, and subsequently in more publications each year until the score for 2012 was 275. This steep increase in interest (Fig. 2) was probably stimulated by a combination of alarming predictions of the impacts of climate change on biodiversity and articles in high-profile scientific journals discussing the option of alleviating the impacts by moving species.

**Figure 3:**
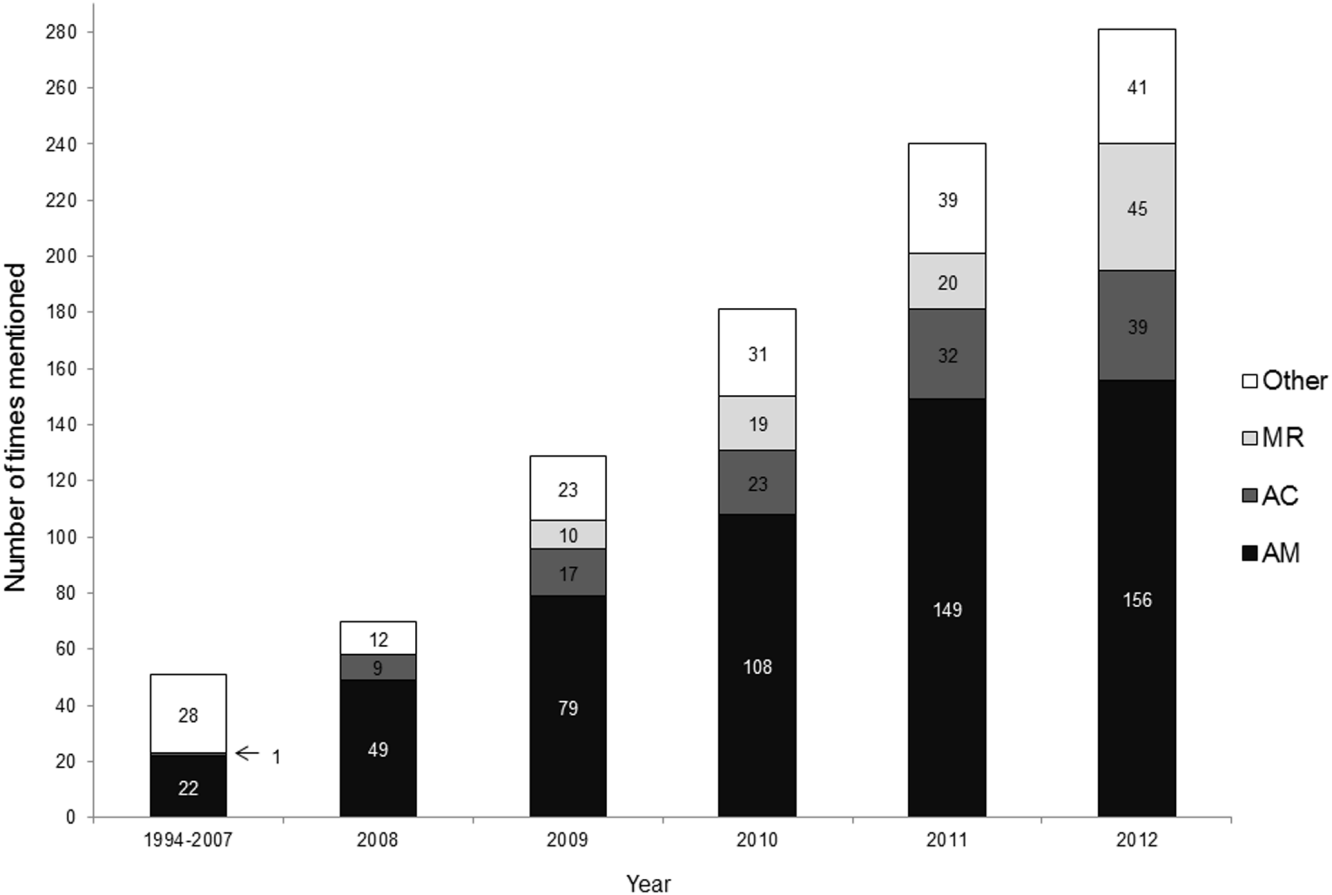
Number of times the three most common terms were used. Number of times the three most common terms denoting a conservation measure entailing intentional human-mediated dispersal of organisms in response to climate change were used as compared to other terms. AM  =  assisted migration; AC  =  assisted colonization; MR  =  managed relocation; Other  =  all other terms found in the literature search (N = 39; see Table 1).

We found 40 different terms for the idea (Table 1). The most commonly used terms were *assisted migration* (mentioned 563 times; first by Whitlock and Milspaugh in 2001 [7]), *assisted colonisation* (121; by Holmes et al. in 2007 [8]), and *managed relocation* (94; by Richardson et al. in 2009 [9]) (Fig. 3).

To promote unbiased, relevant, comprehensive, and exclusive discussions and studies, the term for the approach should neither be highly value-laden nor widely used in other contexts. For example, a highly positively value-laden term might support the idea of moving species in response to climate change prematurely, without solid scientific support for the action. It is also important that the term is descriptive of the approach. The modifying term should delineate the main term, and together they should describe the focal act and communicate the action in an unbiased and unambiguous way.

#### The main term

*Colonisation* (used by numerous authors) means establishing colonies. In this context it implies that what is helped in moving is also helped in establishing a viable population at the new site. While this may sometimes be needed for successful conservation, in many cases dispersing the organisms would be enough. A possible problem with *colonisation* is that it might bring in negative connotations from invasion biology.

*Dispersal* (e.g., [44]) is central in discussions concerning climate change impacts on biodiversity and encompasses the concrete action of the new approach. Failure to disperse is the reason for the suggested need to help species move to new areas. Thus, *dispersal* would be suitable for a term describing the idea discussed here.

*Introduction* (e.g., [26]) is defined by the IUCN ([45] and [43]) as “the intentional or accidental dispersal by human agency of a living organism outside its historically known native range”. As such, it is a much wider concept than moving organisms threatened by climate change for conservation purposes. Moreover, *introduction* may be associated with invasive alien species, which might hamper a neutral discussion. This is true also for *invasions process* [46]. *Reintroduction* [47], in a conservation context, is “movement and release of an organism inside its indigenous range from which it has disappeared” [43]. Thus, it does not communicate the idea of moving organisms to new areas.

*Migration* (numerous authors) has been criticised because, in zoology, it is associated with seasonal or diurnal movements back and forth and would therefore not clearly capture the aim of establishing new populations [31], [48], [49], [40]. However, one of the conventional meanings for the word *migration* is “extension of the distribution of a plant or animal” [35]. Hence, *migration* may be used in the term if associated with another descriptive word. *Migration management* [50] as a combined main term brings in nothing new, but puts the emphasis on *management*, making humans active managers of distribution areas. We argue that the emphasis should instead be on the actual process that is being helped.

*Range expansion* [51] is not suitable here because expansion implies becoming larger. In many cases *range shifting* is a more appropriate description of what takes place. Other combined main terms that we found include *species dispersal* [52], *species relocation* [53], and *ecosystem migration* [54]. Although a specification of what is being moved could be useful, it may be misleading to include only one or a few units in the term (see below for a review on the *what*-part of the definition).

*Relocation* (numerous authors) refers to displacing individuals, but species or whole populations will not usually be actively relocated. *Translocation*, *introduction*, *relocation*, and *reintroduction* also suffer from redundancies if combined with active adjectives such as *assisted*, *human-aided*, *planned*, or *managed* since they imply human activity in themselves.

*Restoration* [55] and *afforestation* [56] emphasise the receiving area, not the organisms that would be moved. While these terms are useful in the context of doing something to a degraded ecosystem, they are not descriptive of protecting threatened species by moving them.

According to the IUCN [47], [43] *translocation* [57] is an umbrella concept involving a variety of accidental or intentional “human-mediated movement[s] of living organisms from one area, with release in another” [43]. The IUCN [43] also defines a subcategory, *conservation translocation*, referring to translocating organisms specifically for conservation purposes. Thus, both *translocation* and *conservation translocation* are wider concepts that do not exclusively refer to the mitigation of a threat posed by climate. *Conservation translocation* includes situations where the organism is in danger due to other threat factors, such as land conversion. Thus, these terms are not restricted to approaches with a climate change dependent direction of the translocation.

*Dispersal*, *colonisation* and *migration* could be seen as a continuum ranging from singular *dispersal* events allowing the dispersed individuals to locally *colonise* a new site and finally resulting in *migration*, i.e., a change in distribution area. When helping organisms to move to new areas, it is essentially the *dispersal* event that is helped to enable local *colonisations* and, ultimately, *migration*. Colonisation could be helped as well, but that is not always necessary. In a directional, climate change motivated measure *dispersal* and, ultimately, *migration* is helped. As *migration* and *dispersal* thus both are descriptive words for the conservation approach, either could be a suitable main term to be used together with a modifying term.

#### The modifying term

*Artificial* (e.g., [58]) implies intentional human-made modifications and is related to such ambiguous terms as *unnatural*, *non-natural*, and *natural* [59]–[63]. In an environmental context, and especially when contrasted with *natural*, the term *artificial* may be value-laden, mostly negatively so, and may thus fail to fairly describe a conservation action.

*Assisted* (numerous authors) refers to helping and, hence, usually excludes accidental species introductions. *Assisted* therefore seems well suited for describing an intentional introduction. It can, however, be seen as positively value-laden to some degree. *Adaptation assisted* [64] would refer to the specific purpose of adaptation to, e.g., climatic change, and thus removes the emphasis from assisting migration. Likewise, combining *assisted* (or any other modifying term) with words such as *forestry*, *species rescue*, or *population* (as in *forestry assisted migration* or *assisted population migration*; [65]) may be useful in specific cases, but in a general term such detail is not needed.

*Benign* [66] implies kindliness and a favourable outcome, and as such is positively value-laden to the extent of compromising objectivity. *Conservation* [43] indicates the purpose of an action, but is inclusive of any introductions with a conservational aim including those to areas where the organism would not disperse on itself driven by climate change.

*Facilitated* (e.g., [67]) does not contain the idea of the discussed approach (cf. [5]–[6]), but could rather refer to the already established conservation action of facilitating species movements through the construction of dispersal corridors enabling spontaneous dispersal.

Most of the modifying terms contain words that *per se* communicate human involvement. Thus, in *human-aided* [68] and *human-assisted* (e.g., [69]), *human* is redundant. *Human-assisted migration management* [50] brings human involvement into the term multiple times as *management* and even *assisted* are likely associated with human action. It also seems that *human-assisted dispersal* is already used for describing accidental dispersal of species to new areas (e.g., [70]–[71]).

*Managed* (numerous authors) has the meaning of being subject to control, guidance, and influence. It can be seen as positively or negatively value-laden, depending on one's attitudes. *Managed* also has the flavour of succeeding and coping with. It communicates that the action is handled in a well-ordered way, which indeed should be the aim when using any conservation approach. However, management often refers to a concrete and continuous intervention. Such continuous activity may not always be included in a conservation approach involving moving organisms in response to climate change: just helping their dispersal may be sufficient.

*Planned* [46] is a dispositional term. Planned actions follow a pre-set design, which is well in agreement with the idea of moving species. However, *planned* does not communicate the actual action of translocating: the action could be just planned but never conducted. *Transformative* [72] refers to something being rather radically altered and is thus not descriptive of the approach, since most practitioners envision minimal change in the ecosystems receiving new organisms.

Summing up, we suggest that *assisted* is the best-suited word for the first part of the term. It may be slightly positively value-laden and we acknowledge that if the measure is found unsuitable, any promotion of it conveyed by a term is undesirable. Compared to other candidates, however, *assisted* suffers from fewer downsides.

#### The complex term

To denote a conservation approach entailing intentional and directional moving of species threatened by climate change, the above analysis identified *dispersal* or *migration* as a suitable main term and *assisted* as the most apt modifying term. We pointed out that the essence of this approach is dispersal but that its ultimate aim is migration. Thus we find that the complex terms *assisted dispersal* and *assisted migration* are both suitable and descriptive. However, because of its wide use (Figs. 1 and 2) we propose *assisted migration* as the term for the focal conservation approach, the exact definition of which is discussed below.

### Definition review

We found 84 definitions in 66 articles of the 130 peer-reviewed articles that mention a term for the approach in their title, abstract, or keywords (Fig. 1; Table S1). However, nine of the definitions (e.g. [73]–[74]) clearly had a different focus: they emphasised the management of the receiving *area* instead of the *unit* to be moved (species, population; Table S2). As this focus is fundamentally different from that of species-specific conservation, we included only the remaining 75 definitions from 60 publications in the analysis (Table S3).

**Figure 4:**
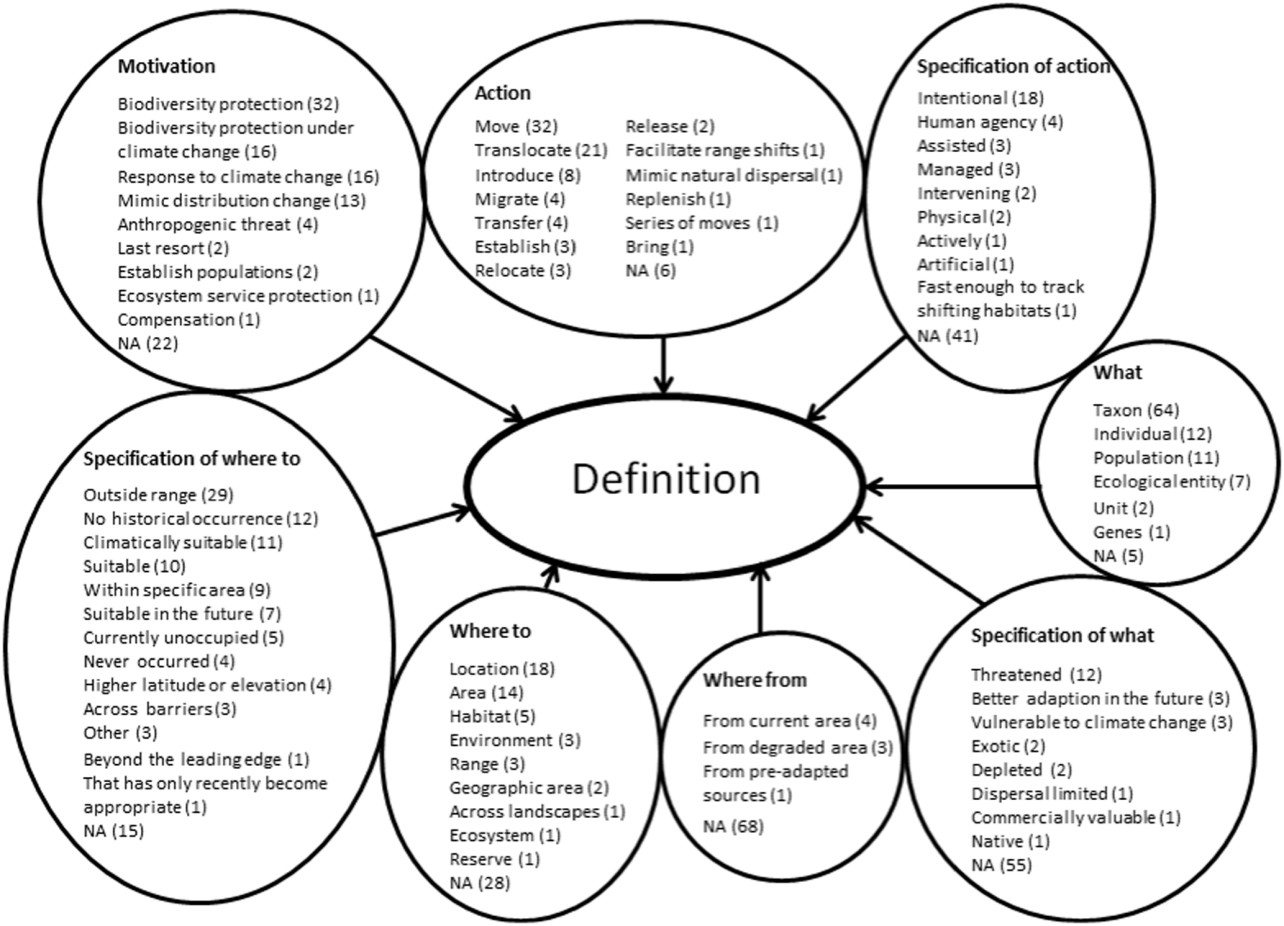
Main categories and groups formed from the definitions found in the literature review. The groups are divided into eight main categories (in bold; see text for further clarification). The numbers after each group refers to the total number of analysis units placed in that group. *NA* =  the definition did not contain a part referable to this main category; the number denotes how many definitions lacked this part.

In the content analysis, we found 485 analysis units, and classified them into 70 groups in eight main categories (see also Fig. 4 and Table S3):

1. *action* (what is done; 13 groups; 82 units);
2. *specification of action* (in what manner is something done; 9 groups; 35 units);
3. *what* (what is transferred; 6 groups; 97 units);
4. *specification of what* (8 groups; 25 units);
5. *where from* (the current location; 3 groups; 8 units);
6. *where to* (recipient area; 9 groups; 48 units);
7. *specification of where to* (13 groups; 99 units); and
8. *motivation* (9 groups; 87 units).

All definitions did not contain analysis units adhering to all of the main categories while others contained several units that were placed in the same group and/or main category. Some parts of the original definitions were split up in a way that reduced the detailed meaning of that part (see Table S3 for a description of how the definitions were divided into groups and main categories). For example, a part of a definition like “to a new area where more favourable conditions prevail” was split up in three analysis units and was placed in major categories as follows: *area* (where); *new* (specification of where) and *where more favourable conditions prevail* (specification of where).

A definition of a term should communicate an idea in a concise and understandable manner. Ideally, a definition expresses the necessary and sufficient conditions for something to belong to the scope of the defined term, i.e., it captures all actions that the term covers and leaves out everything else [75]. With this kind of definition, everyone using the term can discuss, study, and apply the same thing, and confusion can be minimised.

Below, we evaluate how the definition should be worded to best describe this conservation approach. We conclude by constructing a definition using the articulation and categories we see necessary and descriptive for representing the idea.

#### Action

We constructed 13 groups referring to the *action* (Fig. 4). The definitions most commonly included some form of the word *move* (N = 32), which is neutral and, when used as a transitive verb, highlights the active nature of the method. *Move* would thus be suitable as part of the definition.

Words belonging to the group *transfer*, including *transfer* and *transport*) may refer to moving individuals or populations in their entirety, leaving nothing at the starting point. The same applies for *translocate* and *relocate*. However, *translocation* is a general concept in conservation biology referring to moving organisms (e.g., IUCN [45], [43]; and see above). Although it could be argued to bring in unnecessary confusion with related terms, the measure in question is a sort of translocation and thus the verb *translocate* could be suitable for describing the action as part of the definition of the focal approach.

*Replenish* implies reintroduction to a former distribution area, which is not the case with this approach. To *introduce* has a negative association from invasion ecology and would therefore not necessarily be appropriate for the definition (see review above). Although most introduced species don't have a negative impact on their new habitat, *introduction* has been defamed by the invasive ones. *Bring* could be a suitable substitute, but if used as part of a definition, it might emphasise the receiving area or the arrival phase, not what is being moved nor the entire process.

*To establish* refers to seeing to that the moved individuals also succeed. However, concrete actions to ensure this are not necessarily included, as a mere *release* of individuals or propagules in the new area may be sufficient. *Release*, in turn, is too specific, and refers to mobile species that can actually be released (cf. *planted*).

A *series of moves* may describe the actual application of the approach in some cases, but is unnecessarily detailed and exclusive. Similarly, to *mimic natural dispersal* and *facilitate range shift* render unnecessary complexity and imprecision. *Migrate* is unsuitable for describing the *action* in a definition since it does not convey the human action in itself, but requires an active verb (such as *to assist* the migration).

In summary, *move* and *translocate* are the most applicable words to describe the action. We propose to use *translocate*, because it is defined and established in the field of conservation (e.g., [45] and [43]). A new approach defined using this word would relate it to established translocation methodologies carried out for conservational purposes, albeit for other reasons than climate change.

#### Specification of action

Several definitions described the human involvement in the approach with words such as *assisted*, *human-aided*, or *mediated*. These we grouped together as referring to the idea of *assisted*. We discussed *assisted* under *Terminological review* and find it descriptive also for the definition.

The approach was also described with wordings such as *by human agency*, *human intervention* or *proactive*. These refer to initiating change and a sense of human stewardship over nature, and may not clearly enough convey the adaptive rather than transformative notion of the approach.

*Physical* is not very informative since any moving of organisms can hardly be anything else. *Actively* is not exclusive, as also unintentional moving can be active. *Artificial* is reviewed above, and for the reasons described we do not recommend it as part of the definition. *Fast enough to track shifting habitats* emphasises the temporal dimension of the action and although it may be discussed as a detail in the implementation of the method, such level of detail seems unnecessary in a general definition.

The action was sometimes described using *purposeful* or *intentional*. This notion is apt in that it conveys the idea that something is done on purpose as opposed to accidentally and would rule out unintentional spreading of species.

Purposeful, assisted or intentional could all be useful for specifying the action. However, their suitability depends on the word describing the *action*. As we have found *translocation* to be the best word for describing it (see previous section), a specification by *purposeful*, *assisted*, or *intentional* would bring in tautology, as translocation is already established as intentional moving of species by human agency. Thus, we do not think this part is needed in the definition.

#### What

Most definitions in this category referred to words that we grouped into *taxon* where the most common word was *species* (others were: *taxa*, *plants*, *animals*, *subspecies* and *ecotype*; see Fig. 4). Other groups we formed were *population*, *individual*, *ecological entity*, *genes*, and *units*.

As what is being moved is an essential part of the definition it is worthwhile considering this aspect a bit further. The threat of climate change may not appear the same for all populations within a species, since there may be both genetic and climatic differences between regions within the range of a species. Thus, this approach may be applied to only certain populations of a species, and the definition should also embrace cases where specific populations are moved *outside the population's current range*, but *within the species' current range*. Also, from a philosophical point of view, it is inappropriate to speak about moving *species*, since a species is an abstract construct used to describe patterns of recurrence in the living world or to refer to temporally delimited entities that operate within a continuous evolutionary process (e.g., [76]–[77]). Moreover, a species is intangible also in practice: an entire species cannot really be moved, or at least this cannot be verified. Instead, the levels at which this methodology would operate will more likely be the *individual* or *organism*, or a part of a *population*. *Genes* could be an inclusive way to describe what is being moved since they are always moved when any biological unit is moved. However, this would make the definition quite abstract and could also provoke connotations to genetic modification of organisms.

Some authors defined the unit to be moved as an *ecological entity* (*functional form*, *life form*, *flora*, *fauna, ecosystem*). Even though moving certain parts of ecosystems may sometimes be the case, using these in the definition would divert the focus from species conservation toward conservation of ecosystem functions, which we feel should be kept separate to avoid confusion. Furthermore, the operationalization will have to happen at the level of individual organisms or their propagules, instead of broad, abstract groups.

In the group *unit*, we placed *biological units* and *focal units*, both of which allow the focus to lie case-specifically on species, populations, groups of species etc. They also allow the new area to be specified in relation to the current distribution of that particular unit (instead of, e.g., by the distribution of the species it belongs to). However, they may be vague for non-biologists.

On the basis of the aspects above, none of the descriptions seem quite suitable. Therefore, we propose to combine the best sides of the above suggestions and describe what is being translocated with *representatives of a species or population*. This wording allows the definition to include any suitable propagule and does not exclude smaller units than species.

#### Specification of what

In some definitions the kinds of units in focus were specified (Fig. 4). Most of them we grouped under *threatened* (e.g., *threatened*, *endangered* and *high priority*), while those that mentioned a characteristic of the unit as a reason for the threat were placed into *vulnerable to climate change* or *dispersal-limited*. *Exotic* is negatively value-laden due to terms such as *exotic species*, and *native* is not relevant here. *Depleted* is vague and would be more suitable if talking about reintroductions. *Commercially valuable*, in turn, underlines the economic value of the species over conservational ones.

Words such as *threatened* and *endangered* can be part of the definition but *high priority* is exclusive. However, since the translocation of species can be motivated by several reasons, including habitat destruction and over-exploitation, it is important to emphasise climate change as the main threat for the species. This is important because the recipient area is differently defined in these differing instances (see discussion under *specification of where to*). However, as *threatened* is a well-recognised category of the Red List, a rigid use of a definition containing it could result in using assisted migration only for red-listed species. In practise, the measure should be applicable also for species or populations that are not classified as threatened, but are adversely affected by climate change. In order to make a distinction between this climate change motivated directional approach and other, non-directional translocations, we suggest defining the species or population as *harmed by climate change*.

#### Where from

Only eight definitions mentioned from where the species should be moved, resulting in three groups: from current area (in situ, existing natural habitats, or current area of occupancy), from degraded area (habitat predicted to become unsuitable, hostile environments, or degrading ecosystems), and from pre-adapted sources. As species may be moved from different sources – e.g., directly from their natural populations or from ex situ collections when the in situ populations are too scarce to allow sufficient harvesting – the inclusion of where from in a definition is not necessary and would be too limiting.

#### Where to

Of the nine groups in this category, we think that *habitat*, *range*, *environment*, and *across landscapes* are too wide, while referring to a certain *geographic area* (e.g., *high latitude* or *mountain*), *ecosystem*, or *reserve* is not general enough. *Area* can be interpreted as a wide entity (c.f. *distribution area*) while *location* refers to a more limited spatial entity, an exact place. Therefore we suggest using *area*. However, to properly indicate where something should be moved, most of the definitions included a *specification of where to*.

#### Specification of where to

This specification is a key part of the definition, since it demarcates the main difference between this and other conservation translocations. Several definitions mentioned translocating the species to where they do not exist (Fig 4). We grouped these analysis units according to their exclusiveness, from translocating to *other* areas, which does not specify the focal area at all, through *outside range* and *currently unoccupied*, further to *no historic occurrence*, and finally to the most restrictive *never occurred*. However, these are all too broad to single out translocations motivated by climate change, as they do not specify the direction of the movement and can thus imply introduction to any area outside the range of the species. Furthermore, several aspects make the use of historical species distributions as reference points ambiguous ([40]; and response [78]). Most importantly, there is no widely accepted definition of historical distribution. Various stakeholders may, hence, interpret it differently according to their needs, which in turn may lead to substantial confusion between disciplines and in practical applications. A further complication is that historical distribution is usually related to the concept of species, leading to problems when the operational unit of assisted migration is something else than a species, e.g., a locally adapted population. For example, Liu et al. [79] tested the difference in success of individuals moved outside the species' historical range vs. those moved within the range of the species. We would argue that for a specific population, which may be locally adapted, human-constructed boundaries between species and their ranges are irrelevant, and thus, operationalizing the idea in this manner leads to obscure experiments and conclusion drawing.

Many definitions specified the new area as being *suitable* (here including *favourable*) without any further specification, while others referred to areas *suitable in the future* or to an area *that has only recently become appropriate* with no reason specified. As we are trying to find a definition that could single out assisted migration from other conservation translocations, the climatic aspect is an essential criterion. Just describing the new area as *suitable* is ambiguous, since it implies any suitable area and contains neither a reason for the suitability nor a direction of the movement.

Other attributes specifying where the species should be moved were: across barriers, beyond the leading edge, to higher latitude or elevation, and within specific area that included, within natural range, to adjacent area, to contiguous environment, and to parts of the same biogeographic area. These are unnecessarily specific or too ambiguous for a general definition of the topic. For instance, using relative terms such as far outside and just outside [37] may be treacherous, as the definitions then also remain relative, hampering unambiguous operationalization and understanding.

A number of definitions specified the new place as *climatically suitable* (*climatically buffered* or *where climate is projected to become suitable*), which sets the precondition that the suitability of the target site should be evaluated.

We suggest that when defining a conservation approach that aims at protecting a species or population harmed by climate change, the best description for the target area could be *outside its indigenous range where it would be predicted to move in response to climate change*. This formulation allows the definition to include the movement of organisms within the distribution area of a larger entity (e.g., populations vs. species), since *outside of its indigenous range* is related to the focal population. The description contains a precondition (*predicted to move in response to climate change*) whereby it excludes relocating a species or a population due to, e.g., land-use. It also includes the precondition of estimating where the suitable area will lie as climate changes. These estimations can be based on projections ranging from expert views to sophisticated models. In practice also other factors determining site suitability must be considered when choosing the specific sites within the climatically suitable area. However, site suitability is not a necessary part of a definition of the approach, but something requiring consideration during the planning of an actual assisted migration project.

It is important also to identify why the species or population would not reach the new area and why assisted migration is needed to ensure its continued survival. For this, a further condition is needed: where it would be predicted to move in response to climate change, *were it not for anthropogenic dispersal barriers or lack of time*. This specifies the spatial and temporal reality for why the focal species needs help: rapid climate change limits the time it has to disperse over natural barriers, and anthropogenic barriers are further dispersal obstacles.

#### Motivation

The underlying reason for the approach was part of almost all published definitions. The most common incentive was *biodiversity protection* (e.g. *conservation* and *reducing extinction risks*) followed by climate change, either as a *response to climate change* or more specifically *biodiversity protection under climate change*.

Also other motives were mentioned in the definitions, such as *anthropogenic threat*, *ecosystem service protection* or to *establish populations* in new areas. These motives imply different kinds of translocations for, e.g., conservational, cultural, or economic purposes and are not exclusive enough for this approach. Other anthropogenic threats, such as land-use, deforestation, over-harvesting, or pollution may all be good reasons for applying conservation translocations, but do not imply a movement following the direction of climate change.

To *mimic distribution change* was also suggested for explaining that the distribution area is changing. We think this verbalisation could be used if combined with climate change. However, it is not needed if the definition contains a description of *where* the biological unit is moved, which mentions climate change.

We also found some definitions containing the idea of the proposed measure as a *last resort* or as *compensation*. Although this may sometimes be the reality, it is unnecessary to delimit the application of the approach in this way, as for some species it may be the best alternative. Neither do we know whether it will be seen as a compensation for the harm done by humans to biodiversity or as way to sustain biodiversity for its instrumental value.

The motivation for the action narrows down the scope of application and thereby diminishes confusion. Therefore it is one of the necessary conditions that the definition should include. We suggest defining the motive of the measure through mentioning *safeguarding of biological diversity* in the definition. However, it is also crucial to mention climate change in order to separate the focal approach from other translocations. The motivation will become clear in the definition through *harmed by climate change* (specification of what), and *where it would be predicted to move in response to climate change* (specification of where).

#### Defining assisted migration

Based on the above analysis, we propose the definition “Assisted migration means safeguarding biological diversity through the translocation of representatives of a species or population harmed by climate change to an area outside the indigenous range of that unit where it would be predicted to move as climate changes, were it not for anthropogenic dispersal barriers or lack of time”.

This definition follows the original idea of Peters and Darling [5], but elucidates it by bringing in the necessary conditions needed to separate it from other approaches that focus on moving organisms. Thus, only the actions that meet the definition should be regarded as assisted migration. The definition is both exclusive and inclusive. It includes translocations of threatened populations within a species' range, but it excludes translocations made to enhance economic activities (e.g., forestry) by restricting this measure to safeguarding biodiversity. It also excludes conservation translocations motivated by other threats than climate change, or targeted to areas outside the predicted climate change driven dispersal. It is important to be able to separate the motivation behind the focal action, as the motivation may be decisive as regards investment, prioritisation, and stakeholders both in research and society at large.

### Assisted migration and related ideas

**Figure 5:**
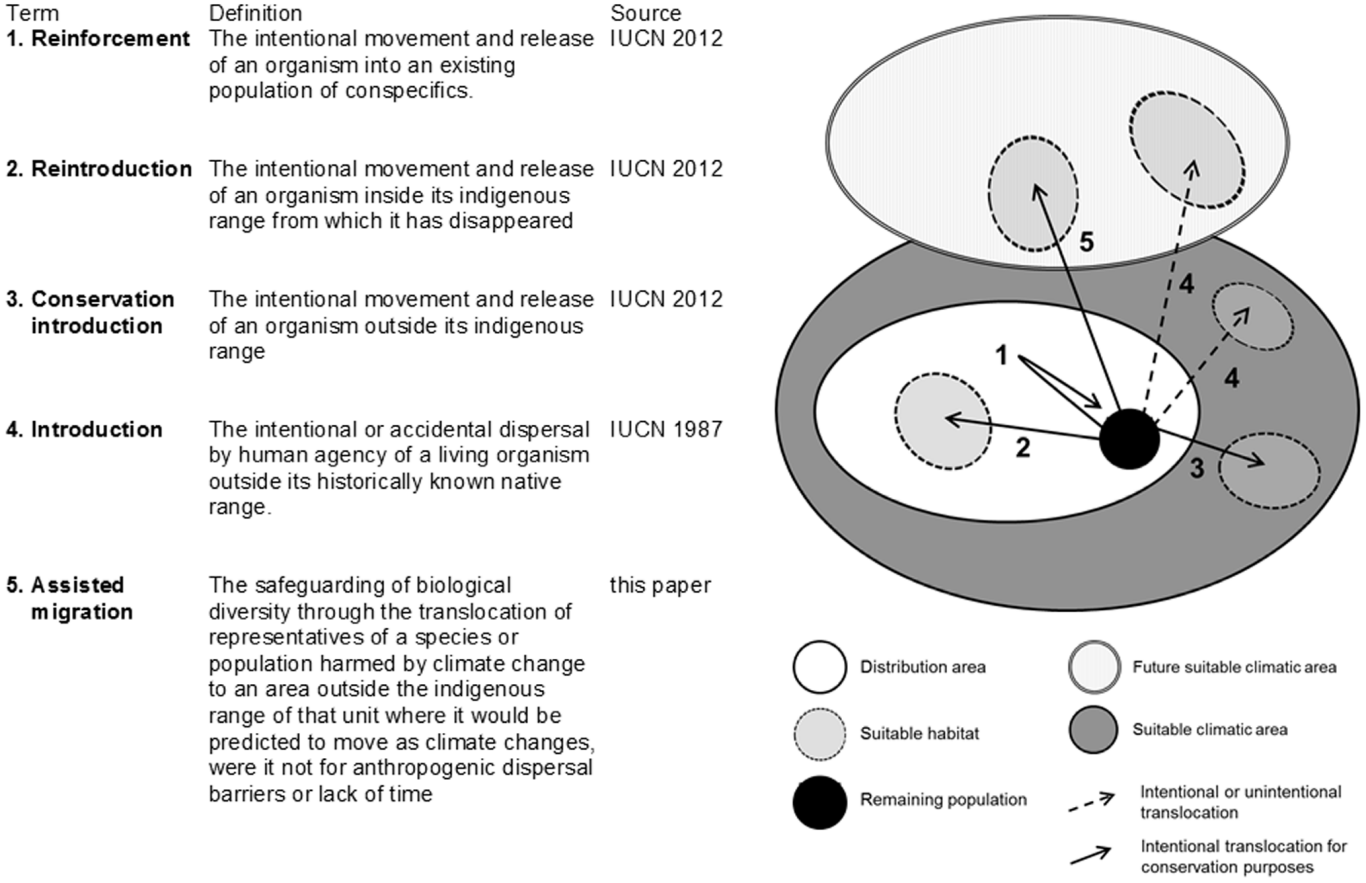
Definitions and a model of translocation concepts. According to the IUCN [45], *translocation* is defined as the movement of living organisms from one area with free release in another.

In Figure 5 we present the definitions for other translocation concepts and their relationships to each other and to assisted migration. *Translocation* (as defined by IUCN [45] and [43]) is an umbrella concept for several kinds of translocations, including assisted migration. In their guidelines for conservation translocations, the IUCN [43] define *conservation introduction* as “the intentional movement and release of an organism outside its indigenous range” and subdivides this into two types: *assisted colonisation* and *ecological replacement*. *Conservation introduction* [66] and *assisted colonisation* [43] are defined as measures that could be used when no other options exist and the species cannot be re-enforced or translocated within its current range. However, they are not specifically related to climate change.

We argue that it is necessary to specify a type of translocation where dispersal is assisted because of a change in climate and, thus, in the suitable distribution area. We have not been able to identify any other force than climate change that would make a species' or population's current distribution area unfavourable while simultaneously making another area favourable. Anthropogenic climate change thus requires a re-evaluation of the way we conserve biodiversity. For this, we need clear concepts. Any other translocation outside a species current range for conservation purposes should be called something else than *assisted migratio*n, which could then be reserved for translocations that are directional as a response to climate change. Hence, *assisted migration* should not be seen as synonymous with *assisted colonisation* but as a subcategory of it.

We noticed a discrepancy in the definition of the measure between the fields of conservation and forestry. Most of the forestry-related definitions emphasised a silvicultural viewpoint, which is not included in the original idea of assisted migration, which has to do with safeguarding biodiversity. Pedlar et al. [65] place forestry in the assisted migration debate by introducing the concepts *forestry assisted migration* and *species rescue assisted migration*. This is a movement in the right direction, since it distinguishes these two concepts, which are fundamentally different in their goal. Nevertheless, to avoid confusion, we suggest that other terms should be used for strategies seemingly similar to assisted migration but applied for purposes other than safeguarding biodiversity. Choosing suitable provenances in the context of agri-, silvi- or horticulture in relation to anticipated changes in climate could be called, e.g., *predictive provenancing* [80] to avoid confusion with *assisted migration*.

## Conclusions

We have quantified the discussion on moving species as a response to climate change by reviewing the proposed terms and definitions for the general idea. Based on that, we propose a term and definition specifically for aiding species threatened by climate change to shift their ranges. Assisted migration is a kind of conservation translocation ([43]; Fig. 5), which can be distinguished from other conservation translocations by the following three aspects:

1. It is directional and based on a prediction of the potential future distribution of the biological unit;
2. It is limited to translocations as a way to overcome temporal or spatial dispersal limitations; and
3. It is used to mitigate threat caused directly or indirectly by anthropogenic climate change.

In these respects this measure is not only separable from other conservation translocations, but also clearly different from many kinds of species introductions discussed in the literature, where representatives of species are introduced far away from their original distribution areas, even to other continents where they would not disperse on their own. Such discussions include the invasive alien species problem (e.g., [81]), Colombian exchange [82], Pleistocene rewilding [83], and the interpretation of including the moving of polar bears from the Arctic to the Antarctic within the scope of assisted migration (cf. [84]).

A clear articulation of an idea enables better scientific operationalization. If the concept can be defined, hypotheses can be generated and tested, and the applicability of the method can be critically evaluated and, if deemed feasible, developed. Without conceptual clarity, there is a high risk for doing confused science that does not actually test what was intended. The concept can be supported by a well-constructed definition and a suitable term for the idea. All this is highly relevant in the development or restriction of a conservation strategy and methodology where scientific theory, ethical considerations, legislation, and application need to be inter-related and fluent to communicate without losing focus.

## Supporting Information

Table S1 **The 868 publications found through the literature search that mention a term for moving species in connection to climate change.**
(PDF)

Table S2 **Definitions not included in the concept analysis since they focus on the receiving area rather than the unit to be moved.**
(PDF)

Table S3 **Definitions and the analysis units derived from them.**
(PDF)

Checklist S1 (PDF)
