# Supplementary material for: Coming to Terms with the Concept of Moving Species Threatened by Climate Change – A Systematic Review of the Terminology and Definitions
Source: PLoS One. 2014 Jul 23;9(7):e102979. doi: 10.1371/journal.pone.0102979 (PMC4108403; doi:10.1371/journal.pone.0102979)
Supplement: Table S1 — The 868 publications found through the literature search that mention a term for moving species in connection to climate change. (PDF) [file pone.0102979.s001.pdf]

Supporting Information 1

Table S1. The 868 publications found through the literature search that mention a term for moving species in connection to climate change. Publication type abbreviations: A= Peer-reviewed article; B= Book; P= Popular or professional article including published abstracts of congress presentations; T= Thesis; and R= Report. Peer-reviewed arcticles that mention a term in the title, abstract, or key words and were searched for a definition are marked in bold.

Author	Title	Publication venue	Year	Used term	Publication type
A de Sherbinin et al.	Migration and risk: net migration in marginal ecosystems and hazardous areas	Environmental Research Letters	2012	Assisted Relocation	A
Ackerly et al.	The geography of climate change	Diversity and Distributions	2010	Managed Translocation	A
Adams-Hosking	Modelling climate-change-induced shifts in the distribution of the koala	Wildlife research	2011	Assisted Migration, Assisted Colonization	P
Adams-Hosking et al.	Modelling changes in the distribution of the critical food resources of a specialist folivore in response to climate change	Diversity and Distributions	2012	Assisted Colonization	A
Adrien et al.	A Distributed Graduate Seminar to Analyze the Priorities, Obstacles, and Opportunities that Exist for the Implementation of State Wildlife Action Plans	Available at: http://people.icess.ucsb.edu/research/documents/WildlifeActionPlansReport.pdf	2008	Assisted Migration	R
Aitken et al.	Adaptation, migration or extirpation: climate change outcomes for tree populations	Evolutionary applications	2008	Facilitated Migration	A
Aitken et al.	Adaptation of forest trees to climate change	Available at: http://www.mrnf.gouv.qc.ca/publications/forets/connaissances/recherche/Corporatif/Larix-Proceeding.pdf#page=23	2007	Facilitated Migration	P
Akcakaya	Conservation Biology	Encyclopedia of theorethical ecology	2012	Human Assisted Migration, Human Assisted Dispersal	B
Albrecht and Machinski	Influence of Founder Population Size, Propagule Stages, and Life History on the Survival of Reintroduced Plant Populations	Plant reintroduction in a changing climate	2012	Managed Relocation	B
Albrecht et al.	The Ethics of Assisted Colonization in the Age of Anthropogenic Climate Change	Journal of Agricultural and Environmental Ethics	2012	Assisted Colonization	A
Alfaro	Forest health in a changing environment	Mery et al. (eds.): Forests and Society - Responding to Global Drivers of Change	2010	Assisted Migration	B
Ali and Trivedi	Botanic gardens and climate change: a review of scientific activities at the Royal Botanic Gardens, Kew	Biodiversity and Conservation	2011	Assisted Migration	A
Al-Khafaji	Climate Change Portfolio Review - Literature Review and Evaluation Matrix	Available at: http://dev.cepf.net/Documents/CEPF_ClimateChangeReview_lit_August2008.pdf	2008	Assisted Migration	R
Allendorf et al.	Genomics and the future of conservation genetics	Nature	2010	Assisted Migration	A

Andalo and Bousquet	The impact of climate change on growth of local white spruce populations in Québec, Canada	Forest Ecology and Management	2005	Translocation	A
Andel et al.	Unifying concepts	Restoration Ecology: The new frontier	2012	Assisted Migration, Managed Relocation	B
Anderson et al.	Evolutionary and Ecological Responses to Anthropogenic Climate Change	Plant Physiology	2012	Assisted Migration	A
**Anderson et al.**	**Immigrants and refugees: the importance of dispersal in mediating biotic attrition under climate change**	**Global Change Biology**	**2012**	**Assisted Migration**	**A**
Anderson et al.	Evolutionary genetics of plant adaptation	Science	2011	Assisted Migration	A
Anonymous	An Assessment of the Vulnerability of Forest Vegetation of Ontario’s Clay Belt (Ecodistrict 3E-1) to Climate Change	Available at: http://www.cabi.org/cabdirect/FullTextPDF/2013/20133008758.pdf	2012	Assisted Migration	R
Anonymous	Climate Change Impacts and Adaptation Options for the Island Forests of Saskatchewan	Available at: http://www.parc.ca/rac/fileManagement/upload/12855_3C12%20_IslandForests_Report_2012.pdf	2012	Assisted Migration	R
Anonymous	Climate Change: A scientific Assessment for the GEF	Available at: http://www.thegef.org/gef/sites/thegef.org/files/publication/Climate%20Change-A%20Scientific%20Assessment%20for%20the%20GEF_2.pdf	2012	Assisted Migration	R
Anonymous	GSDUpdate august 2012	Available at: http://www.fs.fed.us/rm/grassland-shrubland-desert/docs/gsd-update/2012-08.pdf	2012	Assisted Migration	P
Anonymous	US Forest Service Climate Change Adaptation Priorities Team Charter	Available at: http://www.fs.fed.us/sustainableoperations/documents/ClimateChangeAdaptationPrioritiesTeamCharter.pdf	2009	Assisted Migration	R
Anonymous	Assisted colonisation: Flitter further north.	Nature	2009	Assisted Colonisation	P
Anonymous	Avoiding the heffalump trap	The Economist	2009	Assisted Migration	P
Anonymous	California wildfires put focus on prevention methods.	Fire engineering	2009	Assisted Migration	P
Anonymous	Guidelines for Using the IUCN Red List	Available at: https://78462f86-a-3cc5a544-s-sites.googlegroups.com/a/lrfv.org/www/docs/RedListGuidelines_7.0_2008.pdf?attachauth=ANoY7cqhZ2508DS1OwuuWXjXbhJ4rdK0vx7f4NR30yNEMUlfA54INNXqizawbSRV3THAONtpGQYFpaaG8rYCO4lI2kDY218UxncLUF4mw5CuTJTOjnVv4l9vXNYCyLurHsBr6x5ewJSwxeJLSSfFnippZQ_lAMno3bqY7RbddHCYkG0COCASKL16VQ5tED5u96nQm33zS-zxFo4Xd3n9p5mjPszZVpoOSuTsGsC4W2AxYbmVSnfdgbs%3D&attredirects=0	2008	Benign Introduction	R
Anonymous	Potential Ecological Consequences of Climate Change in South Florida and the Everglades: 2008 Literature Synthesis	Available at: http://erec.ifas.ufl.edu/research/pdf/wildlife/TRClimateChangeLoResSecure.pdf	2009	Assisted Colonization	R

Anonymous	Preparing the Pacific Northwest for Climate Change A Framework for Integrative Preparation Planning for Natural, Human, Built and Economic Systems	Available at: http://scholarsbank.uoregon.edu/jspui/bitstream/1794/6989/1/Preparing_PacNW_for_ClimateChange_4-2-08.pdf	2008	Assisted Migration	R
Anonymous	Implications of Climate Change in British Columbia's Southern Interior Forests	Available at: http://cmiae.org/_PDF/ImpofCCinforestsfinal.pdf#page=108	2005	Assisted Migration	R
Anonymous	Climate Change Adaptation – Marine Biodiversity and Fisheries	Marine Adaption Bulletin	2011	Managed Translocation	R
Anonymous	IUCN guidelines for re-introductions	Available at: http://intranet.iucn.org/webfiles/doc/SSC/SSCwebsite/Policy_statements/Reintroduction_guidelines.pdf	1995	Benign Introduction, Conservation Introduction	B
Aplet and Gallo	Applying Climate Adaptation Concepts to the Landscape Scale	Available at: http://ecoadapt.org/data/documents/Aplet_Gallo_2012_Climate_Adaptation_Portfolio_Sierra_Stanislaus-Low-Res_Version.pdf	2012	Assisted Migration, Managed Relocation	R
Appell	Escape from the killing field	Scientific American Magazine	2009	Assisted Migration	P
Arana et al.	High genetic variation in marginal fragmented populations at extreme climatic conditions of the Patagonian Cypress Austrocedrus	Molecular Phylogenetics and Evolution	2010	Assisted Migration	A
Araujo	Protected Areas and Cliamte Change in Europe.	Available at: http://www.azoresbioportal.angra.uac.pt/files/noticias_Inf10e_2009_Protected_Areas_and_Clim_Change_ARAUJO_Sept09-1.pdf	2009	Assisted Migration	R
Arribas et al.	Evaluating drivers of vulnerability to climate change: a guide for insect conservation strategies	Global Change Biology	2012	Assisted Colonization	A
Aschwanden	GSD Update: Restoration on the range: Healing America's iconic landscapes	GSDUpdate	2011	Assisted Migration	P
Ash	Adaption to Climate change: a merging challenge	ABC broadcasting	2010	Assisted Migration	P
Aubin et al.	Why we disagree about assisted migration1: Ethical implications of a key debate regarding the future of Canada's forests	Forestry Chronicle	2011	Assisted Migration	P
Ault et al.	Northern Hemisphere Modes of Variability and the Timing of Spring in Western North America	Journal of Climate	2011	Assisted Migration	A
Austin et al.	Natural Selection and Phenotypic Plasticity in Wildlife Adaptation to CC	Wildlife conservation in a changing climate	2012	Assisted Migration	B
Baer	Engaged Anthropology in 2011: A View from the Antipodes in a Turbulent Era	American anthropologist	2012	Assisted Migration	P
Bagne and Finch	Vulnerability of Species to Climate Change in the Southwest: Threatened, Endangered, and At-Risk Species at the Barry M. Goldwater Range, Arizona	Available at: http://www.fs.fed.us/rm/pubs/rmrs_gtr284.pdf	2012	Assisted Migration	R
Barbosa et al.	Positive regional species–people correlations: a sampling artefact or a key issue for sustainable development?	Animal Conservation	2010	Assisted Migration	A

Barbour and Kueppers	Conservation and Management of Ecological Systems in a Changing California	Available at: https://www.ppic.org/content/pubs/report/R_1108EBR.pdf	2008	Assisted Migration	R
Barbour and Kueppers	Conservation and management of ecological systems in a changing California	Climatic Change	2012	Managed Relocation	A
Barlow	Deep time lags: Lessons from pleistocene ecology	Available at: http://www.google.com/books?hl=fi&lr=&id=N9TlKJEuuawC&oi=fnd&pg=PA165&dq=%22assisted+migration%22+OR+%22assisted+coloni*ation%22+OR+%22managed+relocation%22+OR+%22human-aided+translocation%22+OR+%22assisted+translocation%22+AND+%22climate+change%22&ots=AW13oiy5V5&sig=V7Klg-DkQ0pljtPkIPwidy_7UkM#v=onepage&q=%22assisted%20migration%22%20OR%20%22assisted%20coloni*ation%22%20OR%20%22managed%20relocation%22%20OR%20%22human-aided%20translocation%22%20OR%20%22assisted%20translocation%22%20AND%20%22climate%20change%22&f=false	2010	Assisted Migration	B
**Barnes**	**Tree Response to Ecosystem Change at the Landscape Level in Eastern North America**	**Forestarchiv**	**2009**	**Assisted Migration**	**P**
Barnett	Exit Obstructed	Nature Reports Climate Change	2009	Assisted Migration	P
Barney and DiTomaso	Bioclimatic predictions of habitat suitability for the biofuel switchgrass in North America under current and future climate scenarios	Biomass and Bioenergy	2009	Benign Introduction	A
**Barnosky**	**Transforming conservation**	**Issues in Science & Technology**	**2010**	**Managed Relocation**	**A**
Barton et al.	Risky movement increases the rate of range expansion	Proceedings of the Royal Society B: Biological Sciences	2012	Assisted Colonization	A
Basse et al.	The 7 Aarhus Statements on Climate Change	IOP Conf. Series: Earth and Environmental Science	2009	Assisted Migration	R
Battaglia	A conceptual framework for restoration and conservation of coastal ecosystems subject to chronic sea-level rise	Available at: http://eco.confex.com/eco/2008/techprogram/P9532.HTM	2008	Assisted Migration	P
Bayard	Testing the Role of Social Cues in Saltmarsh Sparrow Habitat Selection Decisions	Available at: http://hydrodictyon.eeb.uconn.edu/people/birdlab/Bayard_T._dissertation_final%20draft.pdf	2010	Assisted Colonization	T
Beardmore and Winder	Review of science-based assessments of species vulnerability: Contributions to decision-making for assisted migration	Forestry Chronicle	2011	Assisted Migration	P
Beever	Testing alternative models of climate-mediated extirpations	Ecological applications	2010	Assisted reintroduction	A
Beever and Belant	Ecological consequences of Climate Change: Synthesis and research needs	Beever: Ecological Consequences of Climate Change: Mechanisms, Conservation, and	2011	Assisted Migration	B

Beierkuhnlein et al.	Ecotypes of European grass species respond differently to warming and extreme drought	Journal of Ecology	2011	Assisted Migration, Assisted Colonization	A
Beir	Conceptualizing and Designing Corridors for Climate Change	Ecological restoration	2012	Assisted Colonization	A
Bellard et al.	Impacts of climate change on the future of biodiversity	Ecology Letters	2012	Human Assisted Colonization	A
Bennett	Multi-scale comparison of native and exotic communities in the Garry oak ecosystem of British Columbia	Available at: https://circle.ubc.ca/handle/2429/41207	2012	Assisted Migration	T
Bennett	Return of the Fauna: Brown treecreeper reintroduction in eucalypt woodland	Australian National Univeristy	2012	Assisted Colonization	T
Bennett	Climate change and the implications for forest fires in British Columbia	Available at: https://circle.ubc.ca/handle/2429/36455	2011	Assisted Migration, Assisted Ecosystem Migration	T
Benson	Intelligent Tinkering: the Endangered Species Act and Resilience	Law and Social-Ecological Resilience	2012	Assisted Migration	A
Bergstrom et al.	The Northern Rocky Mountain Gray Wolf Is Not Yet Recovered	Bioscience	2009	Human Assisted Migration Management	P
Berlin et al.	Impact of Sea Level Rise on Plant Species.	Available at: http://trapdoor.bren.ucsb.edu/research/2012Group_Projects/documents/sealevelrise_report_000.pdf	2012	Assisted Migration	R
**Bernazzani et al.**	**Integrating Climate Change into Habitat Conservation Plans Under the U.S. Endangered Species Act**	**Environmental Management**	**2012**	**Assisted Migration**	**A**
Bert Rambunctious Garden	Saving Nature in a Post-Wild World	Civil Engineering	2012	Assisted Migration	A
Betancourt	Reflections on the relevance of history in a nonstationary world	Historical Environmental variation in conservation and natural resource…	2012	Assisted Migration	B
Bevaqua et al.	Multi-objective assessment of conservation measures	International Council for the Exploration of the Sea	2007	Assisted Migration	B
Bhattarai	Phenotypic and Genetic Characterization of Wildland Collections of Western and Searls Prairie Clovers for Rangeland Revegetation in the Western USA	Available at: http://digitalcommons.usu.edu/cgi/viewcontent.cgi?article=1814&context=etd	2010	Assisted Migration	T
Bicca-Marques and Calegaro-Marques	Are the Risks of Conservation Introduction Worth Taking?	Zoologica (Curitiba)	2012	Conservation Introduction	A
Biggs et al.	Toward Principles for Enhancing the Resilience of Ecosystem Services	Annual Review of Environment and Resources	2012	Assisted Migration	A
Bingham	The Role of Ectomycorrhizal Networks in Plant-to-Plant Facilitation Across Climatic Moisture Gradients.	Available at: https://circle.ubc.ca/bitstream/handle/2429/30532/ubc_2011_spring_bingham_marcus.pdf?sequence=3	2010	Assisted Migration	T
Birks and Willis	Alpines, trees, and refugia in Europe	Plant Ecology and Diversity	2008	Assisted Migration, Assisted Colonization	A
Black-Samuelson	The state of forest genetic	Available at: http://shop.skogsstyrelsen.se/shop/9098/art74/14648674-c3c5ce-1851.pdf	2012	Assisted Dispersal, Adaption Assisted Migration	R

Blois Once and Future Giants	What Ice Age Ex- tinctions Tell Us about the Fate of Earth's Largest Animals.	The Quarterly Review of Biology	2012	Managed Relocation	A
Bodinoff et al.	Habitat attributes associated with short-term settlement of Ozark hellbender (Cryptobranchus alleganiensis bishopi) salamanders following translocation to the wild	Freshwater Biology	2012	Managed Relocation	A
Bombi et al.	Will climate change affect terrapin (Pelusios subniger paritalis and P. castanoides intergularis) conservation in Seychelles?	Phelsuma	2009	Assisted Migration	P
Bonte et al.	Costs of dispersal	Biological reviews	2012	Assisted Migration, Assisted Dispersal	A
Booth	Biosecurity: when one size does not fit all	Feral Herald - Newsletter of the Invasive species Council, Australia	2011	Translocation	P
Bork	Listed Species Reintroductions on Private Land - Limiting Landowner Liability	Stanford Environmental Law Journal	2011	Assisted Migration	P
Bower and Aitken	Ecological genetics and seed transfer guidelines for Pinus albicaulis (Pinaceae)	American Journal of Botany	2008	Facilitated Migration	A
Bower et al.	Effects of length of storage, and stratification on germination of whitebark pine seeds	Weasterm journal of applied forestry	2011	Assisted Migration	A
**Bradley and Wilcove**	**When Invasive Plants Disappear: Transformative Restoration Possibilities in the Western United States Resulting from Climate Change**	**Restoration Ecology**	**2009**	**Transformative Restoration**	**A**
Bradley et al.	Predicting plant invasions in an era of global change	Trends in Ecology & Evolution	2010	Assisted Migration	A
Bragg et al.	Restoring Old-Growth Southern Pine Ecosystems	Available at: http://www.arlis.org/docs/vol1/213500339/pnw_gtr_733_7.pdf	2007	Facilitated Migration	P
Breed et al.	Clarifying climate change adaptation responses for scattered trees in modified landscapes	Journal of Applied Ecology	2011	Assisted colonisation	A
Bremont and Engle	Adaptation Theme Report: Terrestrial Ecosystem Resilience	Available at: http://www.geog.umd.edu/sites/geog.umd.edu/files/Terrestrial_Ecosystems_Theme_Final_Report.pdf	2011	Facilitated Migration, Assisted Colonization	R
Broadmedow et al.	Observed Impacts of Climate Change	Available at: http://www.tsoshop.co.uk/gempdf/ClimateChange_Main_Report_Section_2_Impacts.pdf	2009	Facilitated Migration	R
**Brooker et al**	**Literature review: species translocations**	**Scottish Natural Heritage Commissioned Report No. 440**	**2011**	**Assisted Colonisation**	**R**
Broome et al.	A brief review of the life history of, and threats to, Burramys parvus with a pre-history based proposal for ensuring that it has a future	Wildlife and Climate Change: Towards robust conservation strategies for Australian fauna	2012	Assisted Translocation	B
Brown	Sustainable adaption: an oxymoron	Climate and development	2011	Assisted Migration	P
Bullock	Plant dispersal and the velocity of climate change	Dispersal Ecology and Evolution	2012	Assisted Migration	B
Bunnell and Kremsater	Actions to promote climate resilience in forests of british columbia	Journal of Environmental Monitoring	2012	Assisted Migration	P

**Burbridge**	**Is Australia ready for Assisted Colonization? Policy changes required to facilitate translocations under climate change**	**Pacific Conservation Biology**	**2011**	**Assisted Colonization**	**A**
Burgiel and Muir	Invasive Species, Climate Change and Ecosystem-Based Adaptation: Addressing Multiple Drivers of Global Change	IUCN report: Available at: http://data.iucn.org/dbtw-wpd/edocs/2010-054.pdf	2010	Assisted Migration	R
Burke et al.	A mobility index for Canadian butterfly species based on naturalists' knowledge	Biodiversity and Conservation	2011	Assisted Colonization	A
Burton et al.	Sustainability of boreal forests and forestry in a changing environment.	Available at: http://orton.catie.ac.cr/repdoc/A5253I/A5253I.PDF	2010	Facilitated Migration	R
Bustamanete et al.	Queensland's biodiversity under climate change: coastal and marine ecosystems	Climate Adaptation Flagship Working Paper #12E	2012	Assisted Migration	R
Butler	The Destination of Species	Mother Jones	2012	Assisted Migration	P
Byers and Norris	Climate Change Vulnerability Assessment of Species of Concern in West Virginia	Available at: http://129.71.224.73/publications/PDFFiles/ClimateChangeVulnerability.pdf	2011	Assisted Migration	R
Byrne	Management intervention needed to enhance biological diversity	Available at: http://www.ncseonline.org/sites/default/files/Management%20Interventions%20(09%2030%2011B)%20final.pdf	2011	Assisted Migration	R
Callahan	Continental-scale characterization of molecular variation in quaking aspen	Available at: http://digitalcommons.usu.edu/etd/1297/	2012	Assisted Migration	T
Camacho	Transforming the Means and Ends of Natural Resources Management	The North Carolina law review	2011	Assisted Migration	A
**Camacho**	**Assisted Migration: Redefining Nature and Natural Resource Law Under Climate Change**	**Yale Journal on Regulation, Vol. 27, p. 171, 2010**	**2010**	**Assisted Migration**	**A**
Camacho	Adapting Governance to Climate change: Managing uncertainty through learning infrastructure	Emory Law Journal	2009	Assisted Migration	A
Camacho	The legality and ethics of assisted migration	Available at: http://eco.confex.com/eco/2009/techprogram/P15588.HTM	2009	Assisted Migration	P
Campbell et al.	The linkages between biodiversity and climate change adaptation	UNEP World Conservation Monitoring Center	2009	Assisted Migration, Human Assisted Translocation, Assisted Colonization, Human Assisted Dispersal, Human Aided Translocation	R
Campos-Arceiz and Blake	Megagardeners of the forest – the role of elephants in seed dispersal	Acta Oecologica	2011	Assisted Dispersal	A
Carlson et al.	Maintaining the role of Canada’s forests and peatlands in climate regulation	The Forestry Chronicle	2010	Assisted Migration	P
Carlton	The global dispersal of marine and estuarine crustaceans	In the Wrong Place - Alien Marine Crustaceans: Distribution, Biology and Impacts	2011	Assisted Migration	B

Carmona-Catot et al.	Long-term captive breeding does not necessarily prevent reestablishment: lessons learned from Eagle Lake rainbow trout	Reviews in Fish Biology and Fisheries	2012	Assisted Migration	A
Caro et al.	Conservation in the Anthropocene	Conservation Biology	2012	Assisted Migration	A
Carrete and Tella	Is assisted colonization feasible? Lessons from past introductions	The Ecological Society of America	2012	Assisted Colonization	A
**Carroll et al.**	**Climate change and translocations: The potential to re-establish two regionally extinct butterfly species in Britain**	**Biological Conservation**	**2009**	**Assisted Colonisation**	**A**
Carter and Blair	High richness and dense seeding enhance grassland restoration establishment but have little effect on drought response	Ecological applications	2012	Managed Relocation	A
**Carter and Blair**	**Seed source affects establishment and survival**	**Ecosphere**	**2012**	**Managed Relocation**	**A**
Carvalho et al.	From climate change predictions to actions–conserving vulnerable animal groups in hotspots at a regional scale	Global Change Biology	2010	Assisted Dispersal, Assisted Colonization	A
Casaril et al.	Hydro-climate Knowledge	Available at: https://publications.csiro.au/rpr/download?pid=csiro:EP122609&dsid=DS8	2012	Assisted Migration	R
Casper et al.	Review of the Blue Groper	Available at: http://www.imas.utas.edu.au/__data/assets/pdf_file/0006/197205/Nov-4-SE-CC-Meeting-report_FINAL.pdf#page=37	2011	Managed Translocation	R
CBD	SBSTTA: Biological Diversity and Climate Change.	Available at: http://69.90.183.227/doc/meetings/sbstta/sbstta-09/information/sbstta-09-inf-12-en.pdf#page=44	2003	Human Assisted Migration	R
Ceriani et al.	The survival strategy of the alpine endemic Primula glaucescens is fundamentally unchanged throughout its climate envelope despite superficial phenotypic variability	Plant Ecology	2009	Assisted Migration	A
Chandra	Opportunities for ecological adaptation in Tasmania	Available at: http://alvinchandra.yolasite.com/resources/Documents/Opportunities%20for%20Ecological%20Adaptation%20in%20Tasmania_Final%20AC230810.pdf	2010	Assisted Migration	R
Chandrapavan	Translocation of the southern rock lobster, Jasus edwardsii, to improve yield and marketability	Available at: http://eprints.utas.edu.au/10613/	2010	Managed Relocation	T
Chapin	Managing Ecosystem Sustainability: The key role of resilience	Principles of ecosystem stewardship	2009	Assisted Migration	B
Chapin et al	Temporal Dynamics	Principles of terrestrial ecosystem ecology	2011	Assisted Migration	B
Chapin et al.	Temporal dynamics	Principles of Terrestrial Ecosystem Ecology	2012	Assisted Migration	B
Chapin et al.	Ecosystem stewardship: sustainability	Trends in Ecology and Evolution	2010	Assisted Migration	A

Chapin et al.	Resilience-based Stewardship: Strategies for Navigating Sustainable Pathways in a Changing World	Chapin et al.: Principles of Ecosystem Stewardship	2009	Assisted Migration	B
**Chapin et al.**	**Managing Climate Change Impacts to Enhance the Resilience and Sustainability of Fennoscandian Forests**	**AMBIO: A Journal of the Human Environment**	**2007**	**Assisted Relocation**	**A**
Chapin et al.	Earth Stewardship: science for action to sustain the human-earth system	Ecosphere	2011	Assisted Migration	P
**Chapron and Samelius**	**Where species go, legal protections must follow**	**Science**	**2008**	**Assisted Colonization**	**A**
**Chauvenet et al.**	**Maximizing the success of assisted colonizations**	**Animal Conservation**	**2012**	**Assisted Colonization**	**A**
**Chen et al.**	**Tropical botanical gardens: at the in situ ecosystem management frontier**	**Trends in plant science**	**2009**	**Assisted Migration**	**A**
Chester	The Conservation and Climate Change Clearinghouse: An online informational hub for biodiversity conservationists	Available at: http://eco.confex.com/eco/2010/techprogram/P25857.HTM	2010	Managed Relocation	P
Chester et al.	Climate change science, impacts and opprtunities	Climate and conservation	2012	Translocation	B
**Chmura et al.**	**Forest responses to climate change in the northwestern United States: Ecophysiological foundations for adaptive management**	**Forest Ecology and Management**	**2011**	**Assisted Migration**	**A**
Clarke	Conserving Biodiversity in the Face of Climate Change	Agenda	2007	Assisted Migration	P
Clements et al.	Wildfire Rehabilitation and Restoration: Triage in the Pursuit of Resilience	Rangelands	2009	Assisted Migration	P
Cliquet	Is Environmental Law Ready for New Ecological Restoration Concepts?	Available at: http://digitalcommons.law.umaryland.edu/gelc/2012/july2_3C/4/	2012	Translocation, Managed Relocation of Species, Trans Situ Conservation	C
Coenen et al.	Future directions in conservation and development: Incorporating the reality of climate change	Biodiversity	2008	Assisted Migration, Assisted Colonization	A
Cole	Planned Diversity - The case for a system with Several Types of Wilderness	International Journal of Wilderness	2011	Assisted Migration	P
Cole	Wilderness Restoration: From philosophical questions about naturalness to tests of practical techniques	Available at: http://www.fs.fed.us/rm/pubs_other/rmrs_2008_cole_d002.pdf?	2008	Assisted Migration	P
**Cole et al.**	**Past and ongoing shifts in Joshua tree distribution support future modeled range contraction**	**Ecological applications**	**2011**	**Assisted Migration, Assisted Relocation**	**A**
Cole et al.	Responding to Climate Change: A Toolbox of managements strategies	Beyond Naturalness: Rethinking Park and Wilderness Stewardship in an Era of Rapid Change	2010	Assisted Migration	B
Cole et al.	Naturalness and Beyond: Protected Area Stewardship in an Era of Global Environmental Change	Available at: http://www.lter.uaf.edu/dev2009/pdf/1244_Cole_Yung_2008.pdf	2008	Assisted Migration	B

**Coles and Riegl**	**Thermal tolerances of reef corals in the Gulf: A review of the potential for increasing coral survival and adaptation to climate change through assisted translocation**	**Marine Pollution Bulletin**	**2012**	**Assisted Translocation, Assisted Migration**	**A**
Coll et al.	Biodiversity and Climate Change in Ireland	Available at: https://www.foe.ie/download/pdf/biodiversity_and_climate_change_in_ireland_briefing_paper.pdf	2008	Assisted Migration	R
Collevatti et al.	Range shift and loss of genetic diversity under climate change in Caryocar brasiliense, a Neotropical tree species	Tree Genetics & Genomes	2011	Assisted Migration	A
Collin	Conserving intraspecific biodiversity of forest trees in France and Europe	Available at: http://www.set-revue.fr/sites/default/files/archives/article_06.pdf	2011	Assisted Migration	R
Collins	Amphibian decline and extinction	Diseases of aquatic organisms	2010	Managed Relocation	A
**Collyer et al.**	**Contemporary Evolutionary Divergence for a Protected Species following Assisted Colonization**	**PLOS ONE**	**2011**	**Assisted Colonization**	**A**
Colombo et al.	Managing tree seed in an uncertain climate: conference summary	Climate Change Research Information Note	2008	Assisted Migration	P
Colwell et al.	Coextinction and Persistence of Dependent Species in a Changing World	Annual Review of Ecology, Evolution, and Systematics	2012	Assisted Migration	A
Comer et al.	Climate change vulneability and adpatation strategies for natural communities	Available at: https://connect.natureserve.org/sites/default/files/documents/NatureServe_HCCVI_Report.pdf	2012	Assisted Migration	R
Considine	Moving on: Relocating species in response to climate change	ECOS Magazine	2011	Managed Relocation	P
Cook-Patton	Reframing conservation goals to meet 21st century challenges	Available at: http://www.esajournals.org/doi/full/10.1890/0012-9623-93.3.253	2012	Assisted Migration	C
Corlett	The shifted baseline: Prehistoric defaunation in the tropics and its consequences for biodiversity conservation	Biological conservation	2012	Assisted Migration, Assisted Dispersal	A
Corlett	Impacts of warming on tropical lowland rainforests	Trends in Ecology and Evolution	2011	Assisted Migration	A
**Cortini et al.**	**Climate effects on red alder growth in the Pacific Northwest of North America**	**Forest Ecology and Management**	**2012**	**Assisted Migration**	**A**
Craig	"Stationarity is dead" - long live transformation: five principles for climate change adaption law	Harvard Environmental Law Review	2010	Assisted Migration	A
Craig	Stationarity is Dead - Long Live Transformation: Five Principles for Climate Change Adaptation Law	Harvard Environmental Law Review	2010	Assisted Migration	P
Crispo et al.	Broken barriers: Human-induced changes to gene flow and introgression in animals	BioEssays	2011	Assisted migartion, Assisted Colonisation, Managed Relocation	A
Crowe and Parker	Conserving the diversity of Ontario tree species	Canadian Journal of Forest Research	2011	Facilitated Migration	P

**Crumpacker**	**Implications of Climatic Warming for Conservation of Native Trees and Shrubs in Florida**	**Conservation Biology**	**2001**	**Human Assisted Migration**	**A**
Curtis-McLane and Aitken	Can whitebark pine grow north of its current species range under climate change? A genetic analysis using common gardens	Available at: http://eco.confex.com/eco/2009/techprogram/P18024.HTM	2009	Facilitated Migration	P
Cushman et al.	Climate Change and Connectivity: Assessing Landscape and Species Vulnerability	Available at: http://cel.dbs.umt.edu/documents/GPLCC_Draft_Final_for_Broska_Review.pdf	2010	Assisted Migration	R
Dalacho	Plant Species and Functional Diversity along Altitudinal Gradients, Southwest Ethiopian Highlands	Available at: http://opus.ub.uni-bayreuth.de/volltexte/2010/649/pdf/Diss.pdf	2009	Assisted Migration	T
Dalrymple et al	A Meta-Analysis of Threatened Plant	Plant reintroduction in a changing climate	2012	Managed Relocation	B
Damschen	Resampling Robert Whittaker's Siskiyou plots and using traits and ordination to interpret change	Available at: http://eco.confex.com/eco/2010/techprogram/P25361.HTM	2010	Managed Relocation	P
**Damschen et al.**	**Endemic plant communities on special soils: early victims or hardy survivors of climate change?**	**Journal of Ecology**	**2012**	**Managed Relocation**	**A**
Damschen et al.	Climate Change and Plant Communities on Unusual Soils	Harrison & Rajakaruna: Serpentine: The Evolution and Ecology of a Model System	2011	Assisted Migration	B
Damschen et al.	Climate change effects on an endemic-rich edaphic flora	Ecology	2010	Managed Relocation	A
Daust and Morgan	Management strategies for climate--change	Available at: http://www.bvcentre.ca/files/research_reports/ManagementStrategies-ClimateChangeNadina-Nov15.pdf	2011	Facilitated Migration	R
**Davidson and Simkanin**	**Skeptical of Assisted Migration**	**Science**	**2008**	**Assisted Colonization**	**A**
Davies	Long-term approaches to native woodland restoration: Palaeoecological and stakeholder perspectives on Atlantic forests of Northern Europe	Forest Ecology and Management	2011	Translocation, Assisted Migration	A
Davis	Testing the Accuracy of Ecological Niche Models Using the Last Glacial Maximum Fossil Record of Mammals.	Available at: https://gsa.confex.com/gsa/2012AM/finalprogram/abstract_211735.htm	2012	Assisted Migration	C
Davis et al.	The Impact of Climate Change on Indigenous Arabica Coffee (Coffea arabica): Predicting Future Trends and Identifying Priorities	PLOS ONE	2012	Assisted Migration	A
Dawson et al	Climate change and tree genetic resource management	Agroforest syst	2011	Facilitated Translocation	A
Dawson et al.	Beyond Predictions: Biodiversity Conservation in a Changing Climate	Science	2011	Assisted Migration, Human Assisted Migration	A
Dean	The Preservation Predicament	New York Times	2008	Assisted Migration	P
Deane-Drummond and Clough	Introduction: Genetics, ecology, and conservation	Reviews in Religion and Theology	2009	Assisted Migration	B

Debinski and Cross	Conservation and Global	Conservation Biology Available at: http://press.princeton.edu/blog/wp-content/uploads/2010/10/Debinski-V-6_Conservation-and-Global-Climate-Change.pdf	2008	Assisted Migration	B
Decamps	River networks as biodiversity hotlines	Comptes Rendus Biologies	2011	Managed Relocation	A
del Castillo	Genetic factors associated with population size may increase extinction risks and decrease colonization potential in a keystone tropical pine	Evolutionary Applications	2011	Assisted Migration	A
**del Castillo et al.**	**Pinus chiapensis, a keystone species**	**Forest ecology and management**	**2009**	**Assisted Migration**	**A**
Delach and Matson	Climate Change and Federal Land Management	Available at: http://www.defendersofwildlife.com/resources/publications/programs_and_policy/gw/climate_change_and_federal_land_management.pdf	2010	Managed Relocation	R
D'Elia	Evolution of Avian Conservation Breeding With Insights	Journal of Fish and Wildlife Management	2010	Assisted Migration, Assisted Colonization	A
Dickens et al.	Stress: An inevitable component of animal translocation	Biological Conservation	2010	Assisted Migration, Assisted Colonization	A
**Dietl and Flessa**	**Conservation paleobiology using the past to manage the future**	**Paleontological Society Papers**	**2009**	**Assisted Migration**	**A**
Dixon and Hopper	An introduction to Caladenia R.Br. – Australasia's jewel	Australian Journal of Botany	2009	Assisted Migration	A
Dixon and Tremblay	Biology and natural history of Caladenia	Australian Journal of Botany	2009	Assisted Migration	A
Dobson et al.	Corridors: Reconnecting fragmented habitats	Soulé et al.: Continental Conservation: Scientific Foundations Of Regional Reserve Networks	1999	Artificial Translocation	B
Doledec and Statzner	Responses of freshwater biota to human disturbances: contribution	Journal of the North American Benthological Society	2010	Facilitated Dispersal	A
Donaldson	Botanic gardens science for conservation and global change	Trends in plant science	2009	Assisted Migration	A
Doremus	Adapting to Climate Change Through Law that Bends Without Breaking.	Available at SSRN: Available at: http://ssrn.com/abstract=1628255	2010	Managed Relocation	A
Douhovnikoff and Dodd	Lineage divergence in Coast Redwood , detected by a new set of nuclear microsatellite loci	American Midland Naturalist	2011	Assisted Migration	P
Drayton and Primarck	Success Rates for Reintroductions of Eight Perennial Plant Species after 15 Years	Restoration Ecology	2012	Assisted Migration	A
Dreiss	Differential Canopy Leaf Flushing and Site Nitrogen Status Facilitate Invasive Species Establishment in Temperate Deciduous Forest Understorie	Available at: http://digitalcommons.uconn.edu/gs_theses/173/	2011	Assisted Migration	T
Driscoll et al.	Priorities in policy and management when existing biodiversity stressors interact with climate-change	Climatic Change	2012	Assisted Migration	A
Dudgeon	Asian river fishes in the Anthropocene: threats and conservation challenges in an era of rapid environmental change	Journal of Fish Biology	2011	Assisted Migration	A

Early	Species extinction in an era of climate change: Climate-paths, dispersal barriers and the level of need for managed relocation	Available at: http://eco.confex.com/eco/2009/techprogram/P16252.HTM	2009	Managed Relocation	P
**Early and Sax**	**Analysis of climate paths reveals potential limitations on species range shifts**	**Ecology Letters**	**2011**	**Managed Relocation**	**A**
Eddington et al.	Monitoring Forest and rangeland species and ecological processes to anticipate and respond to cliMate change in British coluMBia	Available at: http://bcwildfire.ca/ftp/HFP/external/!publish/FREP/reports/FREP_Report_20.pdf	2009	Assisted Migration	R
Ellen and Platten	The social life of seeds: the role of networks of relationships in the dispersal and cultural selection of plant germplasm	Journal of the Royal Anthropological Institute	2011	Assisted Migration, Managed Migration, Assisted Relocation, Managed Relocation	A
Ellis	Anthropogenic transformation of the terrestrial biosphere	Philosophical transactions of the Royal Society	2011	Facilitated Migration	A
Enquist et al.	Jemez Mountains Climate Change Adaptation Workshop	Available at: http://www.climas.arizona.edu/files/climas/pubs/jemez-workshop-report-2009-final.pdf	2009	Assisted Migration	R
Eskelin et al.	Assessing assisted migration as a climate change adaptation strategy for Ontario's forests	Available at: http://www.cabi.org/cabdirect/FullTextPDF/2011/20113188037.pdf	2011	Assisted Migration	R
ESSA Technologies Ltd.	Vegetation Models and Climate Change	ESSA Technologies Ltd.: Vegetation Models and	2008	Assisted Migration	R
Esteve-Selma et al.	Potential effects of climatic change on the distribution of Tetraclinis articulata, an endemic tree from arid Mediterranean ecosystems	Climatic Change	2012	Artificial Translocation	A
Etterson	Evaluating the genetic consequences of assisted migration	Available at: http://eco.confex.com/eco/2009/techprogram/P15580.HTM	2009	Assisted Migration	P
**Fazey and Fischer**	**Assisted colonization is a techno-fix**	**Trends in Ecology and Evolution**	**2009**	**Assisted Colonization**	**A**
Feder et al.	Locomotion in response to shifting climate zones: not so fast	Annual Review of Physiology	2010	Managed Relocation	A
Fehr	It's saplings vs. climate change, 'Survivor' style	Canadian Geographic	2012	Assisted Migration	P
Fei et al.	Modelling chestnut biogeography for American chestnut restoration	Diversity and Distributions	2012	Managed reintroduction, Managed Relocation	A
**Fernadez-Manjarrez and Tschanz**	**Assisted colonization: protect managed forests.**	**Science**	**2010**	**Assisted Colonization**	**A**
Finch (ed)	Climate Change in Grasslands, Shrublands, and Deserts of the Interior American West	Available at: http://www.tribesandclimatechange.org/docs/tribes_535.pdf#page=54	2012	Assisted Migration, Assisted Colonization	R
**Fischman and Hyman**	**The legal challenge of protecting animal migrations as phenomena of abudance**	**Virginia Environmental Law Journal**	**2010**	**Translocation**	**A**
Fleishman and Nally	Measuring the response of animals to contemporary drivers of fragmentation	Canadian Journal of Zoology	2007	Assisted Migration, Human Assisted Migration	A
Fleishman et al.	Introduction to the Special Section on Alternative Futures for Great Basin Ecosystems	Restoration Ecology	2009	Transformative Restoration	A

Flikkema et al.	Towards Cyber-Eco Systems: Networked Sensing, Inference and Control for Distributed Ecological Experiments	Available at: http://ieeexplore.ieee.org/xpls/abs_all.jsp?arnumber=6468339&tag=1	2012	Assisted Migration	C
Flint et al.	Hope for the Forests? Habitat Resiliency Illustrated in the Face of Climate Change Using Fine-Scale Modeling	American Geophysical Union, Fall Meeting 2010, abstract #GC51I-0834: Available at: http://adsabs.harvard.edu/abs/2010AGUFMGC51I0834F	2010	Assisted Migration	P
**Fordham et al.**	**Managed relocation as an adaptation strategy for mitigating climate change threats to the persistence of an endangered lizard**	**Global Change Biology**	**2012**	**Managed Relocation**	**A**
Fox	Book review: Biodiversity for nonscientist	Ecology	2011	Assisted Migration	P
Frascaria-Lacoste	Local versus non local: managing in the face of uncertainty	Proceedings 7th European Conference on Ecological Restoration	2010	Planned Invasions Process	P
**Frascaria-Lacoste and Fernandes-Manjarres**	**Assisted Colonization of Foundation Species: Lack of Consideration of the Extended Phenotype Concept—Response to Kreyling et al. (2011)**	**Restoration Ecology**	**2012**	**Assisted Colonization**	**A**
Fraser	Climate Change Impacts in Biological Systems	Hooper (ed.): Proceedings of the Species at Risk 2004 Pathways to Recovery Conference	2004	Translocate	R
**Freilich and Reich**	**Wilderness conservation in an era of global warming and invasive species: a case study from Minnesota's Boundary Waters Canoe Area Wilderness**	**Natural Areas journal**	**2009**	**Assisted Migration**	**A**
Friggens et al.	Decision Support: Vulnerability, Conservation, and Restoration	Available at: http://www.buffelgrass.org/sites/default/files/FS-Grassland-Report-rmrs_gtr285.pdf#page=122	2012	Assisted Colonization	R
Fruchter	Do Large, Infrequent Disturbances Release Estuarine Wetlands fron Coastal Squeezing? D	Available at: http://opensiuc.lib.siu.edu/theses/923/	2012	Assisted Migration	T
Funk et al.	Harnessing genomics for delineating conservation units	Trends in Ecology and Evolution	2012	Assisted Migration	A
Gabol and Ahmed	Identification of future environmental challenges in Pakistan by 2025 through environment foresight	African Journal of Environmental Science and Technology	2011	Assisted Migration	A
Gaiser et al.	The Florida Everglades	Wetland Habitats of North America	2012	Human Assisted Migration	B
**Galatowitsch et al.**	**Regional climate change adaptation strategies for biodiversity conservation in a midcontinental region of North America**	**Biological Conservation**	**2009**	**Assisted Colonization, Assisted Migration**	**A**
Game et al.	Pelagic protected areas: the missing dimension in ocean conservation	Trends in Ecology & Evolution	2009	Assisted Migration	A
Garbelotto and Pautasso	Impacts of exotic forest pathogens on Mediterranean ecosystems: four case studies	European Journal of Plant Pathology	2012	Assisted Migration	A
Garzon et al.	Intra-specific variability and plasticity influence potential tree species distributions under climate change	Global Ecology and Biogeography	2011	Assisted Migration	A

Gayton	Impacts of Climate Change on British Columbia's Biodiversity	Available at: http://www.forrex.org/publications/forrexseries/fs23.pdf	2008	Assisted Migration	R
Geils	Western Forest Diseases and Climate	Available at: http://www.fs.fed.us/rm/pubs_other/rmrs_2008_geils_b001.pdf	2008	Assisted Migration	R
Gibbon et al.	Ecosystem Carbon Storage Across the Grassland–Forest Transition in the High Andes of Manu National Park, Peru	Ecosystems	2010	Assisted Migration, Assisted Afforestration	A
Gibbons	Going Back to the Future To Understand Climate Change	Science	2010	Assisted Migration	P
Gillison et al.	Holocene palaeo-invasions: the link between pattern, process and scale in invasion ecology?	Landscape Ecology	2008	Assisted Migration	A
Gillogly	Book reviews: Gaia in Turmoil	Human ecology	2011	Assisted Migration	P
Gitlin et al.	Genetic Diversity of Cottonwood (Populus sp.) Before, During, and After Establishmnet of Tamarix.	Arizona Riparian Council	2009	Assisted Migration	R
Glick et al.	A new era for conservation: Review of climate change adaption literature	Available at: http://sharepoint.nwf.org/sites/oc/statewildlife/publications/A%20New%20Era%20for%20Conservation.pdf	2009	Assisted Colonization	R
Glicksman	Climate change adaptation on federal lands	Environmentaland energy law pespectives	2012	Assisted Migration	P
Glicksman	Governance of Public Lands, Public Agencies, and Natural Resources	GWU Legal Studies Research Paper	2012	Assisted Migration	A
Glicksman	Ecosystem Resilience to Disruptions Linked to Global Climate Change: An Adaptive Approach to Federal Land Management	Nebraska Law Review	2009	Assisted Migration	A
Glicksman et al.	Climate Change and the Puget Sound: Building the Legal Framework for Adaptation	Center for Progressive Reform White Paper No. 1108; Lewis & Clark Law School Legal Studies Research Paper	2011	Assisted Migration	R
Glosser	Sustainable natural areas vision. Environmental Planning Solutions, Inc. for the Illinois Natural History Survey.	Available at: http://www.inhs.uiuc.edu/research/inai/SNAV_Final.pdf	2011	Assisted Migration	R
Gluesenkamp	Loss and redemption	The Ardeid	2010	Assisted Migration, Managed Relocation, Assisted Colonization	P
Goble et al.	Conservation-reliant species	BioScience	2012	Assisted Migration	A
Gobster	Alternative Approaches to Urban Natural Areas Restoration: Integrating Social and Ecological Goals	Forest Landscape Restoration	2012	Assisted Migration	B
Godbout et al.	Phylogeographic Structure of Jack Pine (Pinus banksiana; Pinaceae) Supports the Exsitence of a Coastal Glacial Refugium in Northeastern America.	American Journal of Botany	2010	Assisted Migration	A
Godefroid and Vanderborght	Plant reintroductions: the need for a global database	Biodiversity and Conservation	2011	Benign Introduction	A

Goldblum et al.	Environmental determinants of tree species distributions in central Ontario, Canada	Physical Geography	2010	Assisted Migration, Assisted Colonization	A
Gonzales	Climate change and tourism: comparing female Costa Rican and American perceptions	Worldwide Hospitality and Tourism Themes	2012	Assisted Migration	A
Gonzales	Impacts of Climate Change on Terrestrial Ecosystems and Adaptation Measures for Natural Resource Management	Changing Climates, Earth Systems and Society	2010	Managed Relocation	B
Gonzales Paredes	Conservation of Crop Wild Relative Species in Bolivia An Outline to Identify Favorable and Unfavorable Factors to Support a Conservation Program	Available at: http://repository.asu.edu/attachments/56564/content/GonzalezParedes_asu_0010N_10615.pdf	2011	Assisted Migration	T
**Goodman et al.**	**Differential Response to Soil Salinity in Endangered Key Tree Cactus: Implications for Survival in a Changing Climate**	**PLOS ONE**	**2012**	**Managed Relocation**	**A**
Goodman et al.	Climate change threatens endangered key tree cactus (Pilosocereus robinii) in the Florida Keys, USA	Available at: http://eco.confex.com/eco/2009/techprogram/P19008.HTM	2009	Assisted Migration	P
Gordon et al.	Engaging with geodiversity: why it matters	Proceedings in the Geologists' Association	2012	Managed Relocation	A
Gore et al.	Exploring the ethical basis for conservation policy: the case of inbred wolves on Isle Royale, USA	Conservation letters	2011	Assisted Migration	A
**Gould et al.**	**Growth phenology of coast Douglas-fir seed sources planted in diverse environments**	**Tree Physiology**	**2012**	**Assisted Migration**	**A**
Gozlan	Response by R Gozlan Biodiversity crisis and the introduction of non-native fish: Solutions, not scapegoats	Fish and Fisheries	2009	Assisted Species Relocation	A
**Grady et al.**	**Genetic variation in productivity of foundation riparian species at the edge of their distribution: implications for restoration and assisted migration in a warming climate**	**Global Change Biology**	**2011**	**Assisted Migration**	**A**
Graham	Snakes on a Plain, or in a Wetland: Fighting Back Invasive Nonnative Animals - Proposing a Federal Comprehensive Invasive Nonnative Animal Species Statute	Tulane Environmental Law Journal	2011	Assisted Migration	P
Graham et al.	Species Distribution Modeling and the Challenge of Predicting Future Distributions	Herzog et al. (eds): Climate Change and Biodiversity in the Tropical Andes	2011	Assisted Migration	B
**Gray and Hamann**	**Strategies for Reforestation under Uncertain Future Climates: Guidelines for Alberta, Canada**	**PLOS ONE**	**2011**	**Assisted Migration**	**A**
Gray and Hamann	Managing Western Redcedar and Yellow-Cedar in a changing environment	Harrinton: A tale of two cedars	2010	Assisted Migration	P
**Gray et al.**	**Assisted migration to address climate change**	**Ecological applications**	**2011**	**Assisted Migration**	**A**
**Green at al.**	**The Good, the Bad and the Recovery in an Assisted Migration**	**PLOS ONE**	**2010**	**Assisted Migration**	**A**
Griesbauer and Green	Assessing the climatic sensitivity of Douglas-fir at its northern range margins in British Columbia, Canada	Trees	2010	Faclitated Migration	A

Griffiths et al.	The Welfare Implications of Using Exotic Tortoises as Ecological Replacements	PLOS ONE	2012	Assisted Colonization	A
Grossnickle and Russell	Physiological variation among western redcedar (Thuja plicata Donn ex D. Don) populations in response to short-term drought	Annals of Forest Science	2010	Assisted Migration	A
Guerrant	Characterizing two decades of rare plant reintroductions	Plant reintroduction in a changing climate	2012	Managed Relocation	B
Gugger et al.	Phylogeography of Douglas-fir based on mitochondrial and chloroplast DNA sequences: testing hypotheses from the fossil record	Molecular ecology	2010	Human Assisted Migration	A
Guilbault et al.	The influence of chilling requirement on the southern distribution limit of exotic Russian olive (Elaeagnus angustifolia) in western North America	Biological invasions	2012	Transformative Restoration	A
Gunn et al.	Forestry Adaptation and Mitigation in a Changing Climate	Available at: http://www.manomet.org/sites/manomet.org/files/scidocs-pdfs/Manomet_ForestryAdaptationtoCCReport2009.pdf	2009	Assisted Migration	R
Gusset et al.	Establishment probability in newly founded populations	BMC Research Notes	2012	Assisted Colonization	A
Haeussler	Climate Change Adaptation for British Columbia Forest and Range Ecosystems: An Analysis of Existing Research Frameworks and Research Needs for the Future Forest Ecosystem Scientific council (FFESC)	NRES Occasional Paper Series	2010	Assisted Migration	R
Haeussler	Taking ecology's temperature in a warming world	LINK News	2010	Assisted Migration	P
Haeussler and Thorpe (eds.)	Complexity science and global change workshop summary	NRES Occasional Paper Series	2010	Assisted Migration	R
Haeussler and Hamilton	Informing Adaptation of British Columbia’s Forest and Range Management Framework to Anticipated Effects of Climate Change: A Synthesis of Research and Policy Recommendations	Available at: http://www.for.gov.bc.ca/ftp/HFP/external/!publish/Web/FFESC/closing-conference/FFESCsynthesis121107.docx	2012	Assisted Migration	R
Hagen et al.	Chapter 31. Contribution of Landscape Design to Changing Urban Climate Conditions	Hagen & Stiles: Urban Biodiversity and Design	2010	Assisted Migration	B
Hagerman	Adapting conservation policy to the impacts of climate change: an integrated examination of ecological and social dimensions of change	Available at: https://circle.ubc.ca/handle/2429/7903	2009	Assisted Migration	T
Hagerman and Chan	Climate change and biodiversity conservation: impacts, adaptation strategies and future research directions	Biology reports	2009	Assisted Colonization	A
Hagerman et al.	Integrative propositions for adapting conservation policy to the impacts of climate change	Global Environmental Change	2010	Assisted Migration	A
Hagerman et al.	Expert views on biodiversity conservation in an era of climate change	Global Environmental Change	2010	Assisted Migration	A

Hagermann et al. Climate change impacts, conservation and protected values	Understandig promotion, ambivalence and resistance to policy change at the world conservation congress	Conservation and society	2010	Assisted Migration	A
Haig et al.	Genetic Applications in Avian Conservation	The Auk	2011	Assisted Migration, Assisted Colonization	A
Halosksy	Adapting natural resource management to climate change: The Olympic case study	Available at: http://eco.confex.com/eco/2010/techprogram/P26760.HTM	2010	Assisted Migration	P
Halpin	GLOBAL CLIMATE CHANGE AND NATURAL-AREA PROTECTION	Ecological applications	1997	Assisted Species Relocation	A
Hannah	Climate Change, Connectivity, and Conservation	Conservation Biology	2011	Managed Relocation	A
Hannah	A global Conservation system for climate-change adaption	Conservation Biology	2010	Assisted Migration	A
**Hannah**	**Protected Areas and climate change**	**Annals of the New York Academy of Sciences**	**2008**	**Assisted Migration**	**A**
Hannah et al.	Conservation of Biodiversity in a Changing Climate	Conservation Biology	2002	Artificial Translocation	A
**Hansen**	**On the use of taxon substitutes in rewilding projects on islands**	**Islands and evolution**	**2010**	**Assisted Migration**	**A**
Hansen	Can we avert dangerous climate change	Available at: http://arxiv.org/abs/0706.3720	2007	Assisted Migration	P
Hansen	State of the Wild: Perspective of a Climatologist	Available at: http://www.davidkabraham.com/Gaia/Hansen%20State%20of%20the%20Wild.pdf	2007	Assisted Migration	P
Hansen	Buying Time.	Available at: http://stage.greenbiz.com/sites/default/files/document/CustomO16C45F64221.pdf#page=9	2003	Assisted Migration	R
Hansen and Hoffmann	Climate Savvy: Adapting Conservation and Resource Management to a Changing World	Hansen & Hoffmann: Climate Savvy: Adapting Conservation and Resource Management to a Changing World	2010	Assisted Migration	B
Hansen et al.	Ecological history and latent conservation potential: large and giant tortoises as a model for taxon substitutions	Ecography	2010	Benign Introduction, Conservation Introduction	A
Harris et al.	Ecological Restoration and Global Climate Change	Restoration Ecology	2006	Benign Introduction	A
Harrison	Favorable environments and the persistence of naturally rare species	Conservation Letters	2008	Assisted Migration	A
Harrison and Rajakaruna	What have we learned from Serpentine uín Evolution, Ecology, and other Sciences?	Harrison & Rajakaruna: Serpentine: The Evolution and Ecology of a Model System	2011	Managed Relocation	B
Harrison et al.	Climate Change and the Future of California's Serpentine Flora: Using Geologic and Soil Information to Improve Conservation Strategies	2006-2011 Mission<br>Kearney Foundation of Soil Science: Understanding and Managing Soil-Ecosystem<br>Functions Across Spatial and Temporal Scales<br>Progress Report: 2009022, 1/1/2010-12/31/2010	2009	Assisted Relocation	R

Harrop	Climate change, conservation and the place for wild animal welfare in international law	Journal of Environmental law	2011	Assisted Migration	A
Haskins and Keel	Managed reolocation: Panacea or Pandemonium?	Plant reintroduction in a changing climate	2012	Managed Relocation	B
Haskins and Pence	Transitioning Plants to New Environments: Beneficial Applications of Soil Microbes	Plant reintroduction in a changing climate	2012	Managed Relocation	B
Hastie et al.	The Threat of Climate Change to Freshwater Pearl Mussel Populations	AMBIO: A Journal of the Human Environment	2003	Artificial Translocation	A
Haughian	Expected Effects of Climate Change on Forest Distrurbance in Bristish Columbia	Journal of Environmental Monitoring	2012	Facilitated Migration	P
Hayashi	Climate-induced Tree Migration in Southern Ontario: Pathways and Source Populations	Available at: https://tspace.library.utoronto.ca/bitstream/1807/29556/1/Hayashi_Kaho_20116_MScF_thesis.pdf	2011	Assisted Migration	T
Hayward	Time to agree on a conservation benchmark for Australia	Pacific Conservation Biology	2012	Assisted Migration	A
**Hayward**	**Conservation management for the past, present and future**	**Biodivers Conserv**	**2009**	**Assisted Migration, Assisted Range Expansion**	**A**
Hebda	The Impacts of Climate Change on the Flora of the Canadian Southern Rocky Mountain Region and its Value to Conservation	Available at: http://ww.cpawsbc.org/files/Media/Terrestrial%20Publications/CPAWS_Flathead_Climate%20Rockies_flora_comp_Mar2010.pdf	2010	Assisted Migration	R
Heffernan	It's conservation - but not as we know it	Nature climate change	2008	Assisted Migration	A
Hein et al.	Dispersal through stream networks: modelling climate-driven range expansions of fishes	Diversity and Distributions	2011	Human Assisted Migration, Assisted Migration	A
Heller and Zaveleta	Biodiversity management in the face of climate change: A review of 22 years of recommendations	Biological Conservation	2009	Translocation	A
Hellman et al.	Five Potential Consequences of Climate Change for Invasive Species	Conservation Biology	2008	Assisted Migration	A
Hellman et al.	The response of two butterfly species to climatic variation at the edge of their range and the implications for poleward range shifts	Oecologia	2008	Assisted Migration	A
Hellmann	Future Human Intervention in Ecosystems and the Critical Role for Evolutionary Biology	Conservation Biology	2011	Managed Relocation	A
Hellmann	Managing Nature as Earth Warms	Nature	2009	Assisted Migration	A
Hellmann and Marsico	Range Limitation and Dispersal of Plants	Available at: http://publications.gc.ca/collections/collection_2012/dn-nd/D3-21-2009-eng.pdf#page=38	2009	Assisted Migration	R
Hellmann et al.	Strategies for Reducing Extinction Risk under a Changing Climate	Saving a Million species	2012	Managed Relocation	B
Hellmann et al.	The influence of species interactions on geographic range change under climate change	Annals of the New York Academy of Sciences	2012	Managed Relocation	A
Heneghan et al.	Lessons Learned from Chicago Wilderness—Implementing and Sustaining Conservation Management in an Urban Setting	Diversity	2012	Assisted Migration	A

Henle et al.	Climate Change impacts on European amphibians and reptiles	Council of Europe: Biodiversity and climate change: Reports and guidance developed under the Bern Convention - Volume I (Nature and Environment N°156) (2010)	2008	Assisted Dispersal	R
Hennon	Shifting Climate, Altered Niche, and a Dynamic Conservation Strategy for Yellow Cedar in the North Pacific Coastal Rainforest	BioScience	2012	Assisted Migration	A
Hermy	Lessons from the past to the future. Future needs for forest plants.	Book of abstracts of the IUFRO Landscape Ecology Working Group International Conference: Forest	2010	Assisted Migration, Managed Relocation	P
Hermy et al.	Forest ecosystem assessment, changes in biodiversity and climate change in a densely populated region (Flanders, Belgium)	Plant biosystems	2008	Assisted Migration	A
Hermy et al.	Jumping the garden fence gives a head start for plants on climate change	Available at: http://opus.kobv.de/zlb/volltexte/2008/6685/pdf/skript229_1.pdf	2008	Assisted Migration	P
**Hewitt et al.**	**Taking stock of the assisted migration debate.**	**Biological Conservation**	**2011**	**Assisted Migration**	**A**
Heywood	The role of botanic gardens as resource and introduction centres in the face of global change	Biodiversity and Conservation	2011	Assisted Migration	A
Hiddink et al.	Keeping pace with climate change: what can we learn from the spread of Lessepsian migrants?	Global Change Biology	2012	Assisted Colonization	A
Hiller	Oregon Furbearer Program Report	Available at: http://www.dfw.state.or.us/resources/hunting/small_game/docs/2011_furbearer_report.pdf	2011	Assisted Migration	R
Hilty et al.	Moving Forward on Climate Change Science, Planning, and Action	Climate and conservation	2012	Translocation	B
Hoag	Confronting the biodiversity crisis	Nature	2010	Managed Relocation, Assisted Relocation, Assisted Migration	P
Hobbs	What do we know about, and what do we do about, novel ecosystems	Novel Ecosystems: Intervening in the New Ecological World Order	2012	Assisted Migration, Managed Relocation	B
Hobbs et al.	Guiding concepts for park and wilderness stewardship in an era of global environmental change	Frontiers in Ecology	2010	Assisted Migration	A
Hobbs et al.	Novel ecosystems: implications for conservation and restoration	Trends in Ecology and Evolution	2009	Assisted Migration	A
Hobday	Sliding baselines and shuffling species: modelling climate-driven range expansions of fishes	Marine ecology	2011	Assisted Migration	A
Hobday et al.	Enhancing Species Adaption to Climate Change	Poloczanska et al.: A Marine Climate Change Impacts and Adaptation Report Card for Australia 2009	2009	Assisted Dispersal, Assisted Translocation	R
Hodgkin and Bordoni	Climate Change and the Conservation of Plant Genetic Resources	Journal of crop improvement	2012	Assisted Migration	A
**Hoegh-Guldberg et al.**	**Assisted Colonization and Rapid Climate Change**	**Science**	**2008**	**Assisted Colonization**	**A**
**Hoegh-Guldberg et al.**	**Response to letters**	**Science**	**2008**	**Assisted Colonization**	**A**

Hoffman et al. (eds.)	CETACEANS & Other Marine Biodiversity of the Eastern Tropical Pacific. Options for Adapting to Climate Change	Available at: http://www.cakex.org/sites/default/files/taller_cetaceos_etp_y_adaptacion_informe_4_junio_09_1.pdf#page=9	2009	Assisted Migration	R
Hogg and Bernier	Climate change impacts on drought-prone forests	The Forestry Chronicle	2005	Assisted Migration	P
Hole et al.	Adaptive Management for Biodiversity Conservation under Climate Change – a Tropical Andean Perspective	Available at: http://www.iai.int/files/communications/publications/scientific/Climate_Change_and_Biodiversity_in_the_Tropical_Andes/chapter2.pdf	2011	Assisted Migration, Translocation	B
Hollstedt	No one said it was going to be easy	Journal of Ecological Monitoring	2011	Assisted Migration	P
Holmes	Assisted migration: helping nature to relocate	New scientist	2007	Assisted Colonization	P
Holsman et al.	Interacting Effects of Translocation, Artificial Propagation, and Environmental Conditions on the Marine Survival of Chinook Salmon from the Columbia River, Washington, U.S.A.	Conservation Biology	2012	Assisted Colonization	A
Holt et al.	Assessment and Decision-making for Climate Change	Available at: http://www.westkootenayresilience.org/Report2_Approaches_Final.pdf	2012	Assisted Migration	R
Hooper	Climate Change Impacts and Forest Management Adaptation Measures in Sweden and British Columbia, Canada	Available at: http://stud.epsilon.slu.se/4530/7/hooper_r_120710.pdf	2012	Assisted Migration	T
Hrynkiewicz-Moczulski	Population variation	Available at: https://circle.ubc.ca/bitstream/handle/2429/27237/HrynkiewiczMoczulskiMagdalena%20_FRST%20498_Graduating_Thesis_2008.pdf?sequence=1	2009	Facilitated Migration	T
**Huang**	**Assisted Colonization won't help rare species**	**Science**	**2008**	**Assisted Colonization**	**A**
Hubert	The Ecological and Conservation Genetics of Garry.	Available at: https://dspace.library.ubc.ca/bitstream/handle/2429/12401/ubc_2009_fall_huebert_colin.pdf?sequence=1	2009	Facilitated Migration	T
Hughes	Climate Change	Lindenmayer et al.: Ten Commitments: Reshaping the Lucky Country's Environment	2008	Assisted Migration	B
Hughson et al.	Natural resource mitigation, adaptation and research needs related to climate change in the Great Basin and Mojave Desert: Workshop summary	Available at: http://pubs.usgs.gov/sir/2011/5103/pdf/sir20115103.pdf	2011	Assisted Migration	R
Hunt et al.	White pines, Ribes, and blister rust: integration and action	Forest pathology	2010	Assisted Migration	A
**Hunter**	**Emerging Landscapes: Using Ecological Theory to Guide Urban Planting Design: An adaptation strategy for climate change**	**Landscape Journal**	**2011**	**Assisted Migration**	**P**

**Hunter**	**Climate change and Moving Species: Furthering the deabte on Assisted Colonization**	**Conservation Biology**	**2007**	**Assisted Migration**	**A**
Huntley et al.	Potential impacts of climatic change upon geographical distributions of birds	Ibis	2006	Artificial Translocation	A
Ieronymidou et al.	Endemic Cyprus Warbler Sylvia melanothorax and colonizing Sardinian Warbler Sylvia melanocephala show different habitat associations	Ibis	2012	Assisted Colonization	A
Iguchi J.	Improving the Improvement Act: Climate Change Management in the National Wildlife Refuge System	Environs: Environmental Law and Policy Journal	2011	Assisted Migration	P
Irl and Beierkuhnlein	Distribution of endemic plant species on an oceanic island – a geospatial analysis of La Palma (Canary Islands)	Procedia Environmental Sciences	2011	Assisted Migration	P
Isaac	Saving million species: extinction risk from climate change	Pacific Conservation Biology	2012	Managed Relocation	A
Isaak et al.	Effects of climate change and wildfire on stream temperatures	Ecological applications	2010	Human Assisted Migration	A
Iverson et al.	Development of risk matrices for evaluating climatic change responses of forested habitats	Climatic Change	2012	Assisted Migration	A
Iverson et al.	Lessons learned while integrating habitat, dispersal, disturbance, and life-history traits into species habitat models under climate change	Ecosystems	2011	Human Assisted Migration	A
Iverson et al.	Merger of three modeling approaches to assess potential effects of climate change	Forest Landscapes and Global Change-New Frontiers in Management, Conservation and Restoration. Proceedings of the IUFRO Landscape Ecology	2010	Assisted Migration	P
Jackson and Sax	Balancing biodiversity in a changing environment: extinction dept, immigration credit and species turnover	Trends in Ecology and Evolution	2010	Managed Relocation	A
Jalili et al.	Climate change, unpredictable cold waves and possible brakes on plant migration	Global Ecology and Biogeography	2010	Human Assisted Migration	A
James	CSIRO Submission 11/417 Australia's biodiversity in a changing climate	Available at: http://202.14.81.34/house/committee/ccea/ccbio/subs/Sub023.pdf	2011	Assisted Translocation	R
Jamieson et al.	Managing genetic diversity in threatened populations: a New Zealand perspective	New Zealand journal of Ecology	2008	Human Assisted Translocation	A
Janowiak et al.	Silvicultural decisionmaking in an uncertain climate future: a workshop-based exploration of considerations, strategies, and approaches	Available at: http://www.treesearch.fs.fed.us/pubs/37881	2011	Assisted Migration	R
Jaquish	Review of British Columbia's contemporary and projected western larch seed planning zones in light of climate change	Available at: http://bcwildfire.ca/ftp/HTI/external/!publish/Interim_Measures/Lw_Review_March_19_2010/INTERIM_Lw_Report_%28Jaquish%29_FINAL.pdf	2010	Assisted Migration, Facilitated Migration	R

Jenerette	Experimental landscape ecology	Landscape ecology	2012	Assisted Migration	A
Jensen et al.	A research approach supporting domestication of Baobab (Adansonia digitata L.) in West Africa	New Forests	2011	Assisted Migration	A
Jorgensen et al.	Regional patterns of vascular plant diversity and endemism	Herzog et al. (eds): Climate Change and Biodiversity in the Tropical Andes	2011	Assisted Migration	B
Jotzo	**A perspective paper on adaptation as a response to climate change.**	Available at: http://fixtheclimate.com/uploads/tx_templavoila/PP_Adaptation_Jotzo_v.2.0.pdf	2009	Assisted Relocation	R
Joyce et al.	Analysis of Potential Impacts of Climate Change on Wildlife Habitats in the U.S.	Linda A Joyce US Forest Service Rocky Mountain Research Station 240 West Prospect, Fort Collins, CO 80526 Curtis H Flather US Forest Service Rocky …	2008	Assisted Migration	R
**Jørgensen**	**What's History Got to Do with It? A Response to Seddon's Definition of Reintroduction**	**Restoration ecology**	**2011**	**Assisted Colonization**	**A**
**Kabaz-Gomez**	**Rules for Playing God: The Need for Assisted Migration & New Regulation**	**Animal Law**	**2012**	**Assisted Migration**	**A**
Kalk	The role of seed banks in coastal plant community response to climate change: Implications for restoring ecosystem resiliency	Available at: http://opensiuc.lib.siu.edu/cgi/viewcontent.cgi?article=1749&context=theses	2011	Assisted Migration	T
Kates et al.	Transformational adaptation when incremental adaptations to climate change are insufficient	Proceedings of the National Academy of Sciences	2012	Assisted Colonization	A
Kawelo et al.	Unique Reintroduction Considerations in Hawaii: Case Studies from a Decade of Rare Plant Restoration at the Oahu Army Natural Resource Rare Plant Program	Plant reintroduction in a changing climate	2012	Managed Relocation	B
Keel	Assisted migration as a conservation strategy for rapid climate change: Investigating extended photoperiod and mycobiont distributions of Habenaria repens Nuttall (Orchidaceae) as a case study	Available at: http://gradworks.umi.com/32/63/3263199.html	2007	Assisted Migration	T
**Keel et al.**	**Seed Germination of Habenaria repens (Orchidaceae) in situ Beyond its Range, and its Potential for Assisted Migration Imposed by Climate Change**	**Castanea The Journal of the Southern Appalachian Botanical Society**	**2011**	**Assisted Migration**	**A**
Kekkonen et al.	Heterozygosity in an Isolated Population of a Large Mammal Founded by Four Individuals Is Predicted by an Individual-Based Genetic Model	PLOS ONE	2012	Assisted Migration	A
Kennedy et al.	Synthesis and Future Directions	Plant reintroduction in a changing climate	2012	Managed Relocation	B
Kibel	Climate Adaptation Policy at the Continental Level: Natural Resources in North American and Europe	Pace Environmental Law Review	2010	Assisted Migration	A
Kingsford and Watson	Climate Change in Oceania-A synthesis of biodiversity impacts and adaptations.	Pacific Conservation Biology	2011	Translocation	A
Kittel et al.	A vulnerability-based strategy for incorporating the climate threat in conservation planning: A case study from the British Columbia Central Interior	Journal of Ecological Monitoring	2011	Assisted Migration	R

**Kjaer et al.**	**Will the Danish forests stay adapted?**	**IOP Conf. Series: Earth and Environmental Science 6**	**2009**	**Assisted Migration**	**P**
Klenk and Larson	Conflicting modes of reasoning in the Assisted Migration debate: a concept mapping analysis	Available at: http://adsabs.harvard.edu/abs/2011AGUFM.B13C0571K	2011	Assisted Migration	P
Klenk et al.	The "emulation of natural disturbance" (END) management	The Forestry Chronicle	2009	Assisted Migration	P
Knight	Using Population Viablity Analysis to Plan Reintroductions	Plant reintroduction in a changing climate	2012	Managed Relocation	B
Knowlton et al.	Using behavioral landscape ecology to predict species' responses to land-use and climate change	Biological conservation	2010	Assisted Migration	A
Knutson and Heglund	Resource Managers Rise to the Challenge of Climate Change	Beever: Ecological Consequences of Climate Change: Mechanisms, Conservation, and	2011	Assisted Dispersal	B
Koch et al.	Isotopic and genetic insights into the persistence of the northern fur seal (Callorhinus ursinus)	Available at: http://adsabs.harvard.edu/abs/2010AGUFM.B11H..06K	2010	Assisted Migration	P
Koehn et al.	Climate change and Australian marine and freshwater environments, fishes and fisheries: synthesis and options for adaptation	Marine and Freshwater Research	2011	Assisted Migration	A
Kolden et al.	Mapped versus actual burned area within wildfire perimeters: Characterizing the unburned	Forest Ecology and Management	2012	Assisted Migration	A
**Kostyack et al.**	**Beyond Reserves and Corridors: Policy Solutions to Facilitate the Movement of Plants and Animals in a Changing Climate**	**Bioscience**	**2011**	**Assisted Colonization**	**A**
Kowarik et al.	Plants in Urban Settings: From Patterns to Mechanisms and Ecosystem Services	Endlicher: Perspectives in Urban Ecology: Ecosystems and Interactions…	2011	Assisted Migration	B
Kozak et al	Current conservation strategies for European crayfish	Knowledge and management of aquatic ecosystems	2011	Assisted Migration	P
Kramer and Havens	Plant conservation genetics in a changing world	Trends in plant science	2009	Assisted Migration	A
**Kranabetter et al.**	**Divergence in ectomycorrhizal communities with foreign Douglas-fir populations and implications for assisted migration**	**Ecological Applications**	**2012**	**Assisted Migration**	**A**
Krause	Strategic Decisions in Conservation: Using Species Distribution Modeling to Match Ecological Requirements to Available Habitat	Plant reintroduction in a changing climate	2012	Managed Relocation	B
Krause	Conservation ecology of endemic plants of the Colorado Plateau: Climate change impacts on range shifts	Available at: http://gradworks.umi.com/34/25/3425574.html	2010	Assisted Migration	T
Kreeger and Kraeuter	Ecologically Significant Bivalve Molluscs of the Delaware Estuary	Climate Change and the Delaware Estuary	2010	Assisted Migration	R
Kremer et al.	Forest ecosystem genomics and adaptation: EVOLTREE conference report	Tree Genetics & Genomes	2011	Assisted Migration	P

Kreyling et al.	Late frost sensitivity of juvenile Fagus sylvatica L. differs between southern Germany and Bulgaria and depends on preceding air temperature	European Journal of Forest Research	2012	Assisted Colonization	A
Kreyling et al.	Geographic origin and past climatic experience influence the response to late spring frost in four common grass species in central Europe	Ecography	2012	Assisted Colonization	A
**Kreyling et al.**	**Assisted colonization: A question of focal units and recipient localities**	**Restoration ecology**	**2011**	**Assisted Colonization**	**A**
Kriticos	CliMond: global high-resolution historical and future scenario climate surfaces for bioclimatic modelling	Methods in Ecology and Evolution	2012	Managed Relocation	A
Krosby et al.	Planning for landscape connectivity in a changing climate	Available at: http://eco.confex.com/eco/2010/techprogram/P22026.HTM	2010	Assisted Migration	P
Krosby et al.	Ecological Connectivity for a Changing Climate	Conservation Biology	2010	Assisted Migration	A
Krutovsky et al	Gene Flow, Spatial Structure, Local Adaptation, and Assisted Migration in Trees	Genomics of Tree Crops	2012	Assisted Migration	B
Kueffer and Daehler	A Habitat-Classification Framework	Inderjit (ed.): Management of Invasive Weeds	2009	Assisted Migration	B
Kätzel and Höppner	Adaptation strategies in forest management	Folia Forestalia Polonica	2011	Facilitated Migration	P
Lackstrom et al.	Engaging Climate-Sensitive Sectors	Available at: http://www.cisa.sc.edu/Pubs_Presentations_Posters/Reports/2012_Lackstrom%20et%20al_Engaging%20Climate-Sensitive%20Sectors%20in%20the%20Carolinas.pdf	2012	Human Assisted Migration	R
Ladle and Whittaker	Conservation Biogeography	Ladle & Whittaker: Conservation Biogeography	2011	Managed Relocation	B
Lamb	Vegetation Biodiversity Survey and Seed Rain Assessment as part of long term reforestration monitoring at the cloud forest school in Monteverde, Costa Rica	Available at: http://www.etd.ceu.hu/2012/lamb_lisa.pdf	2012	Assisted Migration	T
Landguth et al.	UNICOR: a species connectivity and corridor network simulator	Ecography	2012	Assisted Migration	A
Landis et al.	Insect conservation in Michigan prairie fen: addressing the challenge of global change	Journal of Insect Conservation	2012	Assisted Migration	A
Lankau et al.	Incorporating evolutionary principles into environmental management and policy	Evolutionary applications	2011	Managed Translocation	A
Lauteri et al.	Preservation of Chestnut (Castanea sativa Mill.) Genetic Resources and Adaptive Potential in Relation to Environmental Changes	Proc. 1st European Congress on Chestnut - Castanea 2009	2009	Assisted Migration	P
Lawler	Projected Climate Impacts for the Amphibians of the Western Hemisphere	Conservation Biology	2010	Translocation	A
Lawler	Climate Change Adaptation Strategies for Resource Management and Conservation Planning	Annals of the New York Academy of Sciences	2009	Assisted Migration	A

**Lawler andOlden**	**Reframing the debate over assisted colonization**	**Frontiers in Ecology and the Environment**	**2011**	**Assisted Colonization**	**A**
Lawler et al.	Resource management in a changing and uncertain climate	Frontiers in Ecology	2010	Translocation	A
Laws et al.	A Bayesian network approach for selecting translocation sites for endangered island birds	Biological Conservation	2012	Assisted Colonization	A
Lawson et al.	Local and landscape management of an expanding range margin under climate change	Journal of Applied Ecology	2012	Assisted Colonization	A
Leadly	Biodiversity scenarios: projections of 21st century change in biodiversity	Leadly: Biodiversity scenarios: projections of 21st century change in biodiversity	2010	Assisted Migration	B
Leary et al.	A Stitch in Time: Lessons for Climate Change	Available at: http://www.idcoa.cloverpad.org/resources/Documents/Climate_Change%20_Adaptation_AIACC_WP48_Leary_etal.pdf	2007	Facilited Migration	R
**Ledig**	**Climate Change and Conservation**	**Acta Silvatica & Lingaria Hungarica**	**2012**	**Assisted Colonization**	**A**
**Ledig et al.**	**Projections of Suitable Habitat Under Climate Chnage Scenarios: Implications for Trans-Boundary Assisted Colonization.**	**American Journal of Botany**	**2012**	**Assisted Colonization**	**A**
**Ledig et al.**	**Projections of suitable habitat for rare species under global warming scenarios**	**American journal of botany**	**2010**	**Assisted Colonization**	**A**
**Leech et al.**	**Assisted Migration: Adapting forest**	**BC Journal of Ecosystems and Management**	**2011**	**Assisted Migration**	**A**
Legget	Climate change. Current issues and policy tools	Available at: http://assets.opencrs.com/rpts/RL34513_20090306.pdf	2009	Assisted Migration	R
Leites et al.	Height-growth response to climatic changes differs among populations of Douglas-fir: a novel analysis of historic data	Ecological Applications	2012	Assisted Migration	A
Lemieux and Scott	Changing Climate, Challenging Choices: Identifying and Evaluating Climate Change Adaptation Options for Protected Areas Management in Ontario, Canada	Environmental Management	2011	Assisted Migration	A
Lemieux and Thompson	Multi-scale Challenges to Efficient and Effective Adaptation to Climate Change in North American Protected Areas Agencies	Available at: http://www.cfc.umt.edu/cesu/NEWCESU/Assets/Individual%20Project%20Reports/NPS%20Projects/CSU/2010/10Manfredo_BRMD_CC%20and%20social%20science_journal%20article%20draft.pdf	2010	Assisted Colonization	A
Lemieux et al.	Prospects for Canada's protected areas in an era of rapid climate change	Land Use Policy	2011	Assisted Migration	A
LePage	Wetlands: Integrating Multidisciplinary Concepts	LePage: Wetlands:Integrating Multidisciplinary Concepts	2010	Assisted Migration	B
Leshy	Federal Lands in the Twenty-First Century	Natural Resources Journal	2010	Assisted Migration	A
Lewis et al.	Carnivore Translocations and Conservation: Insights from Population Models and Field Data for Fishers (Martes pennanti)	PLOS ONE	2012	Assisted Colonization	A

Libby	The next 30 years	Proceedings of the IUFRO Division 2 Joint Conference: Low<br>Input Breeding and Conservation of Forest Genetic Resources: Antalya, Turkey, 9-13<br>October 2006. Available at: http://www.akdeniz.edu.tr/english/iufro/	2006	Assisted Migration	P
Light	Finding a Future for Environmental Ethics	The ethics forum	2012	Assisted Migration	P
**Light and MacConall**	**Potential impact of insect herbivores on orchid conservation**	**European Journal of Environmental Sciences**	**2011**	**Assisted Migration**	**A**
Lin	Comparative Reproductive Biology of a Rare Endemic Orchid and its Sympatric Congeners in Southwestern China	Available at: http://digitalcommons.fiu.edu/etd/570/?utm_source=digitalcommons.fiu.edu%2Fetd%2F570&utm_medium=PDF&utm_campaign=PDFCoverPages	2012	Managed Relocation	T
Linklater et al.	Translocations as experiments in the ecological resilience of an asocial mega-herbivore	PLOS ONE	2012	Assisted Migration	A
**Liu et al.**	**Overcoming extreme weather challenges: Successful but variable assisted colonization of wild orchids in southwestern China**	**Biological Conservation**	**2012**	**Assisted Colonization**	**A**
Liu et al.	Potential Challenges of Climate Change to Orchid Conservation in a Wild Orchid Hotspot in Southwestern China	The Botanical Review	2010	Assisted Migration, Human Assisted Migration	A
Livezy	Killing barred owls to help spotted owls I: a global perspective	Northwestern Naturalist	2010	Assisted Migration	A
Lo et al.	A word of caution when planning forest management	The Forestry Chronicle	2010	Assisted Colonization, Assisted Migration	P
Loarie	Conserving Moving Species under Changing Landscapes and Climates.	Available at: http://dukespace.lib.duke.edu/dspace/bitstream/handle/10161/700/D_Loarie_Scott_a_200808.pdf?sequence=1	2008	Human Assisted Dispersal	T
Loarie et al.	The velocity of Climate change	Nature	2009	Managed Relocation	A
Loarie et al.	Climate Change and the Future of California’s Endemic	PLOS ONE	2008	Human Assisted Dispersal	A
Long and Mock	Changing perspectives on regeneration ecology	Canadian journal of forest research	2012	Assisted Migration	A
Loo et al.	Climate Change and Forest genetic Resources - State of Knowledge, Risks and Opportunities.	Available at: http://www.prodinra.inra.fr/prodinra/pinra/data/2011/07/PROD2011c2dace48_20110719111005803.pdf	2011	Assisted Migration	R
Lorimer	Multinatural geographies for the Anthropocene	Progress in Human Geography	2012	Assisted Migration	A
Losleben and Weltzin	Monitoring the Pulse of Our Planet—The USA-National	Mountain Views<br>The Newsletter of the Consortium for Integrated Climate Research in Western Mountains<br>CIRMOUNT	2008	Assisted Migration	P
**Loss et al.**	**Assisted colonization: integrating conservation strategies in the face of climate change**	**Biological conservation**	**2011**	**Assisted Colonization**	**A**

Lu and Man	Assesment of assisted migration effects on spring bud flush	The Forestry Chronicle	2011	Assisted Migration	P
Lundy	Climate Change and Endangered Species in Canada: A screening level impact assessment and analysis of species at risk management and policy.	Available at: http://www.uwspace.uwaterloo.ca/bitstream/10012/4169/1/Lundy_K%20-%20MES%20Thesis%20-%20Dec%2008%20-%20FINAL.pdf	2008	Assisted Colonization	T
Lybolt et al.	Instability in a marginal coral reef: the shift from natural variability to a human-dominated seascape	Frontiers in Ecology	2011	Assisted Colonization	A
Lydon	Climate Change's Impact on Western North American Butterflies	Available at: http://s3.amazonaws.com/academia.edu.documents/30547166/WritingSample_GEOG6660W12ETLydon.pdf?AWSAccessKeyId=AKIAIR6FSIMDFXPEERSA&Expires=1368205251&Signature=eMOUZwbsTL2B8UFSAFh8sOyMQb8%3D&response-content-disposition=inline	2012	Assisted Migration	T
Ma et al.	Somatic embryogenesis, plant regeneration, and cryopreservation for Torreya taxifolia, a highly endangered coniferous species	In Vitro Cellular & Developmental Biology	2012	Assisted Migration	A
Maalouf	The interplay of a climate change-induced drought and management in South European calcareous grasslands	Available at: http://ori-oai.u-bordeaux1.fr/pdf/2012/MAALOUF_JEAN-PAUL_2012.pdf	2012	Assisted Migration	T
**Maalouf et al.**	**Integrating climate change into calcareous grassland management**	**Journal of Applied Ecology**	**2012**	**Assisted Migration**	**A**
MacDonald et al.	Root growth of containerized lodgepole pine seedlings in response to Ascophyllum nodosum extract application during nursery culture	Canadian journal of plant science	2012	Assisted Migration	A
MacDonald et al.	Ecological Implications of Changing the Composition of Boreal Mixedwood Forests	Available at: http://www.ales.ualberta.ca/forestry/Sustainable_Forest_Management/Publications/~/media/University%20of%20Alberta/Faculties/ALES/Departments/Forestry/Department%20Site/Sustainable%20Forest%20Management/Documents/SFM/SFMN-KnowledgeReports/SOK2010MixedwoodsMacdonaldetalEn.pdf	2010	Assisted Migration	R
Majidian	Potential for Migration of Coastal Redwood to British Columbia	Available at: https://circle.ubc.ca/bitstream/handle/2429/36459/Majidian_Maryam_FRST_497_Graduating_Essay_2010.pdf?sequence=1	2011	Facilitated Migration	T
Mallet	Hybridisation and climate change: brown argus butter- flies in Britain (Polyommatus subgenus Aricia)	Insect Conservation and Diversity	2011	Assisted Colonization	A
Malmsheimer et al.	Forest management solutions for mitigating climate change in the United States	Available at: https://www.eforester.org/publications/jof/jof_cctf.pdf	2008	Facilitated Dispersal	B

Mantyka-Pringle et al.	Interactions between climate and habitat loss effects on biodiversity: a systematic review and meta-analysis	Global Change Biology	2012	Translocation	A
Maringer et al.	Post-fire spread of alien plant species in a mixed broad-leaved forest of the Insubric region	Flora - Morphology, Distribution, Functional Ecology of Plants	2012	Human Assisted Migration	A
Marini	Major current and future gaps of Brazilian reserves to protect Neotropical savanna birds	Biological Conservation	2009	Assisted Migration	A
Marris	A scientific argument for intervening in nature	Scientific American	2011	Assisted Migration	P
Marris	Moving on assisted migration	Nature	2008	Assisted Migration	P
Marris	Pre-emptive strike: outwitting extinction	Nature Reports Climate Change	2008	Assisted Migration	P
Marsico	Post-Glacial Migration, Limitations to Poleward Range Expansion, and Growth Responses to Future Climates of Plants in the Garry Oak System.	Available at: http://etd.nd.edu/ETD-db/theses/available/etd-12012008-112130/unrestricted/MarsicoT122008.pdf	2008	Assisted Migration	T
Marsico and Hellmann	Dispersal limitation inferred from an experimental translocation of Lomatium (Apiaceae) species outside their geographic ranges	Oikos	2009	Assisted Migration	A
Martin	Symposium summary: Re-imagining conservation goals in light of global change	Available at: http://escholarship.org/uc/item/3tq3b36w	2012	Assisted Migration	C
Martin	The conservation genetics of Ash Meadows pupfish populations. I. The Warm Springs pupfish Cyprinodon nevadensis pectoralis	Conservation Genetics	2010	Assisted Migration	A
Martin andMcdonald-Madden	Relocating Species to Ensure Survival in a Changing Climate.	Issues: Australian science	2011	Managed Relocation	P
Martin et al.	Structured decision making as a proactive approach to dealing with sea level rise in Florida	Climatic Change	2011	Assisted Migration	A
Martinez-Morales	Biodiversity Research: Current distribution and predicted geographic expansion of the Rufous-backed Robin in Mexico: a fading endemism?	Diversity and Distributions	2010	Managed Relocation	A
Maschinski et al.	The Critical Role of the Public: Plant Conservation through Volunteer and Community Outreach Projects	Plant reintroduction in a changing climate	2012	Managed Relocation	B
Maschinski et al.	Sinking ships: conservation options for endemic taxa threatened by sea level rise	Climatic Change	2011	Managed Relocation	A
Mashinski et al.	Optimal Locations for Plant Reintroductions in a Changing World	Plant reintroduction in a changing climate	2012	Managed Relocation	B
Mastrandea et al.	Bridging the gap: linking climate-impacts research with adaptation planning and management	Climatic change	2010	Managed Relocation	A
Matthews et al.	Demographic approaches to Assessing climate change impact: an application to pond-breeding frogs and shifting hydropatterns	Wildfire Conservarvation in a changing climate	2012	Assisted Migration	B
Matthews et al.	Modifying climate change habitat models using tree species-specific assessments	Forest ecology and management	2011	Assisted Migration	A
Mawdsley	Design of conservation strategies for climate adaptation	Wiley interdisiplinary reviews	2011	Translocation, Assisted Dispersal	A

Mawdsley et al.	A review of Climate-Change Adaption Strategies for Wildlife Manaagement and Biodiversity Conservation	Conservation Biology	2009	Translocation, Assisted Dispersal	A
Mbogga et al.	Bioclimate envelope model predictions for natural resource management: dealing with uncertainty	Journal of Applied Ecology	2010	Assisted Migration	A
McCallum	Tasmanian devil facial tumour disease: lessons for conservation biology	Trends in Ecology and Evolution	2008	Assisted Migration	A
McConkey et al.	Seed dispersal in chnaging landscapes	Biological conservation	2012	Assisted Migration	A
McDonald and Service	Key topics in conservation biology	McDonald & Service: Key topics in conservation biology	2007	Assisted Migration	B
**McDonald-Madden et al.**	**Optimal timing for managed relocation of species faced with climate change**	**Nature Climate Change**	**2011**	**Managed Relocation**	**A**
**McIntyre**	**Ecological and anthropomorphic factors permitting low-risk assisted colonization in temperate grassy woodlands**	**Biological Conservation**	**2011**	**Assisted Colonization**	**A**
McKenney	Potential Impacts of Climate Change on the Distribution of North American Trees	Bioscience	2007	Assisted Migration	A
McKenney and Pedlar	Practical (?) considerations for implementing assisted migration strategies for trees in North America	Available at: http://adsabs.harvard.edu/abs/2011AGUFM.B13C0570M	2011	Assisted Migration	P
McKenney et al.	Revisiting projected shifts in the climate envelopes of North American trees using updated general circulation models	Global Change Biology	2011	Assisted Migration	A
**McKenney et al.**	**Climate change and forest seed zones: Past trends, future prospects and challenges to ponder**	**Forestry chronicle**	**2009**	**Assisted Migration**	**P**
McKinnon	Climate Change	Available at: http://pamodelforest.sk.ca/pubs/ClimateChangeReality.pdf	2003	Human Assisted Migration	R
McLachlan	The limits of scientific information for informing forest policy decisions under changing climate	Available at: http://adsabs.harvard.edu/abs/2011AGUFM.B13C0572M	2011	Managed Relocation	P
McLachlan	A survey to evaluate managed relocation as an adaptation strategy	Available at: http://eco.confex.com/eco/2009/techprogram/P15594.HTM	2009	Managed Relocation	P
**McLachlan et al.**	**A Framework for Debate of Assisted Migartion in an era of climate change**	**Conservation**	**2007**	**Assisted Migration**	**A**
McLane	Establishment and growth responses of whitebark and lodgepole pine populations in a changing climate	Available at: https://circle.ubc.ca/bitstream/handle/2429/34087/ubc_2011_spring_mclane_sierra.pdf?sequence=1	2011	Assisted Migration	T

Mclane and Aitken	Whitebark pine (Pinus albicaulus) assisted migration trial. In: Keane, Robert E.; Tomback, Diana F.; Murray, Michael P.; and Smith, Cyndi M., ds. 2011. The future of high-elevation, five-needle white pines in Western North America: Proceedings of the High Five Symposium. 28-30 June 2010; Missoula, MT. Proceedings RMRS-P-63. Fort Collins, CO	Proceedings of the High Five Symposium. 28-30 June 2010	2011	Assisted Migration	P
**McLane and Aitken**	**Whitebark pine (Pinus albicaulis) assisted migration potential: testing establishment north of the species range**	**Ecological Applications**	**2012**	**Assisted Migration**	**A**
Mclane et al.	Climate impacts on lodgepole pine (Pinus contorta) radial growth in a provenance experiment	Forest ecology and management	2011	Assisted Migration	A
McLaughlin	Manipulating Nature.	New York Times	2009	Assisted Migration	P
Meadows	UC scientists help California prepare for climate change	California Agriculture	2009	Managed Relocation	P
Mercer	Evolutionary response of landraces to climate change	Evolutionary Applications	2010	Assisted Migration, Assisted Colonization	A
Metsaranta et al.	Uncertainty of 21st century growing stocks and GHG balance of forests in British Columbia, Canada resulting from potential climate change impacts on ecosystem processes	Forest ecology and management	2011	Assisted Migration	A
Meyer	Creaturely Theology: On God, Humans and Other Animals – Edited by Celia Deane-Drummond and David Clough	Reviews in Religion and Theology	2011	Assisted Migration	A
Meyers	Evolutionary Relationships and an Investigation of Sympatric Speciation within Limnanthaceae	Available at: http://ir.library.oregonstate.edu/xmlui/bitstream/handle/1957/16166/Stephen_Meyers_PhDthesis.pdf?sequence=1	2010	Assisted Migration	T
Meyers et al.	A molecular phylogeny of Limnanthes (Limnanthaceae) and investigation of an anomalous Limnanthes population from California, USA	Systematic Botany	2010	Assisted Migration	A
**Michalski et al.**	**Evidence for genetic differentiation and divergent selection in an autotetraploid forage grass (Arrhenatherum elatius)**	**Theorethical and applied genetics**	**2010**	**Assisted Migration**	**A**
Mickelsen et al.	Trait specific consequences of fast and slow inbreeding: lessons from captive populations of Drosophila melanogaster	Conservation genetics	2010	Assisted Migration	A
Middleton	Multidisciplinary Approaches to Climate Change Questions	Wetlands	2011	Assisted Migration	B
Middleton and Kleinebecker	The Effects of Climate-Change-Induced	Global Change Ecology and Wetlands	2012	Assisted Migration	A
Millar	Climate Change and Forests of the Future: Managing in the Face of Uncertainty.	Ecological applications	2007	Assisted Migration	A

Millar et al.	Re-Framing Forest and Resource Management Strategies	Mountain Views<br>The Newsletter of the Consortium for Integrated Climate Research in Western Mountains<br>CIRMOUNT	2008	Assisted Migration	P
**Miller et al.**	**Securing the Demographic and Genetic Future of Tuatara through Assisted Colonization**	**Conservation Biology**	**2012**	**Assisted Colonization**	**A**
Miller-Rushing et al.	Conservation consequences of climate change for birds	Moller: Effects of Climate Change on Birds	2010	Managed Translocation	B
Milliken et al.	Amazon vegetation: how much don_t we know and how much does it matter?	Kew Bulletin	2010	Assisted Migration	A
Minteer	Species Conservation, rapid environmental change, and ecological ethics	Nature Education Knowledge	2012	Managed Relocation	P
Minteer	Managed relocation: A case study in ecological ethics	Available at:<br>http://esameetings.allenpress.com/2009/Paper15587.html	2009	Managed Relocation	P
Minteer	Geoengineering and ecological ethics in the anthropocene	BioScience	2012	Manager Relocation, Assisted Migration	A
**Minteer and Collins**	**Move it or lose it? The ecological ethics of relocating species under climate change**	**Ecological applications**	**2010**	**Managed Relocation**	**A**
Miranto et al.	Ex situ conservation of threatened native plants in Finland: analysis of the current status	Endangered species research	2012	Assisted Migration	A
Mitchell and Janzen	Temperature-Dependent Sex	Sexual Development	2010	Assisted Migration	A
**Mitchell et al.**	**Linking Eco-Energetics and Eco-Hydrology to Select Sites for the Assisted Colonization of Australia's Rarest Reptile**	**Biology**	**2012**	**Assisted Colonization**	**P**
**Moir et al.**	**Considering Extinction of Dependent Species during Translocation, Ex Situ Conservation, and Assisted Migration of Threatened Hosts**	**Conservation Biology**	**2012**	**Assisted Migration**	**A**
**Moir et al.**	**A preliminary assessment of changes in plant-dwelling insects when threatened plants are translocated**	**Journal of Insect Conservation**	**2012**	**Assisted Migration, Assisted Colonization**	**A**
Mondoni et al.	Climate warming could shift the timing of seed germination in alpine plants	Annals of Botany	2012	Human Mediated Movement	A
Monks et al.	Determining Success Criteria for Reintroductions of Threatened Long-Lived Plants	Plant reintroduction in a changing climate	2012	Managed Relocation	B
Monteiro-Henriques and Esprito-Santo	Climate change and the outdoor regional living plant collections: an example from mainland Portugal	Biodiversity and Conservation	2011	Managed Migration	A
Monterroso et al.	Potential distribution of two dominant species in the National Park "Nevado de Toluca", central México, current and future climate change scenarios	Jourbal of Natural and Environmental Sciences	2010	Assisted Migration, Assisted Colonization	A
Mooney	The ecosystem-service chain and the biological diversity crisis	Philosophical transactions of the Royal Society	2010	Assisted Colonization	A
Moore	Climate change and animal migration	Environmental law	2011	Assisted Migration	P

Morgera	Far away, so close: an example from mainland Portugal	Climate law	2011	Assisted Migration	P
**Morongiello**	**Climate change and it's implications for Australia's freshwater fish**	**Marine and Freshwater Research**	**2011**	**Managed Translocation**	**A**
Morrison et al.	Proactive Conservation Management	Bioscience	2011	Managed Relocation, Human Assisted Dispersal	A
**Morueta-Holme et al.**	**Climate Change Risks and Conservation Implications for a Threatened Small-Range Mammal Species**	**PLOS ONE**	**2010**	**Assisted Migration**	**A**
Mosca	Adaptive Genetic Diversity of Coniferous Forest Trees of the Italian Alps.	Available at: http://acesap.fem-environment.eu/uploads/14-14_poster_abstract.pdf	2009	Assisted Migration	P
Mosca et al.	The geographical and environmental determinants of genetic diversity for four alpine conifers of the European Alps	Molecular Ecology	2012	Assisted Migration	A
Mueller	Conservation Management Under Climate Change: On Tropical Drought Resistance, Non-native Species Response to Increasing Disturbance, and Assisted Migration	Available at: http://etd.nd.edu/ETD-db/theses/available/etd-11122009-174235/	2009	Assisted Migration	T
**Mueller and Hellmann**	**An Assessment of Invasion Risk from Assisted Migration**	**Conservation Biology**	**2008**	**Assisted Migration**	**A**
Murdock and Flower	Final Technical Report Forest Science Program Project # Y093061 Development and analysis of forest health databases, models, and economic impacts for BC: Spruce bark beetle & spruce; western spruce budworm and Douglas fir	Available at: http://pacificclimate.org/sites/default/files/publications/Murdock.FSPBarkBeetleSpruceBudworm.Apr2009.pdf	2009	Assisted Migration	R
Murphy et al.	Queensland's biodiversity under climate change	Available at: https://publications.csiro.au/rpr/download?pid=csiro:EP115650&dsid=DS4	2012	Assisted Migration, Translocation	R
Murray et al.	‘Raising the bar’: improving the standard and utility of weed and invasive plant research	New Phytologist	2012	Managed Relocation	C
Murthy et al.	Climate change and forests in India: adaptation opportunities and challenges	Mitigation and Adaptation Strategies for Global Change	2011	Assisted Migration	A
Musacchio	The world's matrix of vegetation: Hunting the hidden dimension of landscape sustainability	Landscape and Urban Planning	2011	Assisted Migration	A
Musacchio	The scientific basis for the design of landscape sustainability	Landscape Ecology	2009	Assisted Migration	A
Nakao et al.	Assessing the impact of land use and climate change on the evergreen broad-leaved species of Quercus acuta in Japan	Plant Ecology	2011	Assisted Migration	A
**Nash**	**Western Larch Reforestation in British Columbia: Opportunity and Guidance for Expansion by Assisted Migration to Southern Interior Ecosystems of B.C.**	**Available at: https://circle.ubc.ca/handle/2429/36328**	**2011**	**Assisted Migration**	**T**

Neale	Genetic Considerations in Rare Plant Reintroduction: Practical Applications (or How Are We Doing?)	Plant reintroduction in a changing climate	2012	Managed Relocation	B
Neophytou	A study of genetic differentiation and hybridization among oak species with divergent ecological and evolutionary profiles	Available at: http://deposit.ddb.de/cgi-bin/dokserv?idn=1010642391&dok_var=d1&dok_ext=pdf&filename=1010642391.pdf	2010	Assisted Migration	T
New	Conserving narrow range endemic insects in the face of climate change: options for some Australian butterflies	Journal of insect conservation	2008	Assisted Colonisation	A
Nichols and Neale	Association genetics, population genomics, and conservation	DeWoody et al.: Molecular Approaches in Natural Resource Conservation and Management	2010	Assisted Migration	B
Nillesen and van Ierland	Climate Change C	Available at: http://www.rivm.nl/bibliotheek/rapporten/500102003.pdf	2006	Artificial Translocation	R
Nitschke and Innes	Integrating climate change into forest management in South-Central British Columbia: An assessment of landscape vulnerability and development of a climate-smart framework	Forest Ecology and Management	2008	Human Assisted Migration	A
Noss	Between the devil and the deep blue sea: Florida’s unenviable position with respect to sea level rise	Climatic Change	2011	Assisted Colonization	A
Notaro et al.	Projected vegetation changes for the American Southwest: combined dynamic modeling and bioclimatic-envelope approach	Ecological applications	2012	Assisted Migration	A
Notaro et al.	Vegetation and land carbon projections for Wisconsin, USA, in the 21st century	Climate Research	2012	Assisted Migration	A
Oberle and Schaal	Responses to historical climate change identify contemporary threats to diversity in Dodecatheon	Proceedings of the National Academy of Sciences	2011	Assisted Migration	A
O'brien et al.	Adding climate change to the mix: using climate futures in conservation planning for Oregon's oak-dominated habitats	Available at: http://scholarsarchive.library.oregonstate.edu/xmlui/bitstream/handle/1957/27967/062811_workshop_summary.pdf?sequence=1	2011	Assisted Migration	R
Odenbaugh	Refounding Environmental Ethics: Pragmatism, Principle, and Practice. (bookreview)	Bioscience	2012	Assisted Migration	P
**Ogawa-Onishi et al.**	**Assessing the potential impacts of climate change and their conservation implications in Japan: A case study of conifers**	**Biological conservation**	**2010**	**Assisted Migration**	**A**
**Olden et al.**	**Challenges and Opportunities in Implementing Managed Relocation for Conservation of Freshwater Species**	**Conservation biology**	**2011**	**Managed Relocation**	**A**
Olden et al.	Conservation biogeography of freshwater fishes: recent progress and future challenges	Diversity and Distributions	2010	Assisted Migration, Translocation	A
**Olivera-Santos and Fernandez**	**Reintroduction and Refaunation: Response to Seddon et al.**	**Conservation Biology**	**2011**	**Assisted Colonization**	**A**

Olmsted	Climate Surfing: A Conceptual Guide to Drafting Conservation Easements in the Age of Global Warming.	St. John's Journal of Legal Commentary	2008	Assisted Migration	A
Olmstedt	Climate Surfing: A Conceptual Guide to Drafting	Journal of Civil Rights and Economic development	2012	Assisted Migration	A
Olmstedt	Carbon Dieting: Latent Ancillary Rights to Carbon Offsets in Conservation Easments.	Journal of Land, Resources & Environmental Law	2009	Assisted Migration	P
Olson et al.	The adaptive potential of Populus balsamifera L. to phenology requirements in a warmer global climate	Molecular Ecology	2012	Assisted Migration	A
Olson et al.	Managing for Climate Change within Protected Area Landscapes	Natural Areas journal	2009	Assisted Migration	A
O'Neill et al.	Assisted migration to address climate change in BC: recommendations for interim seed transfer standards	Available at: http://bcwildfire.ca/ftp/HTI/external/!publish/Interim_Measures/Report/Assisted%20migration%20Technical%20Report_13.doc	2008	Assisted Migration	R
**O'Neill et al.**	**Accounting for population variation improves estimates of the impact of climate change on species' growth and distribution**	**Journal of Applied Ecology**	**2008**	**Assisted Migration**	**A**
Ouedraogo et al.	Evidence for important genetic differentiation between provenances of Parkia biglobosa from the Sudano-Sahelian zone of West Africa	Agroforestry Systems	2012	Assisted Migration	A
Overpeck et al.	Terrestrial Biosphere Dynamics in the Climate	Alverson et al: Paleoclimate, Global Change, and the Future	2003	Assisted Migration	B
Owley	Conservation Easements at the Climate Change Crossroads	Law & Contemporary Problems	2011	Assisted Migration	A
Park and Talbot	Assisted migration: uncertainty, risk and opportunity	Forestry Chronicle	2012	Assisted Migration	P
Parker and Hellmann.	An experimental test of northern range limitation in two contrasting butterflies	Available at: http://eco.confex.com/eco/2008/techprogram/P12752.HTM	2008	Assisted Migration	P
**Parker et al.**	**Opportunities for Nonnative Ecological Replacements in Ecosystem Restoration**	**Restoration ecology**	**2010**	**Assisted Translocation**	**A**
Parks	The future of spring bud burst: looking at the possibilities	Science findings	2010	Assisted Migration	P
Parmesan	Species' range shifts: Global trends and potential for adaptation and mitigation through ecosystem restoration	Available at: http://eco.confex.com/eco/2010/techprogram/P22029.HTM	2010	Human Assisted Translocation	P
**Pautasso et al.**	**Impacts of climate change on plant diseases—opinions and trends**	**European Journal of Plant Pathology**	**2012**	**Assisted Migration**	**A**
Pautasso et al.	Plant health and global change – some implications for landscape management	Biological reviews	2010	Assisted Migration	A
Pearce	Achieving Climate Change Adaptation in West Kootenay	Available at: http://kootenayresilience.homestead.com/Report8_BarriersOpportunities_Final.pdf	2012	Assisted Migration	R
Pearlstine at al.	A review of the ecological consequences and management	Journal of the North American Benthological Society	2010	Assisted Colonization	A

Pearson and Dawson	Long-distance plant dispersal and habitat fragmentation	Biological conservation	2005	Human Assisted Migration	A
**Pedlar et al.**	**Placing Forestry in the Assisted Migration Debate**	**BioScience**	**2012**	**Species Rescue Assisted Migration, Assisted Migration, Forestry Assisted Migration, Assisted Population Migration**	**A**
Pedlar et al.	The implementation of assisted migration in Canadian forests	Forestry Chronicle	2011	Assisted Migration	P
Pereira	Global Biodiversity Change: The Bad, the Good, and the Unknown	Annual Review of Environment and Resources	2012	Assisted Migration	A
Perez et al.	What is wrong with current translocations? A review and a decision-making proposal	Frontiers in Ecology and the Environment	2012	Assisted Colonization	A
Perry	World Heritage hot spots: a global model identifies the 16 natural heritage properties on the World Heritage List most at risk from climate change	International Journal of Heritage Studies	2011	Assisted Migration, Assisted Colonization, Assisted Translocation	A
Peterson et al.	Responding to Climate Change on National Forests: A Guidebook for Developing Adaptation Options	Available at: http://www.fs.fed.us/psw/publications/millar/Peterson%20et%20al.%20(Adaptation%20Guidebook)%20(Feb.%202011).pdf	2011	Assisted Migration	R
Petit et al.	Forests of the Past	SCIENCE	2008	Assisted Migration	A
Pickles et al.	Ectomycorrhizas and climate change	Fungal Ecology	2012	Assisted Migration, Facilitated Migration	A
Pike	What Is Local? An Introduction to Genetics and Plant Selection in the Urban Context	Urban habitats	2008	Assisted Migration	A
Pinchot	Forest Service Strategic Framework.	Available at: http://myhoodisyourhood.org/content/599/2009_strategic-framework-climate-change-1-0.pdf	2008	Assisted Migration	R
Pinnell	Climate Change and Forest Health: Impacts to	Available at: http://www.kootenayresilience.org/Report6_ForestHealth_Final.pdf	2012	Assisted Migration	R
Pinsky et al.	Dispersal provided resilience to range collapse in a marine mammal: insights from the past to inform conservation biology	Molecular ecology	2010	Human Assisted Relocation, Assisted Migration	A
Poff et al.	Climate Change and Freshwater Fauna Extinction Risk	Saving a Million species	2012	Assisted Dispersal	B
Pojar	A New Climate for Conservation	Available at: http://w.y2y.net/data/1/rec_docs/726_NewClimate_Report_reduced.pdf	2010	Facilitated Migration	R
Ponce-Reyes et al.	Vulnerability of cloud forest reserves in Mexico to climate change	Nature Climate Change	2012	Assisted Colonization	A
Pool	Move it or lose it	Engineering &Technology	2012	Assisted Migration	P
Popescu and Hunter	Assisted colonization of Wildfire Species at risk from climate chnage	Wildfire Conservarvation in a changing climate	2012	Assisted Colonization	B

Porter et al.	Interactive effects of anthropogenic nitrogen enrichment and climate change on terrestrial and aquatic biodiversity	Biogeochemistry	2012	Assisted Migration	A
**Potter and Hargrove**	**Determining suitable locations for seed transfer under climate change: a global quantitative method**	**New Forests**	**2012**	**Plant Refuge Translocations, Human Assisted Migration**	**A**
Poulter et al.	Sea-level rise research and dialogue in North Carolina	Ocean and coastal management	2009	Assisted Species Dispersal	A
Powledge	The evolving Role of Botanical Gardens	BioScience	2011	Assisted Migration	A
Prasad	Melanie Lenart: Life in the hothouse: how a living planet survives climate change (book review)	Landscape ecology	2012	Assisted Migration	P
Prasad	Assessing potential species distributions under future climates using a multi-stage modelling approach	Available at: http://eco.confex.com/eco/2010/techprogram/P21812.HTM	2010	Assisted Migration	P
**Price**	**Assisted Colonization: Move Ahead with Models**	**Science**	**2010**	**Assisted Colonization**	**A**
Primack et al.	The role of botanical gardens in climate change research	New Phytologist	2009	Assisted Migration	A
Prior	Novel Community Interactions Following Species' Range Expansions.	Available at: http://etd.nd.edu/ETD-db/theses/available/etd-07182011-144509/unrestricted/PriorKM072011D.pdf	2011	Managed Relocation	T
Pritchard et al.	Bring the captive closer to the wild: redefining the role of ex situ conservation	Oryx	2012	Assisted Migration	P
Prober et al.	Facilitating adaptation of biodiversity to climate change: a conceptual framework applied to the world’s largest Mediterranean-climate woodland	Climatic Change	2012	Translocation	A
Prunier et al.	Parallel and lineage-specific molecular adaptation to climate in boreal black spruce	Molecular Ecology	2012	Assisted Migration	A
Prunier et al.	Scanning the genome for gene SNPs related to climate adaptation and estimating selection at the molecular level in boreal black spruce	Molecular Ecology	2011	Assisted Migration	A
Puettmann	Silvicultural Challenges and Options in the Context of Global Change: “Simple“ Fixes and Opportunities for New Management Approaches	Journal of Forestry	2011	Assisted Migration	A
Puric-Mladenovic et al.	An analysis of the vulnerabilities of Terrestrial Ecosystems/Vegetation Cover to climate change in the Lake Simcoe watershed	The Ontario Centre for Climate Impacts and Adaptation Resources , report	2011	Assisted Migration	R
**Qie**	**Assisted Dispersal of Tropical Dung Beetles.**	**The Raffles Bulletin of Zoology**	**2012**	**Assisted Dispersal**	**A**
Qualtiere	Impacts of Climate Change on the Western Canadian Southern Boreal Forest Fringe	Available at: http://www.parc.ca/rac/fileManagement/upload/12855-3E11%20Impacts%20of%20CC%20on%20theWCanadian%20S%20Boreal%20Fringe%20%28SRC%29.pdf	2011	Assisted Migration	R

Qualtiere	Variation in germination response to temperature among collections of three conifers from the mixed wood forest	Available at: http://library2.usask.ca/theses/available/etd-05152008-134225/unrestricted/Qualtiere.pdf	2008	Assisted Migration	T
Rahel	Managing Aquatic Species of Conservation Concern in the Face of Climate Change and Invasive Species	Conservation Biology	2008	Assisted Migration	A
Ramage et al.	Forest transformation resulting from an exotic pathogen: regeneration and tanoak mortality in coast redwood stands affected by sudden oak death	Candian Journal of Forest Research	2011	Assisted Migration	P
Ravenscroft	Modeling forest restoration under climate change at multiple spatial scales	Available at: http://eco.confex.com/eco/2008/techprogram/P9555.HTM	2008	Assisted Migration	P
Ravenscroft et al.	Forest restoration in a mixed-ownership landscape under climate change	Ecological applications	2010	Assisted Migration	A
Readfern	Preparing to adapt to unavoidable climate change	ECOS	2010	Assisted Migration	P
Reddy	Tourism and climate change risks: opportunities and constraints in South Africa	Available at: http://wiredspace.wits.ac.za/handle/10539/11392	2012	Assisted Migration	T
Redford et al.	What Does It Mean to Successfully Conserve a (Vertebrate) Species?	Bioscience	2011	Assisted Migration	A
Reed et al	Inbreeding–stress interactions: evolutionary and conservation consequences	Annals of the New York academy of Sciences	2012	Assisted Migration	A
**Regan et al.**	**Evaluation of assisted colonization strategies under global change for a rare, fire-dependent plant**	**Global Change Biology**	**2012**	**Assisted Colonization**	**A**
Reichard et al.	Is Managed Relocation of Rare Plants Another Pathway for Biological Invasions?	Plant reintroduction in a changing climate	2012	Managed Relocation	B
**Ren et al.**	**Reintroduction of Tigridiopalma magnifica, a rare and Critically Endangered herb endemic to China**	**Fauna & Flora International, Oryx,**	**2012**	**Human Assisted Migration, Human Assisted Colonization**	**A**
Renton	Dynamic modelling to predict the likelihood of plant species persisting in fragmented landscapes in the face of climate change	Available at: http://www.mssanz.org.au/modsim2011/E11/renton.pdf	2011	Assisted Migration	P
**Renton et al.**	**Habitat restoration will help some functional plant types persist under climate change in fragmented landscapes**	**Global Change Biology (**	**2012**	**Assisted Migration**	**A**
**Riccardi and Simberloff**	**Assisted colonization is not a viable conservation strategy**	**Trends in Ecology and Evolution**	**2009**	**Assisted Colonization**	**A**
**Riccardi and Simberloff**	**Assisted colonization: good intentions and dubious risk assessment**	**Trends in Ecology and Evolution**	**2009**	**Assisted Colonization**	**A**
Richardson	Fifty years of Invasion Ecology: The Legacy of Charles Elton	Richardson: Fifty years of Invasion Ecology: The Legacy of Charles Elton	2011	Assisted Migration, Managed Relocation	B
Richardson	Multidimensional evaluation of managed relocation	Available at: http://eco.confex.com/eco/2009/techprogram/P15595.HTM	2009	Managed Relocation	P

Richardson	Conservation biogeography: what's hot and what's not?	Diversity and Distributions	2012	Managed Translocation	A
Richardson et al.	Australian acacias take on the world: Lessons for management from a global translocation experiment	Available at: http://eco.confex.com/eco/2011/preliminaryprogram/abstract_27330.htm	2011	Managed Relocation	P
**Richardson et al.**	**Multidimensional evaluation of manged relocation**	**Proceedings of the National Academy of Sciences**	**2009**	**Managed Relocation**	**A**
Richmond et al.	Is the Climate Right for Pleistocene Rewilding? Using Species Distribution Models to Extrapolate Climatic Suitability for Mammals across Continents	PLOS ONE	2010	Assisted Migration	A
**Richter et al.**	**Phenotypic plasticity facilitates resistance to climate change in a highly variable environment**	**Oecologia**	**2012**	**Assisted Migration**	**A**
Riegl et al.	Present Limits to Heat-Adaptability in Corals and Population-Level Responses to Climate Extremes	PLOS ONE	2011	Assisted Migration	A
Rieman and Isaak	Climate Change, Aquatic Ecosystems, and Fishes in the Rocky Mountain West: Implications and Alternatives for Management	Available at: http://www.regions.noaa.gov/western/pdfs/library/2010_USFW_Climate_Change_Aquatic_Ecosystems_and_Fishes.pdf	2010	Human Assisted Migration, Assisted Migration, Facilitated Dispersal	R
**Rija et al.**	**The aftermath of environmental disturbance on the critically endangered Coffea kihansiensis in the Southern Udzungwa Mountains, Tanzania**	**Tropical conservation science**	**2011**	**Assisted Migration**	**A**
Rija et al.	Reclaiming the lost worls: Kihansi spray toad re-introduction in Tanzania	Sooare 2010: Global Re-introduction Perspectives: Additional Case Studies from Around the ..	2010	Benign Introduction	R
Robbins	First Day Jitters: Coming to Terms with Yesterday's Passing and Stepping Forward into a New Day of Species Protection in the Post-Climate-Change Era	ABA Section of Environment, Energy, and Resources, 20th Section Fall Meeting, Austin, TX, October 10-13, 2012. Available at SSRN: Available at: http://ssrn.com/abstract=2147726 or Available at: http://dx.doi.org/10.2139/ssrn.2147726	2012	Assisted Migration	C
Roberts and Hamann	Method selection for species distribution modelling: are temporally or spatially independent evaluations necessary?	Ecography	2012	Assisted Migration	A
Robinson	Patterns of Ecological Performance and Aquatic Insect Diversity in High Quality Protected Area Networks	Available at: http://trace.tennessee.edu/utk_graddiss/1342/	2012	Assisted Migration	T
Roderick et al.	Evolution and biological control	Evolutionary applications	2012	Assisted Migration	A
Roe et al.	No place like home: an experimental comparison of reintroduction strategies using snakes	Journal of Applied Ecology	2010	Assisted Colonization	A
Rohde et al.	Temperature signals contribute to the timing of photoperiodic growth cessation and bud set in poplar	Tree physiology	2011	Assisted Migration	A
Roncal et al.	Testing appropriate habitat outside of historic range: The case of Amorpha herbacea var. crenulata (Fabaceae)	Journal for Nature Conservation	2012	Translocation	A

Rondini and Boitani	Mind the map: trips and pitfalls in making and reading maps of carnivore distribution	Carnivore Ecology and Conservation: A handbook of Techniques	2012	Assisted Migration	B
Rose and Burton	Persistent climate corridors: The identification of climate refugia in British Columbia’s Central Interior for the selection of candidate areas for conservation	BC journal of ecosystem and management	2011	Assisted Migration	A
Rose and Burton	Using bioclimatic envelopes to identify temporal corridors in support of conservation planning in a changing climate	Forest ecology and management	2009	Assisted Migration	A
Ross et al.	Disturbance and the rising tide: the challenge of biodiversity management on low-island ecosystems	Frontiers in Ecology and the Environment	2009	Assisted Migration	A
Rossetto and Rymer	Applications of Molecular markers in Plant Conservation	Molecular markers in plants	2012	Assisted Migration	N
Rowland	Environmental values and assisted colonization: A reply to Sandler	Available at: http://eco.confex.com/eco/2011/preliminaryprogram/abstract_31886.htm	2011	Assisted Colonization	P
Rudd	Scientists’ Opinions on the Global Status and Management of Biological Diversity	Conservation Biology	2011	Assisted Migration	A
**Ruhl**	**Assisted Colonization: Facilitate Migration First**	**Science**	**2010**	**Assisted Colonization**	**A**
Ruhl	Adapting the Endangered Species Act to climate change	Boston University law review	2008	Assisted Migration	A
Ruhl andSalzman	Gaming the Past: The Theory and Practice of Historic Baselines in the Administrative State	Vanderbilt Law Review	2011	Assisted Migration	A
Rull	Sustainability, capitalism and evolution	EMBO reports	2011	Assisted Migration	P
Rull et al.	Conservation of the Unique Neotropical Vascular Flora of the Guayana Highlands in the Face	Conservation Biology	2009	Assisted Migration	A
Rumbaitis del Rio	The Role of Ecosystems in Building Climate Change Resilience and Reducing Greenhouse Gases	Integrating Ecology and Poverty Reduction	2012	Assisted Migration	B
Ruthrof et al.	The efficacy of soil ameliorants to improve early establishment in trees and shrubs in degraded 'Eucalyptus gomphocephala' woodlands	Pacific Conservation biology	2012	Assisted Colonization, Assisted Migration	A
**Saentz-Romero**	**Altitudinal Genetic Variation Among Pinus patula Ppopulations from Oaxaca, Mexico, in Growth Chambers Simulating Global Warming Temperatures.**	**Agrociencia**	**2011**	**Assisted Migration**	**A**
Saenz-Romero et al.	Latitudinal Genetic Variation among Pinus pseudostrobus Populations from Michoacán, México. Two Location Shadehouse Test Results.	Revista Fitotecnia Mexican	2012	Assisted Migration	A
**Saenz-Romero et al.**	**SPLINE MODELS OF CONTEMPORARY, 2030, 2060 AND 2090 CLIMATES FOR MICHOACÁN STATE, MÉXICO. IMPACTS ON THE VEGETATION**	**Revista Fitotecnia Mexican**	**2012**	**Assisted Migration**	**A**
**Saenz-Romero et al.**	**Abies religiosa habitat prediction in climatic change scenarios and implications for monarch butterfly conservation in Mexico**	**Forest Ecology and Management**	**2012**	**Assisted Migration**	**A**

Saenz-Romero et al.	Spline models of contemporary, 2030, 2060 and 2090 climates for Mexico and their use in understanding climate-change impacts on the vegetation	Climatic change	2010	Assisted Migration	A
Safford et al.	Climate change and the relevance of historical forest conditions	Available at: http://www.fs.fed.us/psw/publications/documents/psw_gtr237/psw_gtr237_023.pdf	2012	Human Assisted Dispersal	R
Safford et al.	The growing importance of the past in managing ecosystems of the future	Historical Environmental variation in conservation and natural resource…	2012	Managed Relocation	B
**Safont et al.**	**Use of Environmental Impact Assessment (EIA) tools to set priorities and optimize strategies in biodiversity conservation**	**Biological Conservation**	**2012**	**Managed Relocation**	**A**
Sahasrabudhe and Motter	Rescuing ecosystems from extinction cascades through compensatory perturbations	Nature communications	2011	Assisted Migration	A
Sainsbury and Vaughan-Higgins	Analyzing Disease Risks Associated with Translocations	Conservation Biology	2012	Translocation	A
Sandler	The ethics of species: an introduction	The ethics of species: an introduction	2012	Assisted Colonization	B
**Sandler**	**The Value of Species and the Ethical Foundations of Assisted Colonization**	**Conservation Biology**	**2009**	**Assisted Colonization**	**A**
Sauychin	Saskatchewan's Natural Capital in a Changing Climate	Available at: http://www.parc.ca/pdf/research_publications/pp2009-02_web.pdf	2009	Assisted Migration	R
Sawden	Management of British Columbia's Forests in Response to Climate Change	Available at: https://circle.ubc.ca/bitstream/handle/2429/16114/SawdenAndrew_FRST_497_Graduating_Essay_2008%20.pdf?sequence=1	2009	Assisted Migration	T
Savolainen et al.	Adaptive potential of northernmost tree populations to climate change, with emphasis on Scots pine (Pinus sylvestris L.)	Journal of heredity	2011	Assisted Migration	A
Sax et al	Ecological and evolutionary insights from species invasions	Trends in Ecology & Evolution	2007	Assisted Migration	A
**Sax et al.**	**Managed relocation: A nuanced evaluation is needed**	**Trends in Ecology and Evolution**	**2009**	**Managed Relocation**	**A**
**Schlaepfer et al.**	**Assisted colonization: evaluating contrasting management actions (and values) in the face of uncertainty**	**Trends in Ecology and Evolution**	**2009**	**Assisted Colonization**	**A**
Schlaepfer et al.	The Potential Conservation Value of Non-Native Species	Conservation Biology	2011	Assisted Migration	A
Schmid	Reviews and notices of publications	Taxon	2012	Managed Relocation	P
Schneider	Uncertainty/limits to adaptation/adapting to +4C: a transcript	Climate Adaptation Futures	2012	Managed Relocation	B
Schoettle et al.	Integrating Regeneration, Genetic Resistance, and Timing of Intervention for the Long-Term Sustainability of Ecosystems Challenged by Non-Native Pests – a Novel Proactive Approach	Available at: http://www.fs.fed.us/rm/pubs_other/rmrs_2012_schoettle_a002.pdf	2012	Assisted Migration	R

Schoettle et al.	Geographic patterns of genetic variation and population structure in Pinus aristata, Rocky Mountain bristlecone pine	Canadian Journal of Forest Research	2012	Assisted Migration	A
Schramm and Fishman	Legal Frameworks for Adaptive Natural Resource	The Georgetown International Environmental Law Review	2010	Assisted Migration	A
Schuler and Orrock	The maladaptive significance of maternal effects	Evoutionary ecology	2012	Assisted Dispersal	A
Schulman and Lehvävirta	Botanic gardens in the age of climate change	Biodiversity and Conservation	2011	Assisted Migration	A
Schulze et al.	Large-scale bioenergy from additional harvest of forest biomass is neither sustainable nor greenhouse gas neutral	Bioenergy	2012	Assisted Migration	A
Schurr et al.	How to understand species' niches and range dynamics: a demographic research agenda for biogeography	Journal of Biogeography	2012	Assisted Migration	A
Schwartz	Strategies for conserving plants through (re)introduction (book review)	Landscape ecology	2012	Managed Relocation	P
Schwartz	Evaluating assisted migration and the potential mismatches between species distributions and climate	Available at: http://eco.confex.com/eco/2009/techprogram/P15573.HTM	2009	Assisted Migration	P
**Schwartz**	**The precautionary principle in managed relocation is misguided advice**	**Trends in Ecology and Evolution**	**2009**	**Managed Relocation**	**A**
**Schwartz et al.**	**Managed Relocation Integrating the Scientific, Regulatory, and Ethical Challenges**	**BioScience**	**2012**	**Managed Relocation**	**A**
Schwartz et al.	Predicting extinctions as a result of climate change	Ecology	2006	Assisted Migration	A
Science Daily	Should We Move Species to Save them from Climate Change	Available at: http://www.sciencedaily.com/releases/2008/07/080717140445.htm	2008	Assisted Migration	P
Seabrook et al.	Restore, repair or reinvent: Options for sustainable landscapes in a changing climate	Landscape and Urban planning	2011	Assisted Translocation, Assisted colonisation	A
**Seddon**	**From Reintroduction to Assisted Colonization: Moving along the Conservation Translocation Spectrum**	**Restoration Ecology**	**2010**	**Assisted Colonization**	**A**
**Seddon**	**Guidelines for Subspecific Substitutions in Wildlife Restoration Projects**	**Conservation ecology**	**1999**	**Benign Introduction**	**A**
Seddon et al.	Animal translocations: What are they and why do we do them?	Reintroduction Biology: Integrating Science and Mangement	2012	Assisted Colonization	B
**Seddon et al.**	**Frankenstein Ecosystems and 21st Century Conservation Agendas: Reply to Oliveira-Santos and Fernandez**	**Conservation Biology**	**2011**	**Assisted Colonization**	**A**
**Seddon et al.**	**The risks of Assisted Colonization**	**Conservation Biology**	**2009**	**Assisted Colonization**	**A**
Seidl	Finding Higher Ground - Adaptation in the Age of Warming	Finding Higher Ground - Adaptation in the Age of Warming	2011	Assisted Migration	B
Sekercioglu et al	The effects of climate change on tropical birds	Biological conservation	2012	Assisted Migration	A

Selig	Radial-Growth Forecasting and the Implications for Planning and Management in the Grand River Watershed of Ontario, Canada	Available at: https://libdspace.uwaterloo.ca/bitstream/10012/4331/1/nselig1.pdf	2009	Assisted Migration	T
Sexton et al.	Incorporating sociocultural adaptive capacity in conservation hotspot assessments	Diversity and Distributions	2010	Managed Relocation	A
Sgro et al.	Building evolutionary resilience for conserving biodiversity under climate change	Evolutionary applications	2011	Assisted Migration	A
Shashidhar and Kumar	Effect of Climate Change on orhids and their conservation strategies	The Indian Forester	2009	Assisted Migration	P
Shaw and Etterson	Rapid climate change and the rate of adaptation: insight from experimental quantitative genetics	New Phytologist	2012	Human Assistance of Dispersal	A
Sheean et al.	An assessment of scientific approaches towards species relocations in Australia	Austral Ecology	2012	Assisted Colonization	A
Sheikh et al.	CRS Report for Congress: Global Climate Change and Wildlife	Available at: http://assets.opencrs.com/rpts/RS22597_20080515.pdf	2008	Assisted Migration	R
Sheppard	A new course in visualizing climate change	Branchlines	2011	Assisted Migration	P
Shier and swaisgood	Fitness Costs of Neighborhood Disruption in Translocations of a Solitary Mammal	Conservation Biology	2012	Assisted Migration	A
**Shirey and Lamberti**	**Assisted colonization under the U.S. Endangered Species Act**	**Conservation Letters**	**2010**	**Assisted Colonization**	**A**
Shirey and Lamberti	Regulate trade in rare plants	Nature	2011	Assisted Colonization	A
Shultz et al.	Conservation of Prairie-Oak Butterflies in Oregon, Washington, and British Columbia	Northwest Science	2011	Assisted Colonization	A
Siddiqui et al.	Forest ecosystem climate change impact assessment	Climate Research	1999	Artificial Translocation	A
Siebert	Make Way for the Movable Beast.	Oneearth	2009	Assisted Colonization	P
Simard	Mycorrhizal networks and complex systems: Contributions	Canadian Journal of Soil Science	2009	Assisted Migration	A
Sinclair	RD&E strategy for the forest and wood products sector	Available at: http://www.daff.gov.au/__data/assets/word_doc/0010/1770751/forest.doc	2010	Assisted Migration	R
Singh et al.	Rapid warming in the Himalayas: Ecosystem responses and development options	Climate and development	2010	Assisted Migration	P
Skou et al.	Tracing the introduction history of a potentially invasive ornamental shrub: variation in frost hardiness and climate change	Nordic Journal of Botany	2012	Assisted Migration	A
Smith	Assessing the Viability of Managed Relocation as a Conservation Strategy	Available at: http://www.iccs.org.uk/thesis/consci/msc10-smith,matthew.pdf	2010	Managed Relocation	T
Smith et al.	A European perspective for developing	Renewable Agriculture and Food Systems	2012	Assisted Migration	A
Sooare	Global re-introduction perspectives: re-introduction case-studies from around the globe	Available at: https://cmsdata.iucn.org/downloads/rsg_book_2013.pdf	2008	Conservation Introduction, Benign Introduction	B

Soolanayakanahally	Latitudinal Gradients in Adaptive Traits of Populus.	Available at: https://circle-prod.library.ubc.ca/bitstream/handle/2429/30232/ubc_2011_spring_soolanayakanahally_raju.pdf?sequence=1	2010	Assisted Migration	T
Soto-Correa et al.	GENETIC VARIATION BETWEEN Lupinus elegans Kunth PROVENANCES, ALTITUDINAL SEED ZONING AND ASSISTED MIGRATION	Agrociencia	2012	Assisted Migration	A
Souther and McGraw	Evidence of Local Adaptation in the Demographic Response of American Ginseng to Interannual Temperature Variation	Conservation Biology	2011	Assisted Relocation	A
Souther et al.	Experimental test for adaptive differentiation of ginseng populations reveals complex response to temperature	Annals of Botany	2012	Human Assisted Relocation	A
Southgate et al.	Ascesis and assisted migration responses to the effects of climate change on animal species	European Journal of Science and Theology	2008	Assisted Migration	P
Spear and Chown	The extent and impacts of ungulate translocations: South Africa in a global context	Biological Conservation	2009	Assisted Migration	A
Spies et al.	Climate change adaptation strategies for federal forests of the Pacific Northwest, USA: ecological, policy, and socio-economic perspectives	Landscape Ecology	2010	Assisted Migration	A
Spratte-Lennington	The consequences of Climate-Induced Range Expansions	Available at: http://www.transy.edu/holleian_society/journal/2009/Kelly_Spratte_Lennington.pdf	2009	Assisted Migration, Human Assisted Migration	T
**St Clair and Howe**	**Startegies for conserving forest genetic resources in the face of climate change**	**Turkish Journal of Botany**	**2011**	**Assisted Colonization**	**A**
**St Clair and Howe**	**Ensuring that forests are adapted to future climates**	**Northwest Woodlands**	**2010**	**Assisted Migration**	**P**
Standish and Hobbs	Improving city life - restoration as a component of urban renewal and adaptation to climate change	Available at: http://eco.confex.com/eco/2010/techprogram/P22668.HTM	2010	Assisted Migration	P
**Stanley-Price**	**IUCN Species Survival Commission: the Reintroduction and Invasive Species Specialist Groups' Task Force for Moving Species for Conservation Purposes.**	**Oryx**	**2010**	**Assisted Migration**	**A**
Staudinger et al.	Impacts of Climate Change on Biodiversity, Ecosystems, and Ecosystem Services	Available at: http://crownmanagers.org/storage/Biodiversity-Ecosystems-and-Ecosystem-Services-Technical-Input.pdf	2012	Assisted Migration	R
Steenberg et al.	Exploring adaption to vlimate change in the forests of Central Nova Scotia, Canada	Forest ecology and management	2011	Assisted Migration	A
Steenberg et al.	Indicators of Sustainable Forest.	Available at: http://bcwildfire.ca/ftp/HFP/external/!publish/Web/FFESC/reports/CI-SFM-ClimateChange_FinalReport_111017.pdf	2011	Assisted Migration	R

Steinhoff	Naturalness and Biodiversity: Why Natural Conditions Should Be Maintained within Protected Areas	william and mary environmental law and policy review	2012	Assisted Migration	A
Ste-Marie	Chasing Climate Change–Exploring the Option of Assisted Migration	Forestry Chronicle	2011	Assisted Migration	P
Ste-Marie et al.	Assisted migration: introduction to a multifaceted concept	Forestry Chronicle	2011	Assisted Migration	P
Stephens et al.	Operational approaches to managingforests of the future in Mediterranean regions within a context of changing climates	Environmental Research Letters	2010	Assisted Migration	A
Sthapit et al.	Tropical Fruit Tree Genetic Resources	Available at: http://www.ecoagriculture.org/documents/files/doc_420.pdf#page=99	2012	Assisted Migration	R
Stokes and Howden	Looking forward	Stokes: Adapting Agriculture to Climate Change: Preparing Australian agriculture...	2010	Assisted Migration	B
Stone	Home, home Outside the range?	Science	2010	Assisted Colonization	P
Stout	Silvicultural choices in combating climate change	Available at: http://eco.confex.com/eco/2010/techprogram/P21824.HTM	2010	Facitated Migration	P
Strecker et al.	Defining conservation priorities for freshwater fishes according to taxonomic, functional, and phylogenetic diversity	Ecological applications	2011	Assisted Colonization	A
Strifling	Irreversible? An Ecosystem-Based Approach to Slowing the Synergistic Effects of Invasive Species and Climate Change	Duke Environmental Law & Policy Forum	2011	Assisted migartion	P
Sturrock	Climate change and forest diseases	Forest systems	2012	Assisted Migration	A
Sturrock et al.	Climate change and forest diseases	Plant Pathology	2011	Facilitated Migration, Assisted Migration	A
Sunderman et al.	River restoration success depends on the species pool of the immediate surroundings	Ecological applications	2011	Assisted migartion	A
Sutherland	A horizon scan of global conservation issues for 2010	Trends in Ecology & Evolution	2010	Assisted Colonisation	A
**Swarts and Dixon**	**Terrestrial orchid conservation in the age of extinction**	**Annals of Botany**	**2009**	**Assisted Migration**	**A**
Svenning et al.	Big moving day for biodiversity? A macroecological assessment of the scope for assisted colonization as a conservation strategy under global warming	IOP Conference Series: Earth and Environmental Science	2009	Assisted Colonization	P
Swift	Decision Support Framework: A Mental Model for Integrating the Environmental and Human Dimensions into Decision Making Related to Changes in Natural Disturbance Events	Journal of Environmental Monitoring	2012	Assisted Migration	P
Swift	2010 Winter SISCO Workshop – Climate Change Panel Discussion Searching for sustainability in forest management: Is good silviculture the key?	BC journal of ecosystem and management	2011	Assisted Migration	P

Swift	Searching for sustainability in forest management: Is good silviculture the key?	LINK News	2010	Assisted Migration	P
Swift	2008 Winter SISCO: The resilient forest	Forrex	2008	Assisted Migration	P
Swift and Ran	Successional Responses to Natural Disturbance, Forest Management and Climate Change in British Columbia Forests	Journal of Ecosystems and management	2012	Assisted Migration	P
Tam	Understanding preferences for climate change adaptation for protected areas: the psychology of individual risk perceptions	Available at: https://circle.ubc.ca/handle/2429/28123	2010	Assisted Colonization	T
Tanaka et al.	Predicting the impact of climate change on potential habitats of fir (Abies) species in Japan and on the East Asian continent	Procedia Environmental Sciences	2012	Assisted Migration	A
Tanaka et al.	SRM Center for Professional Education and Development: Wildfires and Invasive Plants in American Deserts	Rangelands	2009	Assisted Migration	R
Taylor and Hamilton	Impact of climatic change on tropical forests in Africa: Implications for protected area planning and management	IUCN; Impacts of climate change on Ecosystems and Species: Implications for Protected Areas	1994	Human Assisted Dispersal, Assisted Dispersal	B
Thiel et al.	Uniform drought and warming responses in Pinus nigra provenances despite specific overall performances	Forest Ecology and Management	2012	Controlled Introduction, Assisted Colonization, Assisted Migration	A
Thomas	Shelter from the storm	New Scientist	2011	Assisted colonisation	P
**Thomas**	**Translocation of species, climate change, and the end of trying to recreate past ecological communities**	**Trends in Ecology and Evolution**	**2011**	**Assisted colonisation**	**A**
Thomas et al.	A framework for assessing threats and benefits to species responding to climate change	Methods in Ecology and Evolution	2011	Translocation	A
Thompson	The Need for (and Obstacles to) Regional Collective Action in Climate Adaptation	Available at: http://165.124.73.46/searlecenter/papers/Thompson_Regional_Collective_Action.pdf	2011	Assisted Translocation	P
Thorpe	Adaptation to Climate Change in Management of Prairie Grasslands	Available at: http://www.cakex.org/sites/default/files/documents/12855-1E12%20Adaptation%20to%20ClimateChange%20in%20Grassland%20Management.pdf	2012	Assisted Migration	R
Thorpe and Stanley	Determining appropriate goals for restoration of imperilled communities and species	Journal of Applied Ecology	2011	Assisted Migration	A
Tishew et al.	Farming for restoration: Building bridges for native seeds	Ecological Restoration	2011	Assisted Migration	A
Tomimatsu	Gene Flow and Dispersal of Plants in Fragmented Landscapes	Available at: http://publications.gc.ca/collections/collection_2012/dn-nd/D3-21-2009-eng.pdf#page=38	2008	Assisted Species Dispersal	R
Tomosy et al.	Role of the U.S. Forest Service. Helping forests, graslands, and wildlife adapt ot shifts in climate.	The Wildlife Professional	2011	Assisted Migration	P

Travis and Dytham	Dispersal and climate change: a review of theory	Dispersal Ecology and Evolution	2012	Assisted Migration	B
Trook	Effects of climate chnage on the distribution of the American Pika (Ochotona princeps) in the Western United States	Available at: http://www.fws.gov/filedownloads/ftp_region6_upload/FOIA%20READING%20ROOM/FOIA%202010/American%20Pika%2012%20Month/12%20month%20status%20review%20citations/Trook%202009.pdf	2009	Assisted Migration	T
Trouwborst	Transboundary Wildlife Conservation in A Changing Climate Adaptation of the Bonn Convention on Migratory Species and Its Daughter Instruments to Climate Change	Diversity	2012	Translocation	A
Trouwborst	Conserving European Biodiversity in a Changing Climate: The Bern Convention, the European Union Birds and Habitats Directives and the Adaptation of Nature to Climate Change	Review of European Community & International Environmental Law	2011	Translocation	A
Twain M	Missouri Pine-Oak Woodlands Restoration Project.	Available at: http://www.fs.fed.us/restoration/CFLR/documents/2010Proposals/Region9/MarkTwain/MTwain_NF_revised_MoPWR_CFLRP_proposal.pdf	2010	Facilitated Dispersal, Translocation	R
**Ukrainetz et al.**	**Comparison of fixed and focal point seed transfer systems for reforestation and assisted migration: a case study for interior spruce in British Columbia.**	**Canadian Journal of Forest Research**	**2011**	**Assisted Migration**	**P**
Urban et al	A crucial step toward realism: responses to climate change	Evolutionary applications	2012	Assisted Migration	A
Utzig et al.	Background Report: Integrated Ecological Impact Assessment - Executive Summary	Available at: http://bcwildfire.ca/ftp/HFP/external/!publish/Wel	2009	Assisted Migration	R
**Valle-Diaz et al.**	**Altitudinal range shift detected through seedling survival of< i> Ceiba aesculifolia</i> in an area under the influence of an urban heat island**	**Forest ecology and management**	**2009**	**Assisted Migration**	**A**
Wallis et al.	Ecosystem, Location, and Climate Effects on Foliar Secondary Metabolites of Lodgepole Pine Populations from Central British Columbia	Journal of Chemical Ecology	2011	Assisted Migration	A
Wallis et al.	Lodgepole pine provenances differ in chemical defense capacities against foliage and stem diseases	Canadian Journal of Forest Research	2010	Assisted Migration	P
van Bruegel	Potential natural vegetation of eastern Africa. Volume 7.	Available at: http://curis.ku.dk/ws/files/36086846/VECEA_Volume7_ProjectedDist.pdf	2011	Human Assisted Migration	R
**Van de Putten**	**Climate Change, Aboveground-Belowground Interactions, and Species' Range Shifts**	**Annual Review of Ecology, Evolution, and Systematics**	**2012**	**Assisted Migration**	**A**
**Van de Veken et al.**	**Experimental assessment of the survival and performance of forest herbs transplanted beyond their range limit**	**Basic and Applied Ecology**	**2012**	**Assisted Migration**	**A**

van den Wollenberg	The future prospects of electronic seed lists	Biodiversity and Conservation	2011	Assisted Migration	A
**Van der Veken et al.**	**Garden plants get a head start on climate change**	**Frontiers in Ecology and the Environment**	**2008**	**Assisted Migration**	**A**
van Wieren	Reintroductions: learning from successes and failures	Restoration Ecology: The new frontier	2012	Assisted Migration, Assisted Colonization	B
**Wang**	**Use of response functions in selecting lodgepole pine**	**Global Change Biology**	**2006**	**Facilited Migration**	**A**
**Wang et al.**	**Integrating environmental and genetic effects to predict responses**	**Ecological applications**	**2010**	**Assisted Migration**	**A**
Varaprasad and Sivaraj	Plant genetic resources conservation and use in light of recent policy developments	Electronic Journal of Plant Breeding	2010	Assisted Migration	A
Vaughn et al.	Restoration Ecology	Nature Education Knowledge	2010	Assisted Migration	P
Webber and Scott	Rapid global change: implications for defining natives and aliens	Global Ecology and Biogeography	2012	Human Assisted Translocation, Managed Relocation, Assisted Migration	A
Weber et al.	Plants in Urban Settings: From Patterns to Mechanisms and Ecosystem Services.	Endlicher et al. (eds.): Perspectives in Urban Ecology: Ecosystems and Interactions Between Humans	2011	Assisted Migration	B
Weeks et al.	Assessing the benefits and risks of translocatoins in changing environments: a genetic perspective	Evolutionary applications	2011	Translocation	A
Vegas-Vilarrubia et al.	Global warming, habitat shifts and potential refugia for biodiversity conservation	Biological conservation	2012	Assisted Migration	A
Wehenkel and Saenz-Romero	Estimating genetic erosion using the example of Picea chihuahuana Martínez	Tree genetics and genomes	2012	Assisted Migration, Assisted Colonization	A
Weisshuhn et al.	Geographic variation in the response to drought in nine grassland species	Basic and applied ecology	2011	Assisted Migration	A
Welbergen et al.	Gap analysis of environmental research needs in the Australian Wet Tropics	Available at: http://www-public.jcu.edu.au/public/groups/everyone/documents/other/jcu_083921.pdf	2011	Assisted Colonisation, Assisted Migration	R
Welch-Devine	Navigating Tradeoffs: Social Sciences at IUCN's World Conservation Congress.	Available at: http://athenaeum.libs.uga.edu/bitstream/handle/10724/11711/welch-devine_meredith_l_200908_ms.pdf?sequence=1	2009	Assisted Migration	T
Wendelberger	Can assessing microsite and regeneration niche preferences when introducing endangered species help mitigate extinction debt?	Available at: http://eco.confex.com/eco/2011/preliminaryprogram/abstract_30380.htm	2011	Assisted Migration	P
West et al.	U.S. Natural Resources and Climate Change: Concepts and Approaches for Management Adaptation	Environmental management	2009	Assisted Migration, Assisted Colonization	A
White	Conservation at large scales: systems of protected areas and protected areas in the matrix	Cole & Yung: Beyond Naturalness: Rethinking Park and Wilderness Stewardship in an Era of …	2010	Assisted Migration	B
White and Shine	The extra-limital spread of an invasive species via 'stowaway'dispersal: toad to nowhere?	Animal Conservation	2009	Human Assisted Translocation	A

Whitlock and Millspaugh	A paleoecologic perspective on past plant invasions in Yellowstone	Western North American Naturalist	2001	Assisted Migration	A
Wiens et al.	Niches, models, and climate change: Assessing the assumptions and uncertainties	Proceedings of the National Academy of Sciences	2009	Assisted Migration	A
Wiensczyk A	Northern Silviculture Committee Winter Conference	Journal of Ecological Monitoring	2012	Assisted Migration	P
Wilczek	Genetic and physiological bases for phenological responses to current and predicted climates	Philosophical transactions of the Royal Society	2010	Assisted Migration	A
Williams	Climatic analogs, climate velocity, and potential shifts in vegetation structure and biomass for Wisconsin under 21st-century climate-change scenarios	Available at: http://www.geography.wisc.edu/faculty/williams/lab/pubs/WilliamsetalEERDFinalReport201206Final.pdf	2012	Managed Relocation	R
Williams	Novel and disappearing climates by 2100AD are concentrated in tropical regions: Implications for conservation biology	Available at: http://eco.confex.com/eco/2007/techprogram/P5205.HTM	2007	Assisted Migration	P
Williams et al.	Queensland's biodiversity under climate change	Available at: http://www.csiro.au/Organisation-Structure/Flagships/Climate-Adaptation-Flagship/Queensland-biodiversity-under-climate-change.aspx	2012	Assisted Migration, Assisted Colonization, Assisted Dispersal	R
Williams et al.	Extrinsic and intrinsic forcing of abrupt ecological change: case studies from the late Quaternary	Journal of Ecology	2011	Assisted Migration	A
**Williams et al.**	**Projected distributions of novel and disappearing climates by 2100 AD**	**Proceedings of the National Academy of Sciences**	**2007**	**Assisted Migration**	**A**
Williams-Tripp et al.	Modeling Rare Species Distribution at the Edge: The Case for the Vulnerable Endemic Pyrenean Desman in France	The Scientific World Journal	2012	Assisted Migration	A
Willis	Climate Change and Conservation	Leader-Williams et al (eds.): Trade-Offs in Conservation: Deciding What to Save	2010	Assisted Colonization	B
**Willis et al**	**Assisted Colonization in a changing climate: a test-study using two U.K. butterflies**	**Conservation Letters**	**2009**	**Assisted Colonization**	**A**
Wilson et al.	Recent evidence for the climate change threat to Lepidoptera and other insects	Journal of insect conservation	2011	Assisted Colonization	A
Winder et al.	Ecological implications for assisted migration in Canadian forests	Forestry Chronicle	2011	Assisted Migration	P
Winston	Science, Practice, and Policy: The Committee on Rare and Endangered	Available at: http://repository.asu.edu/attachments/56395/content/Winston_asu_0010E_10362.pdf	2011	Managed Relocation	T
Visser	Biology: Birds and butterflies in climatic debt	Nature Climate Change	2012	Assisted Migration	P
Visty and Sibold	Project Final Report: Regeneration Status and Dynamics of Rare Ponderosa Pine (Pinus ponderosa)	Available at: http://www.cfc.umt.edu/cesu/NEWCESU/Assets/Individual%20Project%20Reports/NPS%20Projects/CSU/2010/10Sibold_ROMO_ponderosa_pine_final%20rpt.pdf	2011	Assisted Migration	R
**Vitt et al.**	**Assisted Migration: Changes in latitudes, chnages in attitudes**	**Biological conservation**	**2010**	**Assisted Migration**	**A**

**Vitt et al.**	**Assisted migration: part of an integrated conservation strategy**	**Trends in Ecology and Evolution**	**2009**	**Assisted Migration**	**A**
Viveros-Viveros et al.	Variación isoenzimática de Pinus hartwegii Lindl. en un gradiente altitudinal en Michoacán, México	Agrociencia	2010	Assisted Migration	A
**Viveros-Viveros et al.**	**Altitudinal genetic variation in Pinus hartwegii Lindl. I: Height growth, shoot phenology, and frost damage in seedlings**	**Forest ecology and management**	**2009**	**Assisted Migration**	**A**
von Maltitz et al.	Adapting Conservation Strategies to Climate Change in Southern Africa	Leary: Climate Change and adaption	2006	Facilitated Migration, Facilitated Dispersal	B
**Woodall et al.**	**Assessing the potential for urban trees to facilitate forest tree migration in the eastern United States**	**Forest ecology and management**	**2010**	**Assisted Migration**	**A**
Woodhams et al.	Mitigating amphibian disease: strategies to maintain wild populations and control chytridiomycosis	Frontiers in zoology	2011	Managed Relocation	A
Woods et al.	Forest health and climate change: A British Columbia perspective	The Forestry Chronicle	2010	Assisted Migration	P
Woolhouse	The Biology and Ecology of six rare plants from Plumas National Forest, Northern California, USA	Available at: http://scholarworks.sjsu.edu/etd_theses/4221/	2012	Managed Relocation	T
Vose et al.	Effects of climatic variability and change on forest ecosystems: a comprehensive science synthesis for the US	Available at: http://lamar.colostate.edu/~mryan/Publications/Ryan_and_Vose_2012_Effects_of_Climate_Variability_and_Change_Forests_GTRPNW870_Chapter2.pdf	2012	Assisted Migration	R
Wright	Field Staff Perspectives on Managing Climate	Journal of Ecological Monitoring	2012	Assisted Migration	P
Vyse	Broadleaves in the Interior of British Columbia: Their extent, use, management and prospects for investment in genetic conservation and improvement	Forestry Chronicle	2009	Facilited Migration	P
**Yesson**	**A phyloclimatic study of Cyclamen**	**BMC Evolutionary Biology**	**2006**	**Human Assisted Establishment**	**A**
Yung et al.	Experimental Forests and Climate Change	Available at: http://www.fs.fed.us/rm/pubs/rmrs_rp100.pdf	2012	Assisted Migration	R
Yung et al.	A path forward: Conserving Protected Areas in the Context of Global Environmental Change	Cole & Yung: Beyond Naturalness: Rethinking Park and Wilderness Stewardship in an Era of …	2010	Assisted Migration	B
Zellmer	Wilderness, Water, and Climate Change	Environmental law	2012	Assisted Migration, Managed Relocation	A
Zellmer and Anderies	Wilderness preserves: Still relevant and resilient after all these years	Resilience and law	2011	Assisted Migration	P
Zhang et al.	Genetic variation in ecophysiological and survival responses to drought in two native grasses: Koeleria macrantha and Elymus elymoides	Western North American Naturalist	2011	Assisted Migration	A
Ziegler	The Past and Future of White Pine Forests in the Great Lakes Region	Geography compass	2010	Assisted Migration	A
Zimmer	A Radical Step to Preserve a Species: Assisted Migration.	New York Times	2007	Assisted Migration	P

Coming to terms with the concept of moving species threatened by climate change – a systematic review of terminology and definitions. PLOS ONE
Maria H. Hällfors*, Elina M. Vaara, Marko Hyvärinen, Markku Oksanen, Leif E. Schulman, Helena Siipi, Susanna Lehvävirta
*Botany Unit, Finnish Museum of Natural History, P.O. Box 7, FI-00014 University of Helsinki, Finland;
maria.hallfors@helsinki.fi
