# Supplementary material for: Coming to Terms with the Concept of Moving Species Threatened by Climate Change – A Systematic Review of the Terminology and Definitions
Source: PLoS One. 2014 Jul 23;9(7):e102979. doi: 10.1371/journal.pone.0102979 (PMC4108403; doi:10.1371/journal.pone.0102979)
Supplement: Table S2 — Definitions not included in the concept analysis since they focus on the receiving area rather than the unit to be moved. (PDF) [file pone.0102979.s002.pdf]

Supporting Information 2

Table S2. Definitions not included in the concept analysis since they focus on the receiving area rather than the unit to be moved. * = the definition was partially used in the concept analysis as it also focused on the unit.

Source	Definition
Bernazzani et al. 2012 * [1]	"Assisted migration might include moving covered species to new reserves or importing new species that would provide necessary habitat functions for covered species. In both cases, the species being moved is presumed to be adapted to conditions at the recipient site."
Bradley and Wilcove, 2009 [2]	"[…] the translocation of novel species that can survive and reproduce under new climate conditions."
Grady et al., 2011 [3]	"[…] it has been suggested that genotypes best suited to a predicted future climate be preferentially used in ecosystem restoration efforts – termed assisted migration […]."
Ledig et al., 2010 [4]	"This will mean planting with seed or seedlings of nonlocal sources, often assumed to originate from populations to the south or from lower elevation […]. Such management is known as assisted colonization or assisted migration […]."
O'Neill et al., 2008 [5]	"Planting seedlings adapted to a future climate (i.e. assisted migration) is recognized as a key forest management strategy to mitigate negative impacts associated with climate change […]"
St Clair and Howe, 2011 [6]	" Moving species and populations to match future habitats has been called assisted colonization or assisted migration […]."
St Clair and Howe, 2011 [6]	"[…] using assisted colonization to increase genetic diversity by establishing populations adapted to future climates within or adjacent to reserves."
Viveros-Viveros et al., 2009 [7]	"[…] consistent establishing reforestation using seeds of populations with better adaption potential for future global warming that the local ones from a given site […]."
Wang et al, 2009 [8]	"Planting seedlings adapted to predicted future climates has been proposed as a key forest management strategy to mitigate negative impacts of climate change and in some areas, to capitalize on potential opportunities for increased growth associated with warmer climates […]."

References:

1. Bernazzani P, Bradley BA, Opperman JJ (2012) Integrating Climate Change into Habitat Conservation Plans Under the U.S. Endangered Species Act. Environmental Management 49:1103–1114
2. Bradley BA, Wilcove DS (2009) When Invasive Plants Disappear: Transformative Restoration Possibilities in the Western United States Resulting from Climate Change. Restoration Ecology 17:715–721.
3. Grady KC, Ferrier SM, Kolb TE, Hart SC, Allan GJ, et al. (2011) Genetic variation in productivity of foundation riparian species at the edge of their distribution: implications for restoration and assisted migration in a warming climate. Global Change Biology 17:3724–3735.
4. Ledig FT, Rehfeldt GE, Sáenz-Romero C, Flores-López C (2010) Projections of suitable habitat for rare species under global warming scenarios. American journal of botany 97:970–87.
5. O'Neill GAO, Hamann A, Wang T (2008) Accounting for population variation improves estimates of the impact of climate change on species' growth and distribution. Journal of Applied Ecology 45:1040–1049.
6. St.Clair JB, Howe GT (2011) Strategies for conserving forest genetic resources in the face of. Turkish Journal of Botany 35:403–409.
7. Viveros-Viveros H, Sáenz-Romero C, Vargas-Hernández JJ, López-Upton J, Ramírez-Valverde G, et al. (2009) Altitudinal genetic variation in Pinus hartwegii Lindl. I: Height growth, shoot phenology, and frost damage in seedlings. Forest Ecology and Management 257:836–842.
8. Wang T, O'Neill GA, Aitken SN (2010) Integrating environmental and genetic effects to predict responses of tree populations to climate. Ecological applications 20:153-163.

Coming to terms with the concept of moving species threatened by climate change – a systematic review of terminology and definitions. PLOS ONE

Maria H. Hällfors*, Elina M. Vaara, Marko Hyvärinen, Markku Oksanen, Leif E. Schulman, Helena Siipi, Susanna Lehvävirta
*Botany Unit, Finnish Museum of Natural History, P.O. Box 7, FI-00014 University of Helsinki, Finland;
maria.hallfors@helsinki.fi
