# Supplementary material for: Coming to Terms with the Concept of Moving Species Threatened by Climate Change – A Systematic Review of the Terminology and Definitions
Source: PLoS One. 2014 Jul 23;9(7):e102979. doi: 10.1371/journal.pone.0102979 (PMC4108403; doi:10.1371/journal.pone.0102979)
Supplement: Table S3 — Definitions and the analysis units derived from them. (PDF) [file pone.0102979.s003.pdf]

Supporting Information 3

Table S3. Definitions (N=49) and the analysis units derived from them. The analysis units are underneath the main category (Action; Spec. of action [=Specification of action]; What; Spec. of what [=Specification of what]; Where to; Spec. of where to [=Specification of where to]; Motivation; and From where). Indices in superscript refer to the group in which the analysis units were placed:
1. Action: 1.1 Establish; 1.2 Facilitate range shifts; 1.3 Introduce; 1.4 Migrate; 1.5 Mimic natural dispersal; 1.6 Move; 1.7 Relocate; 1.8 Replenish; 1.9 Series of moves; 1.10 Transfer; 1.11 Translocate; 1.12 Release; 1.13 Bring
2. Spec. of action: 2.1 Actively; 2.2 Artificial; 2.3 Assisted; 2.4 Fast enough to track shifting habitats; 2.5 Human agency; 2.6 Intentional; 2.7 Intervening; 2.8 Managed; 2.9 Physical
3. What: 3.1 Ecological entity; 3.2 Genes; 3.3 Individual; 3.4 Population; 3.5 Taxon; 3.6 Unit
4. Spec of what: 4.1 Better adaption in the future; 4.2 Exotic; 4.3 Dispersal limited; 4.4 Threatened; 4.5 Vulnerable to climate change; 4.6 Commercially valuable; 4.7 Native; 4.8 Depleted
5. Where from: 5.1 From current area; 5.2 From degraded area; 5.3 From pre-adapted sources
6. Where to: 6.1 Across landscapes; 6.2 Area; 6.3 Ecosystem; 6.4 Environment; 6.5 Geographic area; 6.6 Habitat; 6.7 Location; 6.8 Range; 6.9 Reserve
7. Spec. of where to: 7.1 Across ecological barriers; 7.2 Beyond the leading edge; 7.3 Climatically suitable; 7.4 Currently unoccupied; 7.5 Other; 7.6 Never occurred; 7.7 No historical occurrence; 7.8 Outside range; 7.9 Suitable; 7.10 Suitable in the future; 7.11 That has only recently become appropriate; 7.12 Within specific area; 7.13 Higher latitude or elevation ;
8. Motivation: 8.1 Anthropogenic threat; 8.2 Biodiversity protection; 8.3 Biodiversity protection under climate change; 8.4 Ecosystem service protection; 8.5 Last resort; 8.6 Mimic distribution change; 8.7 Response to climate change; 8.8. Establish populations; 8.9 Compensation

Source	Definition	Action	Spec. of action	What	Spec. of what	Where from	Where to	Spec. of where to	Motivation
Bernazzi et al., 2012 [1]	"Assisted migration might include moving covered species to new reserves or […]. In both cases, the species being moved is presumed to be adapted to conditions at the recipient site."	moving [1.6]	NA	species [3.5]	covered [4.4]	NA	reserves [6.9]	new [7.8]	NA
Burbridge et al., 2011 [2]	"AC refers to the purposeful movement of species or genes to sites where habitat is predicted to become suitable as the climate changes."	movement [1.6]	purposeful [2.6]	- species [3.5]<br>- genes [3.2]	NA	NA	sites [6.7]	where habitat is predicted to become suitable as the climate changes [7.3]	NA
Camacho, 2010 [3]	"[…] the intentional movement of an organism to an area in which its species has never existed."	movement [1.6]	intentional [2.6]	organism [3.3]	NA	NA	area [6.2]	never existed [7.6]	NA
Camacho, 2010 [3]	"The intentional transfer of flora and fauna to a new region in response to climate change."	transfer [1.10]	intentional [2.6]	- flora [3.1]<br>- fauna [3.1]	NA	NA	region [6.2]	new [7.8]	in response to climate change [8.7]
Carrete and Tella, 2012 [4]	"[…] the translocation of species to previously unoccupied ranges predicted to be favorable for persistence under future climate scenarios (hereafter AC)."	translocation [1.11]	NA	species [3.5]	NA	NA	ranges [6.8]	-previously unoccupied [7.6]<br>-predicted to be favorable for persistence under future climate scenarios [7.10]	NA
Carroll et al., 2009 [5]	"Of course, translocation is controversial and potentially risky when species are moved beyond their natural ranges […]."	- translocation [1.11]<br>- moved [1.6]	NA	species [3.5]	NA	NA	NA	beyond their natural range [7.8]	NA
Chapron and Samelius, 2008 [6]	"[…] proposes moving species outside their historic range to mitigate biodiversity loss induced by climate change."	moving [1.6]	NA	species [3.5]	NA	NA	NA	outside their historic range [7.7]	mitigate biodiversity loss induced by climate change [8.3]
Chen et al., 2009 [7]	"[…] 'assisted migration' of plants highly sensitive to local climatic conditions into areas that have only recently become appropriate."	NA	NA	plants [3.5]	highly sensitive to local climatic conditions [4.5]	NA	areas [6.2]	that have only recently become appropriate [7.11]	NA
Chmura et al., 2011 [8]	"[…] assisted migration, which is the purposeful movement of species or populations to areas where they are expected to be better adapted in the future […]."	movement [1.6]	purposeful [2.6]	- species [3.5]<br>- populations [3.4]	expected to be better adapted in the future [4.1]	NA	areas [6.2]	where they are expected to be better adapted in the future [7.10]	NA
Frascaria-Lacoste & Fernandes-Manjarres, 2012 [9]	"[…] the intentional translocation of populations to compensate for risks related to climate change, is defined broadly as the intentional movement of individuals to reduce risks of extinction that are related to climate change […]"	-translocation [1.11]<br>-movement [1.6]	intentional [2.6]	- populations [3.4]<br>- individuals [3.3]	NA	NA	NA	NA	compensate for risks related to climate change [8.9]
Goodman et al., 2012 [10]	"Introducing endangered species into areas outside of their historic range is a controversial issue […] but one that must be addressed if we are to maintain biodiversity in the face of climate change."	-introducing [1.3]	NA	species [3.5]	endangered [4.4]	NA	areas [6.2]	outside of their historic range [7.7]	maintain biodiversity in the face of climate change [8.3]

Gray et al., 2011 [11]	"[…] assisted movement of endangered species outside their historic range may be necessary for conservation purposes […]."	movement [1.6]	assisted [2.3]	species [3.5]	endangered [4.4]	NA	NA	outside their historic range [7.7]	for conservation purposes [8.2]
Gray et al., 2011 [11]	"We find it useful to differentiate the movement of species far outside their range for conservation purposes (assisted colonization), and population movement within a species range or somewhat beyond the leading edge (assisted migration)."	movement [1.6]	NA	- species [3.5]<br>- population [3.4]	NA	NA	range [6.8]	- within a species range [7.12]<br>- far outside their range [7.8]<br>- somewhat beyond the leading edge [7.2]	for conservation purposes [8.2]
Green at al., 2010 [12]	"Assisted migration is the mediated movement of animals to facilitate or mimic range expansion […] or enhance depleted populations […]. Assisted migration can also replenish extinct or endangered populations, create arks for species under threat and help ameliorate loss of habitat due to climate change […]. Assisted migration generally involves translocating species beyond their existing or former range, to a contiguous environment where barriers to dispersal have occurred through habitat fragmentation, or to an adjacent environment with different temperature regimes […]."	- translocate [1.11]<br>- movement [1.6]<br>- replenish [1.8]	mediated [2.3]	- animals [3.5]<br>- populations [3.4]<br>- species [3.5]	- depleted [4.8]<br>- extinct [4.8]<br>- endangered [4.4]	- their existing range [5.1]<br>their former range [5.1]	environment [6.4]	- beyond their existing range [7.8]<br>- beyond their former range [7.8]<br>- contiguous [7.12]<br>- where barriers to dispersal have occurred through habitat fragmentation [7.1]<br>- adjacent [7.12]<br>- with different temperature regimes [7.3]	- facilitated range expansion [8.6]<br>- mimic range expansion [8.6]<br>- enhance depleted populations [8.2]<br>- create arks for species under threat [8.2]<br>- help ameliorate loss of habitat due to climate change [8.3]<br>- tools for biodiversity restoration [8.2]
Griffiths et al., 2012 [13]	"[…] the intentional movement and release of an organism outside its indigenous range […] to reduce extinction risk."	- movement [1.6]<br>- release [1.12]	intentional [2.6]	organism [3.3]	NA	NA	NA	outside indigenous range [7.8]	to reduce extinction risk [8.2]
Griffiths et al., 2012 [13]	"Assisted colonisation is primarily undertaken to ensure species survival as protection from current or likely future threats is deemed less feasible in its current range than at alternative sites."	NA	NA	NA	NA	NA	sites [6.7]	alternative [7.5]	-ensure species survival [8.2]<br>-protection from current or likely future threats [8.2]
Hansen, 2010 [14]	"All these terms describe an approach aiming at identifying species that are particularly at risk due to climate change and moving them to other, more suited parts of the 'same broad biogeographic region […]."	moving [1.6]	NA	species [3.5]	particularly at risk due to climate change [4.5]	NA	region [6.2]	- other more suited [7.9]<br>- parts of the same biogeographic region [7.12]	aiming at identifying species [8.2]
Hewitt et al., 2011 [15]	"Assisted migration is the intentional translocation or movement of species outside of their historic ranges in order to mitigate actual or anticipated biodiversity losses caused by anthropogenic climate change."	- movement [1.6]<br>- translocate [1.11]	intentional [2.6]	species [3.5]	NA	NA	NA	outside historic range [7.7]	to mitigate actual or anticipated biodiversity losses caused by anthropogenic climate change [8.3]
Hoegh-Guldberg et al., 2008 [16]	"[…] moving species to sites where they do not currently occur or have not been known to occur in recent history […]."	moving [1.6]	NA	species [3.5]	NA	NA	sites [6.7]	- where they do not currently occur [7.4]<br>- where they have not been known to occur in recent history [7.7]	NA
Keel et al., 2011 [17]	"In light of climate change now underway, orchids and other plants must be capable of migrating to higher latitudes, either on their own or with human intervention (= assisted migration)"	migrating [1.4]	human intervention [2.7]	plants [3.5]	NA	NA	higher latitudes [6.5]	NA	climate change [8.7]
Keel et al., 2011 [17]	"[…] assisted migration (= intentional introduction by humans beyond its natural range)"	introduction [1.3]	- intentional [2.6]<br>- by humans [2.5]	NA	NA	NA	NA	beyond natural range [7.8]	NA
Kranabetter et al., 2012 [18]	"Assisted migration of forest trees has been widely proposed as a climate change adaptation strategy, but moving tree populations to match anticipated future climates […]"	moving [1.6]	NA	-forest trees [3.1]<br>-tree populations [3.4]	NA	NA	NA	NA	-climate change adaptation strategy [8.7]<br>- match anticipated future climates [8.7]

Kreyling et al., 2011 [19]	"[…] the intentional movement of focal units (ecotypes, species, taxa, functional types, life forms) to recipient localities, where these focal units are currently absent, and where they cannot be expected to colonize by natural means within a short time frame (i.e. years or decades)."	movement [1.6]	intentional [2.6]	- focal unit [3.6]<br>- ecotypes [3.5]<br>- species [3.5]<br>- taxa [3.5]<br>- functional types [3.1]<br>- life form [3.1]	NA	NA	localities [6.7]	- where currently absent [7.4]<br>- where they cannot be expected to colonize by natural means within a short time frame (i.e. years or decades) [7.1]	NA
Lawler and Olden, 2011 [20]	"Assisted colonization – also known as managed relocation or assisted migration – is one way of facilitating range shifts for species that are restricted in their ability to move in response to climate or other environ- mental changes."	facilitating range shifts [1.2]	NA	species [3.5]	restricted in their ability to move [4.3]	NA	NA	NA	- in response to climate change [8.7]<br>- in response to other environmental changes [8.2]
Lawler and Olden, 2011 [20]	"Assisted colonization involves the intentional translocation of members of a given species to an area outside its native range, to protect it from anthropogenic threats […]."	translocation [1.11]	intentional [2.6]	members of a given species [3.3]	NA	NA	area [6.2]	outside native range [7.8]	to protect it from anthropogenic threats [8.1]
Laws and Kesler, 2012 [21]	"Translocation outside a species historical range […]"	translocation [1.11]	NA	species [3.5]	NA	NA	NA	outside historical range [7.7]	NA
Leech et al., 2011 [22]	"[…] the purposeful movement of species to facilitate or mimic natural population or range expansion."	movement [1.6]	purposeful [2.6]	species [3.5]	NA	NA	NA	NA	- facilitate natural population expansion [8.6]<br>- facilitate range expansion [8.6]<br>- mimic natural population expansion [8.6]<br>- mimic range expansion [8.6]
Leech et al., 2011 [22]	"[…] the purposeful movement of species to facilitate or mimic natural population or range expansion to help ensure forest plantations remain resilient in future climates."	movement [1.6]	purposeful [2.6]	species [3.5]	NA	NA	NA	NA	- facilitate natural population expansion [8.6]<br>- facilitate range expansion [8.6]<br>- mimic natural population expansion [8.6]<br>- mimic range expansion [8.6]<br>- help ensure forest plantations remain resilient in future climates [8.7]
Liu et al., 2012 [23]	"Assisted colonization of endangered species to locations outside their native ranges in response to projected climate change, has emerged as a potential, but highly controversial conservation tool."	NA	NA	species [3.5]	endangered [4.4]	NA	locations [6.7]	outside native ranges [7.8]	- response to projected climate change [8.7]<br>- conservation [8.2]
Liu et al., 2012 [23]	"Assisted colonization, a.k.a. managed relocation, assisted migration, refers to the movement of a species by humans beyond its native range, most notably to habitats at higher latitudes or higher elevations that are predicted to be suitable under future climatic conditions—has recently developed as a conservation concept."	movement [1.6]	by humans [2.5]	species [3.5]	NA	NA	habitats [6.6]	-beyond native range [7.8]<br>-higher latitudes [7.13]<br>-higher elevations [7.13]	- conservation [8.2]
Loss et al., 2011 [24]	"Assisted colonization refers to the physical relocation of a species to a location outside its existing or historical range that is predicted to be favourable for persistence under future climate projections."	relocation [1.7]	physical [2.9]	species [3.5]	NA	NA	location [6.7]	- outside existing range [7.8]<br>- outside historical range [7.7]<br>- predicted to be favourable for persistence under future climate projections [7.3]	NA
McDonald-Madden et al., 2011 [25]	"Managed relocation involves physically moving species from habitat predicted to become unsuitable under climate change to locations where the habitat is predicted to become suitable, but where they have never occurred before."	moving [1.6]	physically [2.9]	species [3.5]	NA	habitat predicted to become unsuitable under climate change [5.2]	location [6.7]	- predicted to become suitable [7.3]<br>- where they have never occurred before [7.6]	- habitat predicted to become unsuitable [8.3]<br>- climate change [8.7]

McIntyre, 2011 [26]	"[…] is commonly posited as the establishment of species beyond their geographical range as a response to climate change […]."	establishment [1.1]	NA	species [3.5]	NA	NA	NA	beyond their geographical range [7.8]	response to climate change [8.7]
McLane and Aitken, 2012 [27]	"The translocation of species into habitable locations outside of their current ranges, termed assisted migration, has been proposed as a means of saving vulnerable species from extinction as a result of climate change."	translocation[1.11]	NA	species [3.5]<br>species [3.5]	vulnerable[4.4]	NA	locations[6.7]	-habitable[7.9]<br>-outside current ranges[7.8]	saving vulnerable species from extinction as a result of climate change[8.3]
McLane and Aitken, 2012 [27]	"One way to avert species losses may be to assist the migration of vulnerable organisms in situations where natural migration is implausible"	migration[1.4]	assist	organisms[3.3]	vulnerable[4.4]	NA	NA	NA	avert species losses[8.2]
Miller et al., 2012 [28]	"Assisted colonization, moving species into regions where they have not previously occurred, aims to establish populations where they are expected to survive as climatic envelopes shift."	moving [1.6]	NA	species [3.5]	NA	NA	regions[6.2]	-where have not previously occurred[7.6]<br>-where expected to survive as climatic envelopes shift[7.3]	establish populations where they are expected to survive as climatic envelopes shift[8.3]
Minteer and Collins, 2010 [29]	"A conservation strategy involving the translocation of species to novel ecosystems in anticipation of range shifts forced by climate change […]."	translocation [1.11]	NA	species [3.5]	NA	NA	ecosystems [6.3]	novel [7.8]	- in anticipation of range shifts forced by climate change [8.3]<br>- conservation [8.2]
Minteer and Collins, 2010 [29]	"[…] relocating threatened species to new locations before their historical ranges become inhospitable due to climate change."	relocating [1.7]	NA	species [3.5]	threatened [4.4]	NA	locations [6.7]	new [7.8]	before their historical ranges become inhospitable due to climate change [8.3]
Moir et al., 2012 [30]	"[…] assisted migration or other movements of threatened host species […]"	movements[1.6]	NA	species [3.5]	threatened [4.4]	NA	NA	NA	NA
Morongiello et al., 2011 [31]	"[…] proactive managed translocation of individuals to areas, either within or outside natural ranges, where conditions are more favourable […]."	translocation [1.11]	- proactive [2.6]<br>- managed [2.8]	individuals [3.3]	NA	NA	areas [6.2]	- within natural range [7.12]<br>- outside natural ranges [7.8]<br>- where conditions are more favourable [7.9]	NA
Morueta-Holme et al., 2010 [32]	"This would involve translocating species to currently unoccupied, but environmentally suitable areas that are likely to remain suitable over the next 100 years or more, in cases where other conservation strategies are unlikely to be sufficient to ensure their survival."	translocating [1.11]	NA	species [3.5]	NA	NA	areas [6.2]	- currently unoccupied [7.4]<br>- environmentally suitable [7.9]<br>- likely to remain suitable over the next 100 years or more [7.10]	in cases where other conservation strategies are unlikely to be sufficient to ensure their survival [8.5]
Mueller and Hellmann, 2008 [33]	"To reduce the risk of extinction due to climate change, some ecologists have suggested human-aided translocation of species, or assisted migration (AM), to areas where climate is projected to become suitable."	translocation [1.11]	human-aided [2.3]	species [3.5]	NA	NA	areas [6.2]	where climate is projected to become suitable [7.3]	- to reduce the risk of extinction due to climate change [8.3]<br>- conservation [8.2]
Olden et al., 2011 [34]	"Conservation of these species may require managed relocation (also called assisted migration or assisted colonization) of individuals to locations where the probability of their future persistence may be higher."	relocation [1.7]	managed [2.8]	- individuals [3.3]<br>- species [3.5]	NA	NA	locations [6.7]	where the probability of their future persistence may be higher [7.10]	conservation [8.2]
Pedlar et al., 2012 [35]	"Assisted migration (AM) is often presented as a strategy to save species that are imminently threatened by rapid climate change."	NA	NA	species [3.5]	imminently threatened by rapid climate change[4.5]	NA	NA	NA	save[8.2]
Pedlar et al., 2012 [35]	"[…] species rescue AM is aimed at conserving species in the face of rapid climate change."	NA	NA	species [3.5]	NA	NA	NA	NA	conserving species in the face of rapid climate change[8.3]

Pedlar et al., 2012 [35]	"[…] forestry AM aims to ensure that plantations of widespread (often commercially valuable) tree species are established using seed sources that will be climatically adapted for the duration of the rotation (i.e., the plantation's planned lifespan […])"	established[1.1]	NA	species [3.5]	commercially valuable[4.6]	seed sources that will be climatically adapted[5.3]	NA	NA	to ensure that plantations of widespread (often commercially valuable) tree species are established[8.8]
Qie 2012 [36]	One proposed conservation tool is assisted colonisation, defi ned by Ricciardi & Simberloff (2009a) as "translocation of a species to favourable habitat beyond their native range to protect them from humaninduced threats". While this encapsulates the practice of moving species outside their distribution range, we note that the concept should be expanded to include "assisted dispersal" (Vitt et al., 2009), that is, introducing species to suitable but unoccupied habitat fragments within their distribution range.	-translocation1.11<br>-moving1.6<br>-introducing1.3	NA	- species [3.5]<br>- species [3.5]	NA	NA	habitat fragments[6.6]	-favourable [7.9]<br>-beyond their native range[7.8]<br>-outside their distribution range[7.8]<br>- suitable[7.9]<br>-within their distribution range[7.12]	-protect them from human induced threat[8.1]<br>- -conservation[8.2]
Regan et al., 2012 [37]	"[…]the transport of individuals by human agency from existing natural habitats to other presently unoccupied habitats predicted on some basis to provide better prospects for future survival."	transport[1.10]	by human agency[2.5]	individuals[3.3]	NA	NA	habitats[6.6]	-predicted on some basis to provide better prospects for future survival[7.10]<br>- other [7.5]<br>-presently unoccupied [7.4]	better prospects for future survival[8.2]
Ricciardi and Simberloff, 2009a [38]	"[…] the translocation of species to favourable habitat beyond their native range to protect them from human-induced threats, such as climate change."	translocation [1.11]	NA	species [3.5]	NA	NA	habitat [6.6]	- favourable [7.9]<br>- beyond native range [7.8]	to protect them from human-induced threats, such as climate change [8.1]
Ricciardi and Simberloff, 2009b [39]	"As stated in our article […] our concern is with the introduction of exotic species (i.e. species moved outside their natural range)."	- moved [1.6]<br>- introduction [1.3]	NA	species [3.5]	exotic [4.2]	NA	NA	outside their natural range [7.8]	NA
Richardson et al., 2009 [40]	"Managed relocation (MR) is an intervention technique aimed at reducing negative effects of climate change on defined biological units such as populations, species, or ecosystems. It involves the intentional movement of biological units from current areas of occupancy to locations where the probability of future persistence is predicted to be higher. The underlying motivation of MR is to reduce the threat of diminished ecosystem services or extinction from climate change."	movement [1.6]	intentional [2.6]	- defined biological units [3.6]<br>- populations [3.4]<br>- species [3.5]<br>- ecosystems [3.1]	NA	from current areas of occupancy [5.1]	locations [6.7]	where the probability of future persistence is predicted to be higher [7.10]	- reducing negative effects of climate change [8.3]<br>- reduce the threat of diminished ecosystem services from climate change [8.4]<br>- reduce the threat of extinction from climate change [8.3]
Richter et al., 2012 [41]	"[…] through the introduction of more drought-tolerant provenances of native species, or by introducing new species better adapted to the future climate of a region."	introduction[1.3]	NA	species [3.5]	-native[4.7]<br>-drought-tolerant[4.1]<br>-new[4.2]<br>-better adapted to future climate[4.1]	NA	NA	NA	NA
Rija et al., 2011 [42]	"[…] translocating the species beyond the current known range within the Udzungwa Mountains, using seedlings found in situ. Assisted migration is increasingly being used as a tool to save species faced with shrinking ecological envelopes owing to the rapidly changing climate."	translocating [1.11]	NA	- species [3.5]<br>- seedlings [3.3]	faced with shrinking ecological envelopes [4.4]	in situ [5.1]	mountain [6.5]	- within [7.12]<br>- beyond the current known range [7.8]	- save species owing to the rapidly changing climate [8.3]
Ruhl, 2010 [43]	"[…] the practice of assisted colonization—actively translocating species from degrading ecosystems to locations more favourable to long-term survival."	translocating [1.11]	actively [2.1]	species [3.5]	NA	from degrading ecosystems [5.2]	locations [6.7]	more favourable to long-term survival [7.10]	NA
Saentz-Romero et al., 2011 [44]	"Assisted migration is a strategy designed to migrate a plant only fast enough to track shifting habitats, and a series of moves will be required to keep the plant within its shifting	- migrate [1.4]<br>- series of moves [1.9]	fast enough to track future habitats [2.4]	plant [3.5]	NA	NA	range [6.8]	within shifting natural range[7.12]	- to track shifting habitats [8.6]<br>- in pace with a changing climate [8.7]

	natural range and in pace with a changing climate […]."								
Saenz-Romero et al., 2012 [45]	"Because the speed of the changing climate is far faster than rates of migration of forest trees […] human-assisted movement of tree populations by massive plantation programs seems inescapable […]"	movement [1.6]	human-assisted[2.5]	tree populations[3.4]	NA	NA	NA	NA	changing climate[8.7]
Safont et al., 2012 [46]	"[…] managed relocation of species to other sites where the required conditions occur naturally. Through this sequence of actions, we could protect against the loss of genetic and species diversity."	NA	NA	species [3.5]	NA	NA	sites[6.7]	-other[7.5]<br>-where the required conditions occur naturally[7.9]	-protect against loss of species diversity[8.2]<br>-protect against loss of genetic diversity[8.2]
Sax et al., 2009 [47]	"[…] aims to save species from the effects of climate change by purposefully transporting them to areas where they have not previously occurred, but where they are expected to survive as temperatures increase."	transporting [1.10]	purposefully [2.6]	species [3.5]	NA	NA	areas [6.2]	- where have not previously occurred [7.7]<br>- where they are expected to survive as temperatures increases [7.3]	save species from the effects of climate change [8.3]
Schwartz et al., 2012 [48]	"The intentional act of moving species, populations, or genotypes to a location outside a known historical distribution for the purpose of maintaining biological diversity or ecosystem functions as an adaptation strategy for climate change."	act of moving[1.6]	intentional[2.6]	-species [3.5]<br>-populations [3.4]<br>-genotypes[3.3]	NA	NA	location[6.7]	outside a known historical distribution[7.7]	-adaptation strategy for climate change [8.7]<br>-maintaining biological diversity[8.2]<br>-maintain ecosystem functions[8.2]
Schwartz et al., 2012 [48]	"Introducing a species into a new location by bringing propagules or individuals and releasing them."	-introducing[1.3]<br>-bringing[1.13]<br>-releasing[1.12]	NA	-species [3.5]<br>-propagules[3.3]<br>-individuals[3.3]	NA	NA	location[6.7]	new[7.8]	NA
Schwartz et al., 2012 [48]	"Assisted migration with the introduction managed to ensure successful establishment."	introduction[1.3]	managed[2.8]	NA	NA	NA	NA	NA	ensure successful establishment[8.8]
Seddon and Soorae, 1999 [49]	"An attempt to establish subspecies for the purpose of conservation outside its recorded distribution but within an appropriate habitat or ecogeographical area"	establish [1.1]	NA	subspecies [3.5]	NA	- NA	- habitat [6.6]<br>- area [6.2]	- outside its recorded distribution [7.7]<br>- within an appropriate [7.9]<br>- within ecogeographical [7.12]	purpose of conservation [8.2]
Seddon et al., 2009 [50]	"[…] growing debate over whether species should be translocated outside their historic ranges to deal with extinction risks as habitats shift due to climate change."	translocated [1.11]	NA	species [3.5]	NA	NA	NA	outside their historic ranges [7.7]	- to deal with extinction risks as habitats shift due climate change [8.3]
Seddon, 2010 [51]	"Translocation of species beyond their natural range to protect them from human-induced threats"	translocation [1.11]	NA	species [3.5]	NA	NA	NA	beyond their natural range [7.8]	to protect them from human induced threats [8.1]
Shirey and Lamberti, 2010 [52]	"We define assisted colonization as the intentional movement of a species or subspecies to a region where it has not occurred in the past, but could occupy under climate change."	movement [1.6]	intentional [2.6]	- species [3.5]<br>- subspecies [3.5]	NA	NA	region [6.2]	- where has not occurred in the recent past [7.4]<br>- could occupy under climate change [7.3]	NA
Stanley-Price, 2010 [53]	"Moving species outside their natural ranges."	moving [1.6]	NA	species [3.5]	NA	NA	NA	outside natural ranges [7.8]	NA
St. Clair and Howe, 2011 [54]	"Move high priority populations to new locations where they are adapted to future climates"	move [1.6]	NA	populations [3.4]	high priority [4.4]	NA	locations [6.7]	- where they are adapted to future climates [7.3]<br>- new [7.8]	NA
St. Clair and Howe, 2011 [54]	"[…] move threatened populations to new environments must be considered as an additional conservation measure."	move [1.6]	NA	populations [3.4]	threatened [4.4]	NA	environments [6.4]	new [7.8]	additional conservation measure [8.2]
Swarts and Dixon, 2009 [55]	"[…] translocation to new locations to mitigate threatening processes, including climate change […]."	translocation [1.11]	NA	NA	NA	NA	locations [6.7]	new [7.8]	- to mitigate threatening processes [8.2]<br>- climate change [8.7]
Swarts and Dixon, 2009 [55]	"[…] artificial assistance to migrate from hostile environments, across ecological barriers to new climatically buffered sites."	assistance to migrate [1.4]	artificial [2.2]	NA	NA	from hostile environments [5.2]	environments [6.4]	- new [7.8]<br>- across ecological barriers [7.1]<br>- climatically buffered [7.3]	NA

Thomas, 2011 [56]	"Translocating species (i.e. assisted colonisation or assisted migration) beyond their recorded native ranges is an option when traditional strategies are insufficient."	translocating [1.11]	NA	species [3.5]	NA	NA	NA	beyond their recorded native ranges [7.7]	is an option when traditional strategies are insufficient [8.5]
van der Veken et al., 2008 [57]	"To what degree should humans intervene to prevent extinctions by transporting species to location where suitable conditions exists?"	transporting [1.10]	humans intervene [2.7]	species [3.5]	NA	NA	location [6.7]	where suitable conditions exists [7.9]	prevent extinctions [8.2]
van der Veken et al., 2012 [58]	"[…] plant species introductions for conservation purposes beyond current range boundaries in the face of climate change. The assisted translocation of populations, species or ecosystems towards higher altitudes and latitudes might be an option to mitigate the effects of climate change and preserve future biodiversity."	-introductions[1.3]<br>-translocations[1.11]	NA	-populations[3.4]<br>-species[3.5]<br>-ecosystems[3.1]	NA	NA	NA	-higher latitudes[7.13]<br>-higher altitudes[7.13]	-conservation purposes[8.2]<br>-climate change[8.7]<br>-mitigate the effects of climate change [8.7]<br>-preserve future biodiversity[8.2]
Vitt et al., 2009 [59]	"We envisage AM as having the role that mimics the natural dispersal of some species across landscapes, tracking the leading edge of their shifting bioclimatic envelopes, and it should be undertaken only if species is not capable of natural migration, plastic response or adaption in situ."	mimics natural dispersal [1.5]	NA	species [3.5]	NA	NA	across landscapes [6.1]	tracking the leading edge of their shifting bioclimatic envelope [7.3]	- should be undertaken only if species is not capable of natural migration [8.2]<br>- […] not capable plastic response [8.2]<br>- […] not capable adaption in situ [8.2]
Vitt et al., 2010 [60]	"In this paper, we limit the definition of assisted migration to the purposeful movement of species to facilitate or mimic natural range expansion, as a direct management response to climate change."	movement [1.6]	purposeful [2.6]	species [3.5]	NA	NA	NA	NA	- facilitate natural range expansion [8.6]<br>- mimic natural range expansion [8.6]<br>- management response to climate change [8.7]
									-

Refrences:

1. Bernazzani P, Bradley BA, Opperman JJ (2012) Integrating Climate Change into Habitat Conservation Plans Under the U.S. Endangered Species Act. Environmental Management 49:1103–1114
2. Burbidge AA, Byrnes M, Coates D, Garnett ST, Harris S, et al. (2011) Is Australia ready for assisted colonization? Policy changes required to facilitate translocations under climate change. Pacific Conservation Biology 17:259–269.
3. Camacho AE (2010) Assisted Migration: Redefining Nature and Natural Resource Law Under Climate Change. Yale Journal on Regulation 27:171–255.
4. Carrete M, Tella JL (2012) Is assisted colonization feasible? Lessons from past introductions. Frontiers in Ecology and the Environment 10:12-13.
5. Carroll MJ, Anderson BJ, Brereton TM, Knight SJ, Kudrna O, et al. (2009) Climate change and translocations: The potential to re-establish two regionally-extinct butterfly species in Britain. Biological Conservation 142:2114–2121.
6. Chapron G, Samelius G (2008) Where species go, legal protection must follow. Science 14:1049.
7. Chen J, Cannon CH, Hu H (2009) Tropical botanical gardens: at the in situ ecosystem management frontier. Trends in plant science 14:584–9.
8. Chmura DJ, Anderson PD, Howe GT, Harrington CA, Halofsky JE, et al. (2011) Forest responses to climate change in the northwestern United States: Ecophysiological foundations for adaptive management. Forest Ecology and Management 261:1121–1142.
9. Frascaria-Lacoste N, Fernández-Manjarres J (2012) Assisted Colonization of Foundation Species: Lack of Consideration of the Extended Phenotype Concept - Response to Kreyling et al. (2011). Restoration Ecology 20(3):296–298.
10. Goodman J, Maschinski J, Hughes P, McAuliffe J, Roncal J, et al. (2012) Differential response to soil salinity in endangered key tree cactus: implications for survival in a changing climate. PLOS ONE, 7(3):e32528.
11. Gray LK, Gylander T, Mbogga MS, Chen P-Y, Hamann A (2011) Assisted migration to address climate change: recommendations for aspen reforestation in western Canada. Ecological applications 21:1591–603.
12. Green BS, Gardner C, Linnane A, Hawthorne PJ (2010) The good, the bad and the recovery in an assisted migration. PLOS ONE 5:e14160.
13. Griffiths CJ, Zuël N, Tatayah V, Jones CG, Griffiths O (2012) The Welfare Implications of Using Exotic Tortoises as Ecological Replacements. PLOS ONE 7(6): e39395.
14. Hansen DM (2010) On the use of taxon substitutes in rewilding projects on islands. Islands and Evolution 19:111–146.
15. Hewitt N, Klenk N, Smith AL, Bazely DR, Yan N, et al. (2011) Taking stock of the assisted migration debate. Biological Conservation 144:2560–2572.
16. Hoegh-Guldberg O, Hughes L, Mcintyre S, Lindenmayer DB, Parmesan C, et al. (2008) Assisted Colonization and Rapid Climate Change. Science 321:345–346.
17. Keel BG, Zettler LW, Kaplin BA (2011) Seed Germination of Habenaria repens (Orchidaceae) in situ Beyond its Range, and its Potential for Assisted Migration Imposed by Climate Change. Castanea 76:43–54.
18. Kranabetter JM, Stoehr MU, O'Neill GA (2012) Divergence in ectomycorrhizal communities with foreign Douglas-fir populations and implications for assisted migration. Ecological applications 22(2):550–60.
19. Kreyling J, Bittner T, Jaeschke A, Jentsch A, Steinbauer JM, et al. (2011) Assisted Colonization: A Question of Focal Units and Recipient Localities. Restoration Ecology 19:433–440.
20. Lawler JJ, Olden JD (2011) Reframing the debate over assisted colonization. Frontiers in Ecology and the Environment, 9(10), 569–574.
21. Laws RJ, Kesler DC (2012) A Bayesian network approach for selecting translocation sites for endangered island birds. Biological Conservation 155:178-185.
22. Leech SM, Almuedo PL, Neill GO (2011) Assisted Migration : Adapting forest management to a changing climate. BC Journal of Ecosystem and Management 12:18–34.

23. Liu H, Feng C, Chen B, Wang Z, Xie X, et al. (2012) Overcoming extreme weather challenges: Successful but variable assisted colonization of wild orchids in southwestern China. Biological Conservation 150:68–75.

24. Loss SR, Terwilliger LA, Peterson AC (2011) Assisted colonization: Integrating conservation strategies in the face of climate change. Biological Conservation 144:92–100.

25. McDonald-Madden E, Runge MC, Possingham HP, Martin TG (2011) Optimal timing for managed relocation of species faced with climate change. Nature Climate Change 1:261–265.

26. McIntyre S (2011) Ecological and anthropomorphic factors permitting low-risk assisted colonization in temperate grassy woodlands. Biological Conservation 144:1781–1789.

27. McLane SC, Aitken SN (2012) Whitebark pine (Pinus albicaulis) assisted migration potential: testing establishment north of the species range. Ecological applications 22(1):142–53.

28. Miller KA, Miller HC, Moore JA, Mitchell NJ, Cree A, et al. (2012) Securing the Demographic and Genetic Future of Tuatara through Assisted Colonization. Conservation biology 26(5):790–798.

29. Minteer BA, Collins JP (2010) Move it or lose it? The ecological ethics of relocating species under climate change. Ecological Applications 20:1801–1804.

30. Moir ML, Vesk PA, Brennan KEC, Hughes L, Keith DA, et al. (2012) A preliminary assessment of changes in plant-dwelling insects when threatened plants are translocated. Journal of Insect Conservation 16(3):367-277

31. Morrongiello JR, Beatty SJ, Bennett JC, Crook DA, Ikedife DNEN, et al. (2011) Climate change and its implications for Australia's freshwater fish. Marine and Freshwater Research 62:1082-1098

32. Morueta-Holme N, Fløjgaard C, Svenning J-C (2010) Climate change risks and conservation implications for a threatened small-range mammal species. PLOS ONE 5:e10360.

33. Mueller JM, Hellmann JJ (2008) An assessment of invasion risk from assisted migration. Conservation biology 22:562–7.

34. Olden JD, Kennard MJ, Lawler JJ, Poff NL (2011) Challenges and opportunities in implementing managed relocation for conservation of freshwater species. Conservation biology 25:40–7.

35. Pedlar JH, Mckenney DW, Aubin I, Beardmore T, Beaulieu J, et al. (2012) Placing Forestry in the Assisted Migration Debate. BioScience 62:835–842.

36. Qie L, Howard SD, Lim SL-H, Sodhi NS (2012) Assisted dispersal of tropical dung beetles. The Raffles Bulletin of Zoology 25:155-160

37. Regan HM, Syphard AD, Franklin J, Swab RM, Markovchick L, et al. (2012) Evaluation of assisted colonization strategies under global change for a rare, fire-dependent plant. Global Change Biology, 18(3):936–947.

38. Ricciardi A, Simberloff D (2009a) Assisted colonization is not a viable conservation strategy. Trends in ecology & evolution 24:248–53.

39. Ricciardi A, Simberloff D (2009b) Assisted colonization: good intentions and dubious risk assessment. Trends in Ecology & Evolution 24:476–477.

40. Richardson DM, Hellmann JJ, McLachlan JS, Sax DF, Schwartz MW, et al. (2009) Multidimensional evaluation of managed relocation. Proceedings of the National Academy of Sciences of the United States of America 106:9721–4.

41. Richter S, Kipfer T, Wohlgemuth T, Calderón Guerrero C, Ghazoul J, et al. (2012) Phenotypic plasticity facilitates resistance to climate change in a highly variable environment. Oecologia, 169(1):269–79.

42. Rija AA, Mwamende KA, Hassan SN (2011) The aftermath of environmental disturbance on the critically endangered Coffea kihansiensis in the Southern Udzungwa mountains, Tanzania. Tropical Conservation Science 4:359.

43. Ruhl JB (2010) Assisted Colonization: Facilitate Migration First. Science 330:1317–1318.

44. Sáenz-Romero C, Beaulieu J, Rehfeldt GE (2011) Altitudinal genetic variation among Pinus patula populations from Oaxaca, México, in growth chambers simulating global warming temperetures. Agrosciencia 45:399–411.

45. Sáenz-Romero C, Rehfeldt GE, Duval P, Lindig-Cisneros RA (2012) Abies religiosa habitat prediction in climatic change scenarios and implications for monarch butterfly conservation in Mexico. Forest Ecology and Management 275:98-106.

46. Safont E, Vegas-Vilarrúbia T, Rull V (2012) Use of Environmental Impact Assessment (EIA) tools to set priorities and optimize strategies in biodiversity conservation. Biological Conservation 149:113-121.

47. Sax DF, Smith KF, Thompson AR (2009) Managed relocation: a nuanced evaluation is needed. Trends in ecology & evolution 24:472–473.

48. Schwartz MW, Hellmann JJ, McLachlan JM, Sax DF, Borevitz JO, et al. (2012) Managed Relocation : Integrating the Scientific, Regulatory, and Ethical Challenges. BioScience 62:732–743.

49. Seddon PJ, Soorae PS (1999) Guidelines for Subspecific Substitutions in Wildlife Restoration Projects. Conservation Biology 13:177–184.

50. Seddon PJ, Armstrong DP, Soorae P, Launay F, Walker S, et al. (2009) The risks of assisted colonization. Conservation biology 23:788–9.

51. Seddon PJ (2010) From Reintroduction to Assisted Colonization: Moving along the Conservation Translocation Spectrum. Restoration Ecology 18:796–802.

52. Shirey PD, Lamberti GA (2010) Assisted colonization under the U.S. Endangered Species Act. Conservation Letters 3:45–52.

53. Stanley Price MR (2010) Assisted Colonization: Move ahead with models. Science 330:1317.

54. St.Clair JB, Howe GT (2011) Strategies for conserving forest genetic resources in the face of. Turkish Journal of Botany 35:403–409.

55. Swarts ND, Dixon KW (2009) Terrestrial orchid conservation in the age of extinction. Annals of botany 104:543–56.

56. Thomas CD (2011) Translocation of species, climate change, and the end of trying to recreate past ecological communities. Trends in ecology & evolution 26:216–21.

57. van der Veken S, Hermy M, Vellend M, Knapen A, Verheyen K (2008) Garden plants get a head start on climate change. Frontiers in Ecology and the Environment 6:212–216.

58. van der Veken S, De Frenne P, Baeten L, Van Beek E, Verheyen K, et al. (2012) Experimental assessment of the survival and performance of forest herbs transplanted beyond their range limit. Basic and Applied Ecology, 13(1):10–19.

59. Vitt P, Havens K, Hoegh-Guldberg O (2009) Assisted migration: part of an integrated conservation strategy. Trends in ecology & evolution 24:473–4

60. Vitt P, Havens K, Kramer AT, Sollenberger D, Yates E (2010) Assisted migration of plants: Changes in latitudes, changes in attitudes. Biological Conservation 143:18–27.

Maria H. Hällfors*, Elina M. Vaara, Marko Hyvärinen, Markku Oksanen, Leif E. Schulman, Helena Siipi, Susanna Lehvävirta
*Botany Unit, Finnish Museum of Natural History, University of Helsinki, Helsinki Finland; maria.hallfors@helsinki.fi
